# Characterization of twenty-five ovarian tumour cell lines that phenocopy primary tumours

**DOI:** 10.1038/ncomms8419

**Published:** 2015-06-17

**Authors:** Tan A. Ince, Aurea D. Sousa, Michelle A. Jones, J. Chuck Harrell, Elin S. Agoston, Marit Krohn, Laura M. Selfors, Wenbin Liu, Ken Chen, Mao Yong, Peter Buchwald, Bin Wang, Katherine S. Hale, Evan Cohick, Petra Sergent, Abigail Witt, Zhanna Kozhekbaeva, Sizhen Gao, Agoston T. Agoston, Melissa A. Merritt, Rosemary Foster, Bo R. Rueda, Christopher P. Crum, Joan S. Brugge, Gordon B. Mills

**Affiliations:** 1 Department of Pathology, Interdisciplinary Stem Cell Institute, Braman Family Breast Cancer Institute, and Sylvester Comprehensive Cancer Center, Miller School of Medicine, University of Miami, Miami, Florida 33136, USA; 2 Lineberger Comprehensive Cancer Center, University of North Carolina, Chapel Hill, North Carolina 27514, USA; 3 Department of Pathology, Brigham and Women’s Hospital, Harvard Medical School, Boston, Massachusetts 02115, USA; 4 Department of Systems Biology, MD Anderson Cancer Center, Houston, Texas 77030, USA; 5 Department of Cell Biology, Harvard Medical School, Boston, Massachusetts 02115, USA; 6 Department of Bioinformatics and Computational Biology, MD Anderson Cancer Center, Houston, Texas 77030, USA; 7 Department of Molecular and Cellular Pharmacology, University of Miami Miller School of Medicine, Miami, Florida 33136, USA; 8 Vincent Center for Reproductive Biology, Division of Gynecologic Oncology, Department of Obstetrics and Gynecology, Massachusetts General Hospital and Harvard Medical School, Boston, Massachusetts 02114, USA

## Abstract

Currently available human tumour cell line panels consist of a small number of lines in each lineage that generally fail to retain the phenotype of the original patient tumour. Here we develop a cell culture medium that enables us to routinely establish cell lines from diverse subtypes of human ovarian cancers with >95% efficiency. Importantly, the 25 new ovarian tumour cell lines described here retain the genomic landscape, histopathology and molecular features of the original tumours. Furthermore, the molecular profile and drug response of these cell lines correlate with distinct groups of primary tumours with different outcomes. Thus, tumour cell lines derived using this methodology represent a significantly improved platform to study human tumour pathophysiology and response to therapy.

## Abstract (web summary)

Despite methodological improvements, it remains extremely difficult to routinely establish cell lines from human primary tumours. Here, the authors develop a culture medium that allows efficient cell line establishment and use this to generate 25 ovarian cancer cell lines.

## Graphical abstract

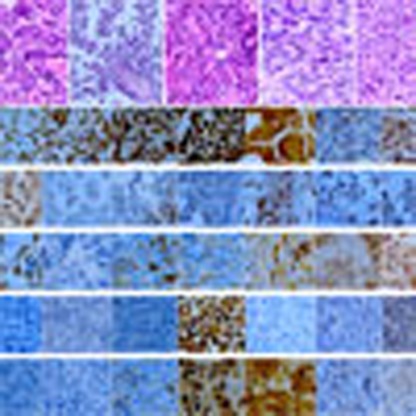

More than 60 years have passed since the establishment of the first human cancer cell line, HeLa, in 1951 (ref. 1). Since then, human tumour cell lines have had an extremely important impact on cancer research and greatly facilitated development of a variety of cancer treatments that benefit human patients^2^,^3^,^4^.

Human carcinomas that grow uncontrollably in the body are often paradoxically difficult to grow in cell culture. A robust and efficient cell line model system that predicts patient response to various drugs would greatly improve development and implementation of new drugs for personalized treatment of cancer patients.

Despite many decades of improvements in methods for establishing cancer cell lines^3^, it remains extremely difficult to routinely establish high-quality, permanent cell lines from human primary tumours with high efficiency, limiting the number and diversity of cell lines available for study. Moreover, in many tumour types, only high-grade subtypes have yielded cell lines, resulting in collections that do not accurately reflect the true spectrum of tumours encountered in the clinic.

Further, many of the tumour cell lines available are of uncertain origin due to the lack of ‘fingerprinting’ technology able to ascertain identity when the lines were developed. In addition, the original tumour is not available for analysis with modern technology such as next-generation sequencing. Thus, a more efficient method of establishing human tumours as cultures that reflect the heterogeneity of human tumours is highly desirable and could offer a more effective platform for drug discovery.

The challenges associated with establishing ovarian carcinoma cell lines is illustrated by a comprehensive study, which reported that only 11 out of 90 tumour samples could be established as cell lines^5^. The 90 tumour samples were collected from 67 patients with 44 serous, 2 clear-cell, 2 endometrioid, 3 mixed mullerian and 16 not otherwise specified adenocarcinomas. All the successful cell lines were from high-grade poorly differentiated cancers with serous (*n*=4), not otherwise specified (*n*=4) or mixed mullerian (*n*=1) histology. Attempts to establish cell lines from clear-cell and endometrioid cancers, or from moderately differentiated carcinomas were not successful. Importantly, it took >5 months for the first passage, and the cell lines could be propagated only up to 15 passages^5^. In addition, all successful cell lines were established from ascites fluid, not from solid tumours. These limitations suggest a strong selection pressure in culture that limits the ability of standard cancer cell line panels to accurately reflect the diversity of human tumours. This has caused some to question whether the tumour cell lines established in routine culture media are truly representative of human clinical tumours, and whether this limits their utility in understanding tumour biology and facilitating drug development^6^,^7^.

The difficulty of establishing human tumour cell lines extends to many other tumours types as well. For example, since the first breast cancer cell line was established^8^, only a small number of additional cell lines have been developed^4^. Cailleau *et al.*^9^ reported a 10% success rate from metastatic tumours, and 0% for 300 primary tumours. Amadori *et al.*^10^ established only 1 cell line out of 136 primary tumours. McCallum *et al.*^11^ established 10 cell lines out of 135 primary tumours. Gazdar *et al.*^12^ established cell lines in 10% of the cases (18/177). However, all of these cell lines had exceedingly long doubling times (16–60 days) and a long lag time before the first cell passage (4–44 months), limiting their usefulness^12^.

These experiences provide clear indication that the great diversity of tumour subtypes and patient groups are not adequately represented in the existing tumour cell line collections. Consistent with this view, comparison of copy-number changes, mutations and messenger RNA (mRNA) expression profiles revealed pronounced differences between commonly used ovarian cancer cell lines and ovarian cancer tumour samples^13^. Thus, not only a very small fraction of human tumours can be cultured, but these cell lines also appear to lose the characteristics of the original tumour.

In general, the standard cell culture media such as DMEM, Ham’s F12, Roswell Park Memorial Institute medium (RPMI), McCoy’s and MCDB105 are composed of 40–50 ingredients. Because they are unable to support living cells on their own, these media are supplemented with tissue extracts, feeder layers, anti-apoptotic drugs and serum. While these supplements have improved culture results^14^,^15^, they also have various drawbacks. At a minimum, the presence of anti-apoptotic drugs complicates cytotoxic screens, tissue extracts introduce biological variability and feeder layers may confound cytostatic assays.

Hence, we set out to develop a highly efficient culture system that preserves the phenotype of the original tumour as much as possible for common human cancers, driven both by the clear need for improved *in vitro* models and by the encouraging results with a chemically defined culture medium that we described previously^16^. This medium has over 80 ingredients that provide all the essential nutrients for maintaining basic cellular metabolism without undefined supplements such as serum, pituitary extract, feeder layers, conditioned medium or drugs^16^. As a result, in this medium normal human breast epithelial cells maintain telomerase activity^17^ and proliferate beyond 70 population doublings, a nearly 10^21^-fold expansion of cell numbers, in contrast with the 10–15 doublings in the standard medium^16^. More recently, we were also able to culture normal ovarian and fallopian tube epithelium in a modified version of this medium^18^. These results encouraged us to hypothesize that perhaps human tumours could also be grown consistently in such a medium.

Here, we report the phenotypic properties of 25 new ovarian cancer cell lines (OCI) derived using cell culture media optimized for human ovarian cancer subtypes (Ovarian Carcinoma Modified Ince medium, OCMI). This panel of cell lines will be available to the research community and nearly doubles the number of existing 26 cell lines available from public cell lines repositories, such as ATCC and ECACC.

## Results

### Tumour cells fail to thrive in standard cell culture media

**Figure 1:**
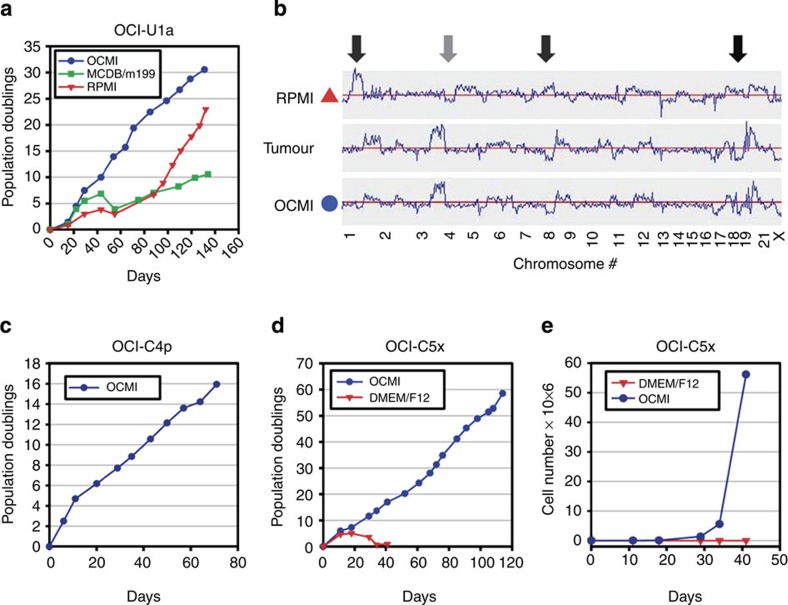
Long-term culture of ovarian tumours in OCMI media. (**a**) Cells from malignant ascites fluid-containing ovarian carcinoma cells (OCI-U1a) were plated in OCMI (blue line), MCDB105/M199 with 10% serum (green line) or RPMI-1640 with 10% serum (red line). A significant decrease in population doubling is observed in MCDB105/M199 and RPMI-1640 media after 7 weeks (green and red lines). This was not observed in OCMI, in which the cells reached 30 population doublings at cessation of the experiment. A fast proliferating cell population emerged in RPMI-1640 medium around day 90 (red line) and was established as a permanent cell line. (**b**) The whole-genome DNA copy-number variants (CNVs) of the OCI-U1a tumour cells cultured in OCMI and RPMI and from the paired uncultured tissue were examined using a 250-K SNP array. The CNV trace reveals several peaks that are gained (amplifications, black arrows) and lost (deletions, grey arrows) in tumour cells cultured in RPMI (top trace) compared with the uncultured tumour sample (middle trace). In contrast, the CNV pattern of tumour cells cultured in OCMI appears significantly more similar to the uncultured tumour (bottom and middle CNV traces, respectively). (**c**,**d**) The ovarian clear-cell lines OCI-C4p and OCI-C5x were cultured to at least 60 and 100 population doublings, respectively, in OCMI (blue line). In the control medium (DMEM:F12 with 10% serum) the cells stopped proliferating in 30–40 days (red line). (**e**) Total cell numbers were plotted instead of population doublings to highlight the scale difference in OCMI (blue line) versus DMEM:F12 with 10% serum (red line) depicted in **d**. See Supplementary Methods, Supplementary Figs 1–6 and Supplementary Tables 1–7 for more detailed cell line and tumour information.

In standard culture media we were able to establish tumour cell lines with a success rate of <1%, consistent with prior reports. In the single successful case, the ovarian tumour line OCI-U1a is derived in RPMI medium (Fig. 1a), in which a brief period of rapid proliferation (days 0–20) is followed by growth arrest (days 20–40), widespread cell death (days 40–50) and the eventual emergence of a rapidly proliferating rare clone that give rise to a continuous cell line (days 60–90).

Importantly, the DNA copy-number variations (CNVs) identified in the cell line that is established in RPMI differ significantly from those found in the uncultured tumour tissue, consistent with clonal outgrowth of select subpopulations or the acquisition of additional genetic aberrations during tissue culture (Fig. 1b). Consistent with the experience of others in this field, this was the only tumour specimen that yielded a continuous ovarian tumour cell line using standard media over the course of our nearly 10-year-long study.

### High-efficiency establishment of ovarian cell lines

To derive a representative set of human ovarian cancer cell lines from multiple types of ovarian tumours, we tested many culture conditions over 10 years and developed a medium (OCMI) that allowed establishment of 25 new ovarian cancer cell lines in 26 attempts. Through a combination of theoretical deduction of metabolic pathways and trial and error, we found a mixture of variables with a large synergistic effect on culture efficiency^16^.

In many cases, the individual components had incremental effects that emerged over many passages, varied between different histotypes and patients and produced combined effects that were non-obvious and synergistic, which made optimization difficult (Supplementary Figs 1–4). Also, empirically testing all possible combinations was prohibitive due to very large number of permutations (Supplementary Note 1). Nevertheless, we were able to find a formulation that has a high success rate across the spectrum of ovarian tumours.

We refer to the ovarian carcinoma cell lines cultured in OCMI medium as OCI lines, to distinguish them from standard ovarian carcinoma (SOC) lines (Supplementary Tables 1 and 2). In OCMI medium, the tumour cells are able to proliferate immediately without significant *in vitro* clonal selection or a need to acquire additional genomic or epigenetic aberrations (Fig. 1). Furthermore, it is possible to culture these cells continuously for >20–100 passages (∼60–300 population doublings) with no decrease in growth rate; we have not yet identified an upper limit of population doublings (Fig. 1; Supplementary Table 3).

The capital letter after the ovarian carcinoma designation ‘OCI’ refers to the histological subtype of the original tumour: papillary serous, clear cell, endometrioid and mucinous cancers, and rare types such as carcinosarcoma and dysgerminoma. Together, the papillary serous, clear cell, endometrioid and mucinous subtypes account for >90% of ovarian adenocarcinomas; accordingly this panel of cell lines is broadly representative of ovarian cancer. The number after the histotype designation refers to the unique case number.

The lower case letter at the end of each cell line name refers to tissue source; 14 of the cell lines were established from primary solid tumours, 7 from ascites fluid and 4 from primary mouse xenografts derived from direct implantation of human tumours into immunocompromised mice. All 25 OCI lines formed soft agar colonies consistent with retention of a transformed phenotype in culture (Supplementary Table 2). In two cases, the bulk of the tumour was located in the fallopian tubes—these cell lines are referred to as ‘FCI’ cell lines.

Adenocarcinoma of the ovary is a heterogeneous disease that is comprised of many histopathological subtypes with distinct features. In many cases, the original subtype of tumour that was used to establish the ‘SOC’ cell lines is unknown^13^. Hence, in this study we tried to use the small subset of SOC cell lines in which the original histologic subtype is known (Supplementary Table 1).

### Standard media fail to support OCI cell lines

We observed that none of the OCI lines we tested can be efficiently cultured in existing standard media (Supplementary Fig. 5a–d; Supplementary Table 4). In contrast, all of the SOC lines we tested can be cultured in OCMI medium (Supplementary Fig. 5e–h).

The SOC lines are cultured in a variety of different standard media, and no single standard medium appears to support culture of all existing SOC lines, making it difficult to compare a large panel of SOC lines with one another because they require different media. Our results indicate that OCMI medium has the potential to serve as a universal culture medium for SOC lines, facilitating comparisons across cell lines.

We also observed that OCI cell lines cannot be efficiently cultured in the medium recently described by Watanabe *et al.,* who used an apoptosis inhibitor (Y-27632) that inactivates Rho-associated kinase (ROCK) to help culture human embryonic stem cells^19^,^20^. This medium was later used to culture several human tumours^14^; however, our results indicate that Y-27632 has a negative effect on OCI lines (Supplementary Fig. 6). Interestingly, it was recently shown that the Y-27632 also negatively affects the survival of CD34+ haematopoietic progenitor cells, human adipose-derived stem cells and proliferation of melanomas^21^,^22^,^23^, suggesting that it has differential impact on different lineages and tissue-specific stem cells.

### OCI cell lines mirror the genomic landscape of tumours

Major genetic alterations may accumulate during cell culture in standard media^24^. To compare tumour versus cell line genomes, we examined their loss-of-heterozygosity (LOH) profiles and found that the majority of OCI cell lines exhibit very high levels of identity to their corresponding uncultured tumour sample (Supplementary Figs 7 and 8). We had frozen primary tumour samples available in 16 cases, which we used for the LOH analysis. Among these, 14 cases have especially striking similarities between the cell line and tumour with a >95% identity between the LOH pattern of the uncultured tumour and the matching cell lines (Supplementary Fig. 8; Supplementary Data 1). In two cases, the similarity between the cell line and the tumour is 90–95%. It is possible that intra-tumoral genetic heterogeneity may be responsible for some of the small differences between cultured cells and the primary tumour, because the fragment of tumour from which the cell line was established is necessarily different than the fragment of tumour from which the DNA was isolated^25^,^26^,^27^. It is also possible that in a few cases some of the changes may be due to accumulation of genetic alterations during culture, even though there was no noticeable difference in the growth rate among these lines and the other OCI lines. In any case, there is a high overall match between the LOH patterns of the primary tumours and OCI lines. We were not able to compare the DNA of the SOC lines with the matched original tumour DNA, because these cells were established decades ago and the original tumour sample is not available (Supplementary Table 1).

Next, we compared CNV patterns of OCI cell lines with the ovarian tumours analysed in the TCGA data set^28^. Consistent with the LOH analysis, we found that the overall CNV trends of the OCI lines are similar to primary tumour samples (Supplementary Fig. 9).

A persistent problem in the cell culture field has been cross-contamination and misidentification of up to 15–20% of the cell lines^29^. The close genomic match between OCI lines and the original tumour tissues ensure that each OCI line is derived from a unique patient. Furthermore, we sequenced the mitochondrial DNA and short tandem repeats of the OCI and SOC lines to provide a permanent unique identifier for authentication of these cell lines (Supplementary Table 6). Overall, these results indicate that OCI cell lines faithfully preserve the genetic alterations present in the original tumour.

### OCI and SOC lines have different mRNA signatures

**Figure 2:**
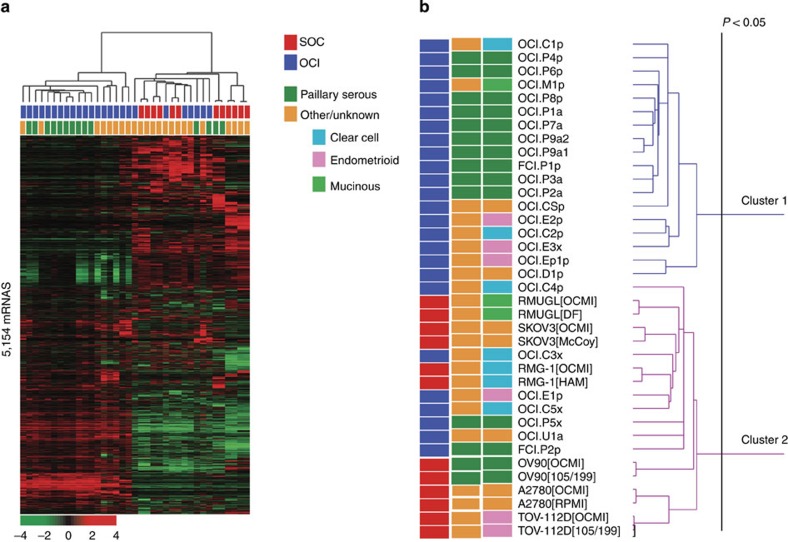
mRNA profiling of OCI cell lines identifies two major clusters. (**a**) Unsupervised hierarchical clustering of mRNA expression levels of OCI (blue bars) and SOC (red bars) ovarian cancer cell lines reveal two clusters. Genes with ≥2-fold difference relative to the median value in at least four cell lines were selected for hierarchical clustering analysis (5,146 gene features). Rows represent individual genes and columns represent each cell line. The red and green colours reflect relative high and low expression levels, respectively, as indicated in the scale bar (log2-transformed scale). Two major clusters are observed: cluster 1 contains only OCI cell lines (left cluster, blue only), and cluster 2 contains a mixture of SOC and OCI cell lines (right cluster, red and blue). While the papillary serous histotype almost exclusively align within cluster 1 (green bars), the other subtypes are present in both clusters (orange bars). See Supplementary Methods, Supplementary Figs 10 and 11 and Supplementary Tables 8–11 for more detailed information. The complete mRNA expression data set is available at Gene Expression Omnibus, GEO accession number GSE40788. (**b**) The dendogram of the cell lines that make up the two clusters in the heatmap in **a**. The cell line names are coloured as follows; first column, OCI (blue) and SOC (red); second column, papillary serous (dark green) and other histotypes (orange); third column, papillary serous (dark green), clear cell (light blue), endometrioid (pink), mucinous (light green) and other histotypes (orange).

Unsupervised hierarchical clustering of the mRNA expression of 25 OCI and 6 SOC lines reveal two major clusters. There are 558 upregulated mRNAs in cluster 1 and 265 mRNAs in cluster 2 (Fig. 2; Supplementary Tables 8 and 9; Supplementary Data 2; see GSE40788 for the complete data set).

Interestingly, mRNA cluster 1 contains only OCI lines and most of the papillary serous lines (10/12) (Fig. 2). In contrast, cluster 2 predominantly contains the non-serous lines (10/12), and all of the SOC lines (6/6) (Fig. 2). Others have shown that mRNA profiles of primary human serous cancers constitute a distinct group, which is consistent with the above results^30^,^31^,^32^. However, it was also observed that the gene expression profiles of a subset of endometrioid and clear-cell cancers overlap with papillary serous tumours in these analyses^30^,^31^,^32^. Reminiscent of this pattern, we also observed that some of the endometrioid and clear-cell OCI lines are in cluster 1 with the serous cell lines (Fig. 2).

These results suggest that some of the tumours that are histologically classified as endometrioid or clear-cell cancers may have a papillary serous-like gene expression signature. In summary, mRNA cluster 1 generally correlates with papillary serous histology and OCI cell lines, and mRNA cluster 2 correlates with non-papillary serous histology and SOC cell lines. To exclude medium-based differences, both OCI and SOC lines were cultured in OCMI medium for these experiments.

In addition, SOC lines were cultured in standard versus OCMI media in a control experiment. We observed that SOC lines cluster next to each other regardless of whether they are cultured in OCMI or in standard media (Fig. 2b).

These results indicate that the mRNA expression differences observed between OCI versus SOC cell lines and between cluster 1 versus 2 are unlikely to be due to culture medium differences.

To uncover the pathways that are differentially expressed between cluster 1 versus -2 cell lines, we used Ingenuity Pathway Analysis (Supplementary Figs 10 and 11). This analysis revealed that 37 pathways are upregulated in cluster 1, including NF-kB, CXCR4, IGF-1, Rho-GDI, ILK, IL-6 and IL-8 signalling pathways (Supplementary Table 10). The 41 pathways that are upregulated in cluster 2 include Notch, BRCA, GADD45, Granzyme and Stathmin signalling pathways (Supplementary Table 11; see Supplementary Data 3 for the full list of pathways).

### Protein and mRNA profiles identify the same OCI clusters

**Figure 3:**
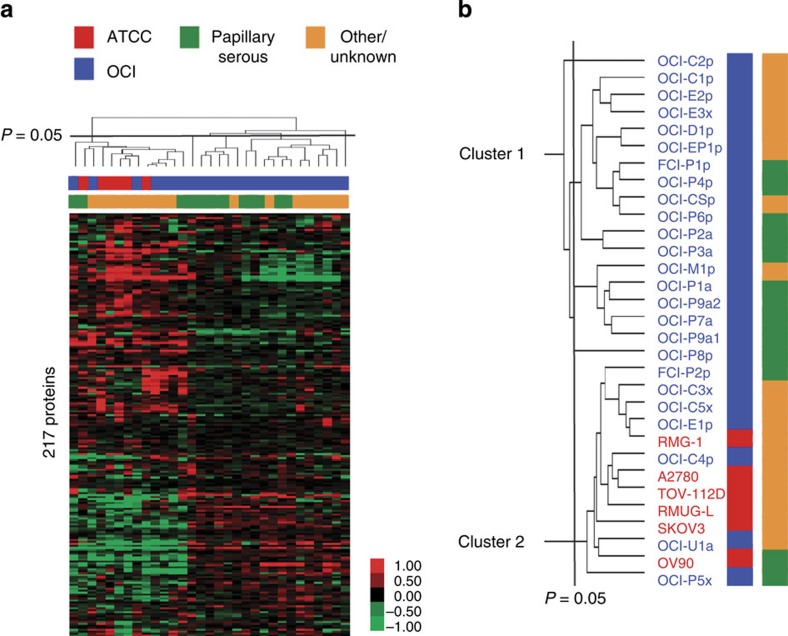
The proteomic profiling of OCI cell lines identifies two major clusters. (**a**) The unsupervised clustering of protein expression (measured by RPPA) in OCI cell lines (blue bars) together with SOC ovarian cancer cell lines (red bars) reveal two distinct clusters. Rows represent cell lines and columns represent antibody probes for each protein. The red and green colours reflect relative high and low expression levels, respectively. As in the mRNA clustering, cluster 1 contains only OCI cell lines (top half of the heatmap, blue only), and cluster 2 contains a mixture of SOC and OCI cell lines (bottom half of the heatmap, red and blue). While the papillary serous histotype almost exclusively aligns within cluster 1 (green bars), the non-papillary serous subtypes (orange bars) are divided between cluster 1 and cluster 2. (**b**) The dendogram of the cell lines that make up the two clusters in **a**. The cell line names are coloured as follows; papillary serous (green) and other histotypes (orange). See Supplementary Methods, Supplementary Figs 12–14, Supplementary Tables 12 and 13 and Supplementary Data 4 and 5 for more detailed information.

We next used reverse-phase protein array (RPPA) to examine the expression levels of 226 proteins in OCI and SOC cell lines. These proteins were selected because they represent major signalling pathways in cancer cells^33^. The unsupervised hierarchical clustering of the protein expression data reveal two major clusters that are nearly identical with the mRNA groups: the RPPA cluster 1 contains only OCI lines as well as most of the papillary serous lines (10/12) (Fig. 3). In contrast, RPPA cluster 2 predominantly contains the non-papillary serous lines (10/13), and all of the SOC lines (6/6) (Fig. 3). See Supplementary Data 4 and 5 for the full list of RPPA antibodies and proteins differentially expressed between clusters 1 versus 2.

It is worth noting that in the above experiments the mRNA and protein extracts were prepared in triplicates from three different passages in two separate experiments. Reassuringly, we observed that replicates from different experiments cluster together (Supplementary Fig. 12; Supplementary Table 12).

Moreover, we confirmed that OCI lines maintain their phenotype during long-term culture by comparing the RPPA profile of 10 pairs of cell lines that were analysed at two different times separated by 10–48 passages (Supplementary Fig. 13a). Unsupervised hierarchical clustering based on the expression of 218 proteins demonstrate that the three replicates and two different passages of each cell line cluster next to each other with one exception that, nevertheless, remained in the same cluster with a slight separation (Supplementary Fig. 13b,c). Furthermore, comparison of the list of cell lines in mRNA versus RPPA clusters reveal that they are identical with just one exception (Supplementary Table 13). These results indicate that the molecular differences between OCI versus SOC lines are stable and reproducible across many passages and analytical platforms.

### OCI cell line xenografts reproduce human tumour histology

To examine the *in vivo* tumour phenotype of the OCI lines, we injected each cell line into immunocompromised mouse hosts.

Ovarian carcinomas are characterized by very distinct morphological features^34^ (Supplementary Fig. 14). These differences in tumour morphology reflect relevant differences in gene expression and clinical features^31^,^32^. However, recapitulating architectural features of primary tumours has been an elusive goal in most xenograft tumour models.

**Figure 4:**
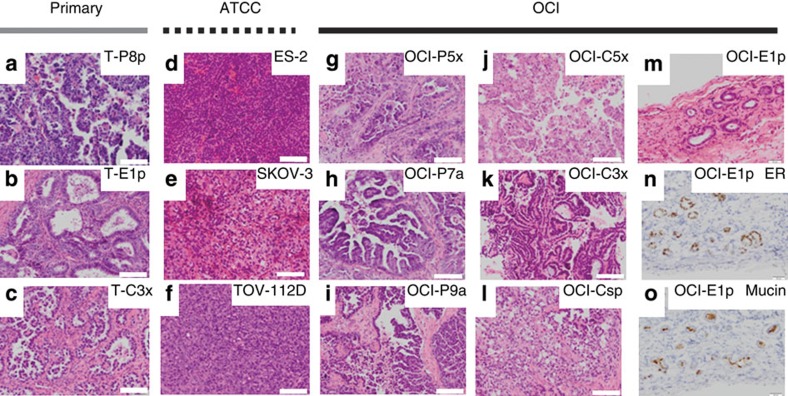
Histopathology of OCI xenografts recapitulate the original human tumour. (**a**–**c**) Haematoxylin and eosin (H&E)-stained sections of primary human tumours used to establish OCI-P8p (papillary serous), OCI-E1p (endometrioid) and OCI-C3x (clear cell) cell lines. (**d**–**f**) H&E-stained sections of xenografts tumours derived by injecting SOC cells (ES2, SKOV3 and TOV-112D) subcutaneously into immunocompromised mice. The typical features of human adenocarcinomas such as glands, papillae, stromal cores and desmoplastic stroma are absent. (**g**–**o**) H&E-stained sections of xenograft tumours derived by injecting OCI cell lines (P5x, P7a, P9a, C5x, C3x, CSp and E1p) subcutaneously into immunocopromised mice. In papillary serous specimens, note the presence of stromal cores and papillary architecture (**g**–**i**). In the endometrioid specimen note the presence of glands (**m**) which were positive for oestrogen receptor (ER) and mucin (brown), respectively, consistent with the endometrioid phenotype (**n**,**o**). Scale bar, 100 μM. See Supplementary Fig. 14 for additional images.

While it is difficult to entirely phenocopy a primary tumour in a xenograft, we hoped to recapitulate some of the cardinal features of different histotypes. For papillary serous carcinoma, these morphologic features would include finger-like structures (papillae) that consist of central stromal cores giving rise to smaller branches lined by a malignant epithelium with minimal cytoplasm and very large, high-grade, round nuclei^34^ (Fig. 4a). In contrast, endometrioid adenocarcinoma—named for its similarity to the endometrium—features back-to-back glands that are organized around central lumina surrounded by elongated malignant epithelial cells with abundant cytoplasm^34^ (Fig. 4b). Another distinct subtype, clear-cell carcinoma, typically forms microcysts, glands and/or papillae that are lined with cells with abundant cytoplasm that appears clear in haematoxylin and eosin stains due to excess cytoplasmic glycogen^34^ (Fig. 4c). Instead of glycogens, mucinous cancers have high levels of mucin in their cytoplasm^34^ (Supplementary Fig. 5i).

In general, SOC lines produce poorly differentiated xenograft tumours in mice, without any of the distinctive histopathologic features of specific ovarian tumour subtypes (Fig. 4d–f).

In contrast, the OCI lines produce tumour xenografts with a histopathology strongly resembling the original human tumour (Fig. 4g–o). OCI-P5x, P7a and P9a were established from human papillary serous carcinoma, and they recapitulate the papillary serous-like-specific architecture in immunocompromised mice (Fig. 4g–i). The OCI-C3x and C5x lines were established from human clear-cell tumours, and they form microcysts and papillae lined by clear cells in mice (Fig. 4j,k). The OCI-CSp line was established from a poorly differentiated carcinosarcoma and it forms a poorly differentiated tumour in mice (Fig. 4l). The OCI-E1p line was established from an endometrioid adenocarcinoma and forms oestrogen receptor-positive tumours with a glandular architecture, recapitulating the original tumour phenotype (Fig. 4m–o).

In summary, quite remarkably, the OCI lines form tumours that are morphologic phenocopies of corresponding human ovarian carcinomas at the histopathologic level, unlike SOC lines, which generally lack this characteristic.

### The OCI lines recapitulate histotype-specific features of tumours

Several molecular features help distinguish the histological subtypes of human ovarian carcinomas^35^. High-grade serous ovarian carcinomas (HGSOCs) are typically associated with high frequency of p53 mutations, as well as elevated WT1 and p16 protein expression in >80% of the cases^36^,^37^,^38^. The low-grade serous ovarian carcinomas are also WT1 positive; however, they tend to lack p53 mutations and express low levels of p16 (refs 36, 37, 38). In contrast, clear-cell ovarian carcinomas are associated with PI3K mutations, high HNF1B and low/absent ARID1a expression^36^,^37^,^38^,^39^, and they can be WT1 positive in ∼20% of the cases^40^.

**Figure 5:**
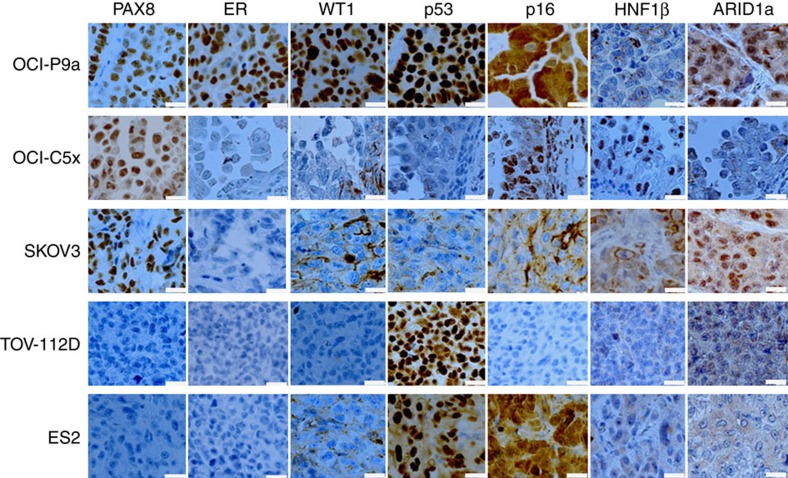
Histotype-specific immunostains of SOC and OCI xenograft tumours. Formalin-fixed paraffin-embedded sections of xenograft tumours derived from OCI and SOC lines were stained with ovarian carcinoma histotype-specific markers; PAX8, oestrogen receptor (ER), Wilm’s tumour (WT1), p53, p16, HNF1β and ARID1a. Nuclear staining (brown) for PAX8, ER, WT1, p53, HNF1β and ARID1a is specific. The occasional cytoplasmic or stromal staining with these markers is nonspecific. In contrast, tumour-specific p16 staining is typically cytoplasmic. Scale bar, 20 μM. See Supplementary Fig. 15 for additional images.

The *in vivo* histotype-specific molecular features described above are preserved to a great extent in OCI cell lines. On the basis of an algorithm described previously^36^,^37^,^38^, it is not difficult to place each OCI line into a specific ovarian cancer histotype category (Table 1; Fig. 5; Supplementary Figs 16 and 17). In contrast, SOC lines contain unusual features that are frequently inconsistent with *in vivo* tumour histotypes.

Two of the SOC cell lines we examined—SKOV3 and A2780—account for nearly 60% of ovarian carcinoma-related publications according to a recent report^13^, and just 5 SOC lines account for nearly 90% of published work^13^. While some of these SOC lines have histotype-specific mutations such as in p53, PIK3Ca and ARID1a (Table 1), their remaining profile is frequently at odds with this mutational state. For example, the SOC lines ES2, OV90 and TOV-112 do not express high levels of WT1 and p16, despite having p53 mutations (Table 1)^38^. Others have reported that only 5 of 32 SOC cell lines are WT1 positive, and of these only three have mutant p53 (ref. 38). Hence, it appears that <10% of SOC cell lines have HGSOC-like phenotype even though ∼70% of malignant epithelial human ovarian cancers are HGSOCs^13^.

The putative cells of origin of ovarian cancer include the normal ovarian surface, ovarian inclusion cysts and fallopian tube epithelia that are almost uniformly cytokeratin-7 (CK7) positive^18^,^41^. In addition, a majority of inclusion cysts and fallopian tube epithelium and a minority of ovarian surface epithelium are composed of CK7/PAX8 double-positive cells^18^. This is a distinctive profile that helps distinguish normal ovarian and fallopian tube epithelium from other tissues^42^,^43^. Consistent with this, almost 90% of epithelial ovarian cancers are keratin 7 and/or PAX8 positive^40^,^43^ and 70% oestrogen receptor (ER) positive^44^. Reassuringly, we found that the great majority OCI lines we examined (86%) are also positive for ER, PAX8 and/or CK7 as expected (Table 1).

In contrast, some SOC lines such as OV90 and TOOV-112 do not express any of the ovarian-specific markers (Table 1) and others express only one of three markers we examined (A2780 and ES2), raising some doubt about the differentiation state of these lines.

### mRNA profile of OCI lines correlates with patient outcomes

**Figure 6:**
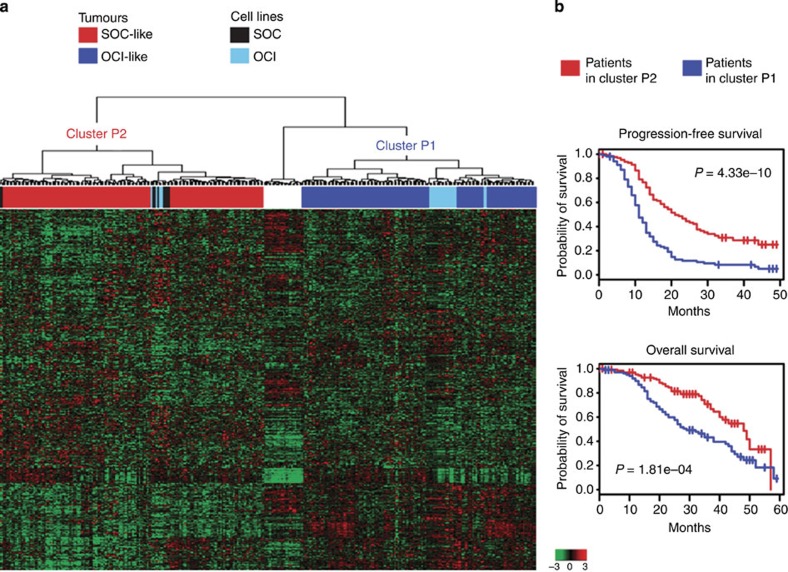
The OCI lines identify two major outcome groups. (**a**) Unsupervised hierarchical clustering of gene expression data of 37 cell lines and 285 human tissues reveal three clusters. Genes with ≥2-fold expression differences relative to the median value across cell lines and tissues in at least four samples were selected for hierarchical clustering analysis (3,831 gene features). Rows represent individual genes and columns represent each cell line or tissue. The red and green colour in cells reflect relative high and low expression levels, respectively, as indicated in the scale bar (log2-transformed scale). Red and blue bars above the heatmap indicate human tumour samples; light-blue bars indicate OCI lines; and black bars indicate SOC lines. While SOC cell lines (black bars) are exclusively group within patient cluster P2 (red bar), the OCI cells (light-blue bars) predominantly group within patient cluster P1 (blue bar). A small subset of tumour samples form a third cluster that does not include any cell lines (white); this group was excluded from outcome analysis. The complete mRNA expression data set is available at Gene Expression Omnibus, GEO accession number GSE40788 and GSE9899. (**b**) The progression-free and overall survival analysis data of patients with the ovarian tumours in patient cluster 1 and 2 in **a**. The patients with tumours that have a gene expression profile to OCI lines (blue bar, cluster 1 in **a**) have worse outcomes than patients with tumours that have gene expression profiles similar to SOC lines (red bar, cluster 2 in **a**). The small cluster of tumours that did not include any cell lines (white) was excluded from the outcome analysis.

Next, we compared the mRNA expression profile of the cell lines with primary human tumours. Clustering analysis of the OCI and SOC cell lines with 285 primary human ovarian tumour specimens reveal two distinct patient clusters. Patient cluster 1 that includes only OCI lines and patient cluster 2 that includes all of the SOC lines (Fig. 6a). Complete data set available at Gene Expression Omnibus (GEO) (GSE9899).

Significantly, the distribution of the cell lines within two human tumour clusters is identical to the *in vitro* cell line mRNA Clusters 1 and 2, with the exception of a single cell line (OCI-C4p) (Supplementary Table 12). This result indicates that the *in vitro* phenotype of the OCI cell lines generally conforms to *in vivo* clinical tumour phenotypes. Furthermore, the comparison of the clinical outcomes of these two groups reveal that the patients who have OCI-like tumours (patient cluster 1) have a worse progression-free and overall survival compared with patients with SOC-like tumours (patient cluster 2) (Fig. 5b).

### Drug response of OCI lines correlates with patient outcomes

The striking difference between the outcome of OCI-like versus SOC-like patient clusters prompted us to test whether the drug response of the corresponding cell lines are different as well. To test this, we treated OCI and SOC cell lines with taxol and cisplatin, which are the two most commonly used first-line drugs for the treatment of ovarian cancer.

In these experiments, we tested a subset of OCI and SOC lines from mRNA/RPPA cluster 1 and cluster 2 that are representative of a spectrum of different tumour subtypes (papillary serous, clear cell, carcinosarcoma, endometrioid and mucinous), and tissue sources (solid tumours, ascites fluid and xenograft explants) (Supplementary Table 2). Finally, to exclude medium-based differences, both OCI and SOC lines were cultured in OCMI medium for these experiments.

**Figure 7:**
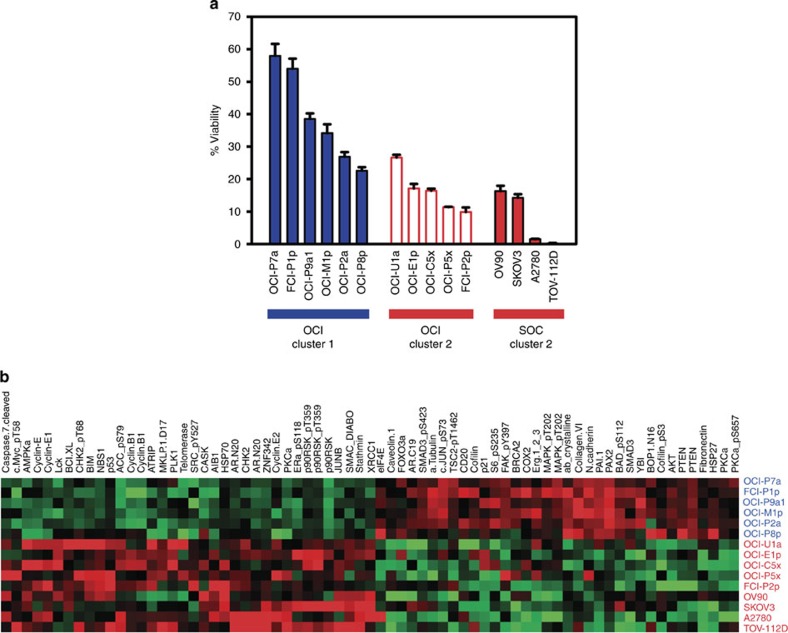
The OCI lines identify two major taxol and cisplatin response groups. (**a**) Taxol response of OCI and SOC cell lines that are in mRNA/RPPA cluster 1 versus cluster 2. The OCI and SOC lines were plated in triplicates in OCMI medium (3,000 cells per well) in 96-well plates. The next day, 20 nM taxol was added and metabolic activity was measured as 590/530 fluorescence via Alamar Blue after 96 h. OCI cell lines in mRNA/RPPA cluster 1, blue bars; SOC cell lines in cluster 1, red bars; OCI lines in cluster 2, white bars. The results are representative of four different experiments. See Supplementary Figs 18–20 for further details. (**b**) Proteins that are differentially expressed in mRNA/RPPA cluster 1. Analysis of RPPA data from OCI and SOC lines revealed a subset of proteins and phosphor-proteins that are differentially expressed in the taxol-resistant OCI lines (cluster 1, blue labels; cluster 2, red labels; *P*<0.05, Student’s *t*-test). Rows represent cell lines and columns represent antibody probes for each protein. The red and green colours reflect relative high and low expression levels, respectively. Please see Supplementary Tables 14 and 15 and Supplementary Data 6 for further details.

Consistent with the worse outcome of patients in the OCI-like cluster, we observe that the OCI lines in mRNA/RPPA cluster 1 are more resistant to taxol, cisplatin and vincristine compared with SOC/OCI lines in mRNA/RPPA cluster 2 (Fig. 7a; Supplementary Figs 19–21).

It is worth noting that there was at least 5–36 passages between the RPPA analysis and taxol/cisplatin-sensitivity experiments (Supplementary Table 13). Nevertheless, there was a close concordance between RPPA clusters and drug response, providing additional evidence that the phenotype of OCI lines is stable in culture. Moreover, these drug response experiments were also carried out with equal number of serous, clear-cell and endometrioid subtypes in each group (cluster 1 versus 2) (Supplementary Table 14), indicating that the difference in drug response is independent of histotype (Supplementary Fig. 20).

To explore the possible basis for the relative drug resistance of OCI cells, we compared the protein profiles of cluster 1/OCI lines with cluster 2/SOC lines (Fig. 7b), and found that drug-resistant OCI lines in cluster 1 overexpress several proteins that have been previously associated with taxol and cisplatin resistance including tubulin (target of taxol), PAX2, Cox2, PAI1, AKT, PTEN, SMAD3, caveolin and activated Erk (MAPKpT202) (Supplementary Tables 14 and 15)^45^,^46^,^47^,^48^,^49^,^50^,^51^.

The drug-sensitive SOC lines in cluster 2 express higher levels of several pro-apoptotic proteins, for example, Bim, SMAC-DIABLO, cleaved caspase 7 and lower levels of inactive phosphorylated BAD (pS112), a BH3-only pro-apoptotic protein. High Bim levels have been inversely correlated with activated Erk (MAPKpT202), which phosphorylates Bim and targets it for ubiquitination and degradation^52^,^53^,^54^,^55^. Hence, these differences could render cluster 2 cells more sensitive to chemotherapy-induced apoptosis. (Supplementary Tables 14 and 15). See Supplementary Data 7 for the full list of proteins.

We also examined the correlation of the OCI cell lines with molecular subtypes of ovarian cancer. Two large studies (*n*=734) in the US (TCGA) and Australia (AOCS) classified ovarian cancers based on their molecular profiles^56^,^57^. In the TCGA study, four subgroups were identified: mesenchymal, differentiated, immunoreactive and proliferative^57^. In the AOCS study, six prognostic subgroups were identified (c1–c6)^56^, with the additional subtypes being comprised of tumour histotypes not included in the TCGA study that was restricted to high-grade serous ovarian cancer. It was found that the TCGA mesenchymal subtype and AOCS C1 subtypes displayed a significant trend towards early relapse and short overall survival^56^,^57^.

**Figure 8:**
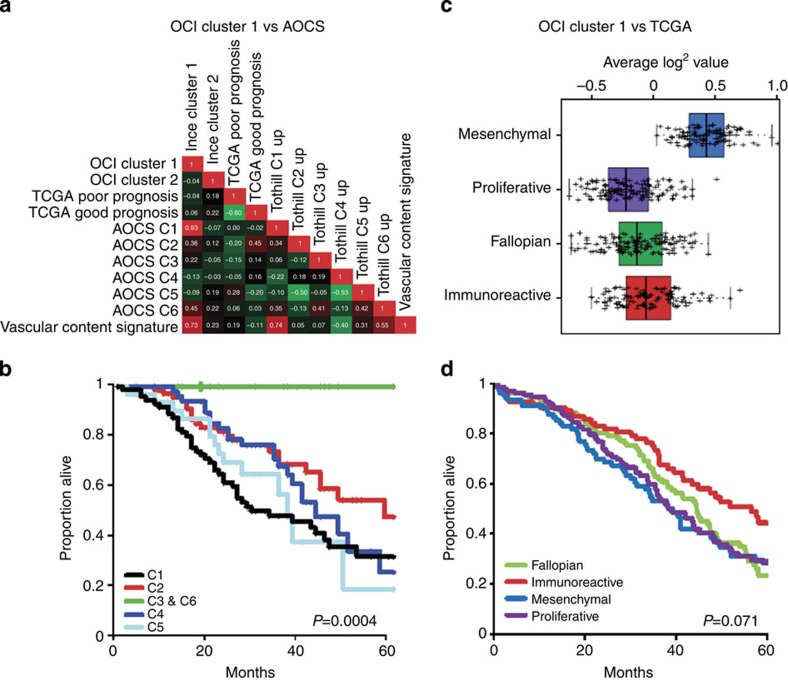
The OCI cluster 1 is similar to TCGA and AOCS poor-outcome groups. (**a**) Pearson correlation values for each of the signature scores indicate a very strong correlation among the genes that are expressed in the OCI cluster 1 and the Tothill C1 group (*R*=0.93). Interestingly, both of these signatures also exhibit a strong correlation with genes that are abundantly expressed in vascular endothelial cells (>0.7)^60^, suggesting that both of these signatures define ovarian cancer cells with mesenchymal/endothelial attributes. (**b**) The Kaplan–Meier overall survival plot of the Tothill C1–C6 groups indicate that the Tothill C1 group that correlated with OCI cluster 1, has one of the poorest outcomes. (**c**) The box-and-whisker plots of the signature scores for the TCGA ‘poor prognosis genes’ demonstrate that the TCGA mesenchymal group is most similar to OCI cluster 1 in a statistically significant manner. The two-tailed *t*-test was used to compare the mesenchymal group signature score versus the three other groups (*P*=0.034). The interquartile range is shown by the box and the bar within the box represents the median value. (**d**) The Kaplan–Meier overall survival plot of the 482 TCGA tumours with clinical follow-up demonstrate that the TCGA mesencyhmal subtype that correlates with the OCI cluster 1 also has one of the worst outcomes. See Supplementary Figs 21 and 22 for further details.

Interestingly, we found that our OCI cluster 1-specific signature that correlates with poor clinical outcome was nearly identical to the AOCS C1 (*R*=0.93) (Fig. 8a,b; Supplementary Fig. 22) and was most similar to the TCGA mesenchymal subtype (Fig. 8c,d). These correlations provide support for prognostic models that validated these signatures^58^,^59^,^60^. To explore the possibility that the OCI lines differ in their epithelial/mesenchymal state, we also compared the OCI mRNA levels with epithelial–mesenchymal transition core signature profiles^61^. We found that cluster 2 OCI samples are characterized by high levels of epithelial markers and cluster 1 OCI lines high levels of mesenchymal markers consistent with the above results (Supplementary Fig. 22).

## Discussion

Over the past six decades, the great majority of human cancer subtypes have proven to be difficult to propagate in cell culture. This problem derives from the suboptimal conditions of *in vitro* culture and culture media that poorly recapitulates the conditions in living tissue. The resulting panels of cancer cell lines have hampered many attempts at preclinical drug development, in that the behaviour of the limited set of available cell lines, both *in vitro* and *in vivo*, are frequently unpredictive of clinical responses.

Here, we present a method for propagating a diverse array of ovarian carcinoma cell types *in vitro*. Use of this method, in the form of a novel medium and alterations in oxygen and serum levels, has yielded to date a panel of 25 new ovarian cancer cell lines that are extensively characterized, with histopathological and molecular analyses that includes whole-genome profiles of DNA and RNA data and protein arrays. In this report, we provide detailed description of the medium and methods in the Supplementary Material. In addition, the cell lines described here are made available to the scientific community as a renewable resource for basic and translational research. These new cell lines will nearly double the number of ovarian carcinoma cell lines available from public repositories.

The molecular profile of OCI cell lines we describe here demonstrate a remarkable stability over time and consistency across multiple platforms including their DNA, mRNA and protein profiles^62^, and recapitulate clinically relevant patient populations. For example, there was a remarkable similarity between mRNA expression signature of poor outcome patient tumours in the AOCS C1 and TCGA mesenchymal data sets and our poor outcome cell lines (OCI cluster 1). In addition, we found that OCI cluster 1 lines have an epithelial–mesenchymal transition-like signature that has been associated with cancer stem cells, resistance to chemotherapeutic treatments and invasion of tumour cell clusters through the mesothelial lining of the peritoneal cavity—a critical step in the dissemination of ovarian carcinoma^62^. These observations lend support for the potential relevance and utility of OCI cell lines for ovarian cancer research.

Consistent with these results, we found that OCI lines in cluster 1 lines are more resistant to taxol and cisplatin, both first-line treatments for ovarian cancer, compared with SOC/OCI cell lines in cluster 2. This result provides the beginning of evidence that the *in vitro* drug responses of these OCI lines may indeed correlate closely with *in vivo* patient responses to drug treatments. Such a correlation between *in vitro* cell line data and *in vivo* patient data is especially encouraging since it has been recognized that such correlations are rare with standard tumour cell lines, which tend to be more drug sensitive than human tumours, leading to false-positive hits in cell culture-based drug screens. The closer correlation between OCI lines and human ovarian tumours is perhaps not surprising, since we observed that cytological, morphological and molecular features of the OCI lines and their xenograft tumours resembled specific subtypes of human ovarian cancer, which has not been the case for most SOC lines.

While the overall correlation between cell line taxol/cisplatin response and patient outcome is encouraging, long-term prospective follow-up studies will be required to determine whether OCI lines will be predictive of patient response.

Currently, the number of cells in our OCI panel is too small to carry out robust statistical analysis to determine whether there are >2 significant clusters. It is possible that with a larger panel of cell lines we will discover additional molecular subtypes. Furthermore, more diverse data sets from human tumours will be needed for histotype-specific studies, because over 95% of the nearly 1,500 samples in 12 publically available gene expression data sets with outcome data are from stage 3–4, high-grade (3) serous ovarian cancers^58^,^63^. The data set that includes non-serous histotypes contains 8 clear-cell, 13 mucinous and 37 endometrioid cancers; however, no outcome data are available for these samples^18^,^63^. There is a close correlation between form and function in biology, and the marked differences in tumour architecture and cell morphology among distinct ovarian cancer histotypes suggest deeper underlying molecular differences that are yet to be discovered^38^.

Regarding mRNA expression subtypes of ovarian cancer, it is worth pointing out that several of the AOCS/TCGA subcategories are characterized by immune (c2), stromal (c4) or proliferation (c5) signatures. As we described, the immune and stromal cells have to be eliminated from the culture for successful establishment of tumours as cell lines. Otherwise fibroblasts will typically outgrow the tumour cells. In OCMI medium, the non-tumour cells are naturally eliminated during the first several passages. Thus, the OCI cell lines are free of stromal and other cells, and as a result we did not expect them to replicate tumour subtypes that are defined by non-tumour signatures such as the c2 and c4 subtypes. While the *in vivo* proliferation rates of tumours can be markedly different, such as the c5 subtype, the successful culture of tumours involves expansion of proliferating cells. Hence, the *in vitro* proliferation rate differences are likely to be less pronounced compared with *in vivo* differences.

In conclusion, we describe here the initial instalment of our efforts as a proof of concept towards establishing a comprehensive ovarian carcinoma cell line panel. Even at this early stage the results are encouraging, and we are in the process of establishing additional lines representing each subtype that will be needed to explore the histotype-specific differences and intra-tumoral heterogeneity of ovarian tumours.

While we have successfully cultured a number of colon and lung cancer cells in OCMI medium, we do not recommend this medium as a universal culture environment for all tumours at this time. Our experience with other tumour types indicates that to achieve high success rates and maintain the phenotypic properties of the original tissue, each tumour type will require tissue-specific modifications in medium formulation for optimum results.

In the future, a robust and efficient culture system yielding cancer cell populations that predict patient responses to various drugs should improve development of new drugs for personalized treatment of cancer patients. It should also improve the ability to identify mechanisms of resistance and to develop rational drug combinations. Our preliminary results suggest that the methodology we describe here can be modified with tissue-specific changes to culture other tumour types such as leukaemias, as well as normal breast, ovarian and fallopian tube cells^16^,^18^,^64^. We foresee that this approach can one day be used for personalized oncology where the *in vitro* drug-sensitivity profile of each patient’s tumour can be assessed real time and this information can be used to guide treatment decisions.

## Methods

### Primary tumour culture and cell lines

The OCI cell lines and OCMI medium are available from the Live Tumor Culture Core at Sylvester Comprehensive Cancer Center, Miller School of Medicine, University of Miami http://sylvester.org/shared-resources/Live-Tumor-Culture-Core. To establish the cell lines, fresh tumour tissue fragments are minced and plated on Primaria (BD Biosciences) plates before and after digestion with 1 mg ml^−1^ collagenase (Roche). The tumour cells are cultured in nutrient medium described previously^16^, supplemented with insulin, hydrocortisone, epidermal growth factor (EGF), cholera toxin and serum as described in Supplementary Methods. We refer to this version of the medium as OCMI. This formulation is supplemented with 17β-estradiol for endometrioid and mucinous tumours (OCMIe). The papillary serous, clear-cell, dysgerminoma and carcinosarcoma tumours are initially cultured in 5% CO_2_ and regular O_2_ at 37 °C as monolayers attached to Primaria culture plates. The endometrioid and mucinous tumours are initially cultured in 5% CO_2_ and low O_2_ (5%) at 37 °C as monolayers attached to Primaria culture plates. Adjustments in O_2_ and plates are made for individual cell lines as necessary (Supplementary Table 3). The tumour cells are passaged at a ∼1:3 ratio once a week and plated into a new flask at ∼1 × 10^4^ cells cm^−2^. During the initial weeks of culture, (∼1–5) the plates are treated with diluted trypsin first, to deplete stromal cells. The remaining cells that are still attached to the culture plate are treated with 0.25% trypsin for subculturing. In general, tumour cultures are free of stromal and normal cell types within 4–6 passages. The SOC cell lines were short tandem repeat validated and cultured as per the instructions of the vendor. OCI lines will be available from the Ince laboratory upon publication. All study procedures were approved by the Institutional Review Boards of the University of Miami, Brigham & Women’s and Massachusetts General Hospitals to collect discarded tissues with written consent from all patients. See Supplementary Note 1 for further details of culture methods.

### Macromolecular analysis

Protein expression was analysed by RPPA, as described previously^65^,^66^. Replicate data were averaged, log2 transformed, median centred and subjected to hierarchical clustering using the un-centred Pearson correlation in Cluster (v.3.0) and Java TreeView (v.1.1.1). mRNA expression for the cell lines was measured using the Illumina HumanHT-12 v4 Expression BeadChip platform^67^. The gene expression data for 285 ovarian tumour samples were obtained from the GEO (accession number: GSE9899) and normalized by the robust multichip average (RMA) method^68^. The genomic DNA from tumours and cell lines were analysed with Affymetrix 250K Sty chips^69^. The copy-number analysis was performed using the Molecular Inversion Probe (MIP) 330 k microarrays from Affymetrix. The details of MIP assay have been previously described^70^. (See Supplementary Methods for further details.)

### Drug-sensitivity experiments

The relative sensitivities of OCI and SOC cell lines to chemotherapy drugs was measured by seeding an equal number of cells in six replicates in 96-well black-walled clear-bottom Corning plates at 3,000 cells per well, and allowing attachment in OCMI for 12 h. Both OCI and SOC cell lines were exposed to drugs in OCMI medium. The cell lines were cultured in the presence of drug or vehicle control for 96 h. The fraction of metabolically active cells after drug treatment was measured by incubation with 2:10 (v/v) CellTiter-Blue reagent (Promega Cat# G8081) in media for 2 h, and the reaction was stopped by addition of 3% SDS. Fluorescence was measured in a SpectraMax M5 plate reader (Molecular Devices, CA) using SoftMax software (555EX/585EM). See Supplementary Methods for additional details.

### Analysis of tumorigenicity

Single-cell suspensions were prepared in a Matrigel: medium mixture (1:1) and 1–5 million cells per 100 μl volume were injected in one intraperitoneal and two subcutaneous sites per mouse. Tumour cell injections were performed on 6–8-week-old female immunodeficient nude (Nu/Nu) mice (Charles River Laboratories International, Inc, Wilmington, MA). Tumours were harvested 5–9 weeks after implantation. Tumour histopathology was assessed with haematoxylin and eosin staining of formalin-fixed paraffin-embedded sections. All mouse studies were approved by the University of Miami, the BWH or MGH Institutional Animal Care and Use Committee.

### Mutational analysis

Mutation detection was carried out at the MD Anderson Cancer Center Sequencing and Microarray Facility by amplifying purified DNA with primers designed to amplify coding regions of the p53 gene (exons 5–9). Primers were designed using a variety of software applications including, but not limited to, Primer Express v3.0 (Applied Biosystems). The PCR products were purified with ExoSAP-IT (USB) and sequenced in both directions using BigDye Terminator chemistry (Applied Biosystems) and run on a 3730 DNA Analyzer (Applied Biosystems). The sequence data files were aligned and compared with a reference sequence (from Ensembl TP53: Gene ID ENSG0000014150 Transcript TP_002 ENST00000445888) in SeqScape v2.5 Software (Applied Biosystems) and mutations analysed.

### Gene and protein expression analysis

Analysis of mRNA and protein expression profiling data, LOH and DNA copy-number changes are described in the Supplementary Methods.

## Additional information

**Accession codes:** the mRNA (GSE40785), MIP (GSE40786) and SNP (GSE40787) data are available in Gene Expression Omnibus under accession code for the study GSE40788.

**How to cite this article:** Ince, T. A. *et al.* Characterization of twenty-five ovarian tumour cell lines that phenocopy primary tumours. *Nat. Commun.* 6:7419 doi: 10.1038/ncomms8419 (2015).

## Supplementary Material

Supplementary Information Supplementary Figures 1-22, Supplementary Tables 1-16, Supplementary Note 1, Supplementary Methods and Supplementary References

Supplementary Data 1 DNA from 12 primary uncultured tumors, 12 matched OCI cell lines, and nine SOC lines are analyzed using 250K Sty Affymetrix SNP arrays. (related to Supplemental Fig 7-8)

Supplementary Data 2 Unsupervised hierarchical clustering of mRNA expression levels of OCI and SOC ovarian cancer cell lines reveal two clusters. (Related to Fig 2)

Supplementary Data 3 Ingenuity Pathway Analysis (IPA) of the 823 genes that are differentially expressed between Cluster 1 vs. in Cluster 2 (p < 0.05) (Figure 3). The 558 transcripts up-regulated in Cluster-1 are associated with 37 core pathways in IPA (p < 0.05). The 265 transcripts up-regulated in Cluster-2 are associated with 37 core pathways in IPA (p < 0.05). (Related to Supplementary Fig 10-11)

Supplementary Data 4 The unsupervised clustering of protein expression (measured by RPPA) in OCI cell lines together with SOC ovarian cancer cell lines reveal two distinct clusters. (Related to Fig 3)

Supplementary Data 5 List of Antibodies used for RPPA analysis(Related to Fig 3)

Supplementary Data 6 The heatmap profiles derived from unsupervised clustering of data from RPPA analysis of OCI cell lines. Each column depicts a different antibody and each row depicts an individual replicate from each cell line(Related to Sup Fig 12)

Supplementary Data 7 Analysis of RPPA data from OCI and SOC lines reveal a subset of proteins and phosphor-proteins that are differentially-expressed in the Taxol resistant OCI lines in cluster 1 vs. SOC lines in cluster 2 (Related to Fig 7b)

## Figures and Tables

**Table 1:**
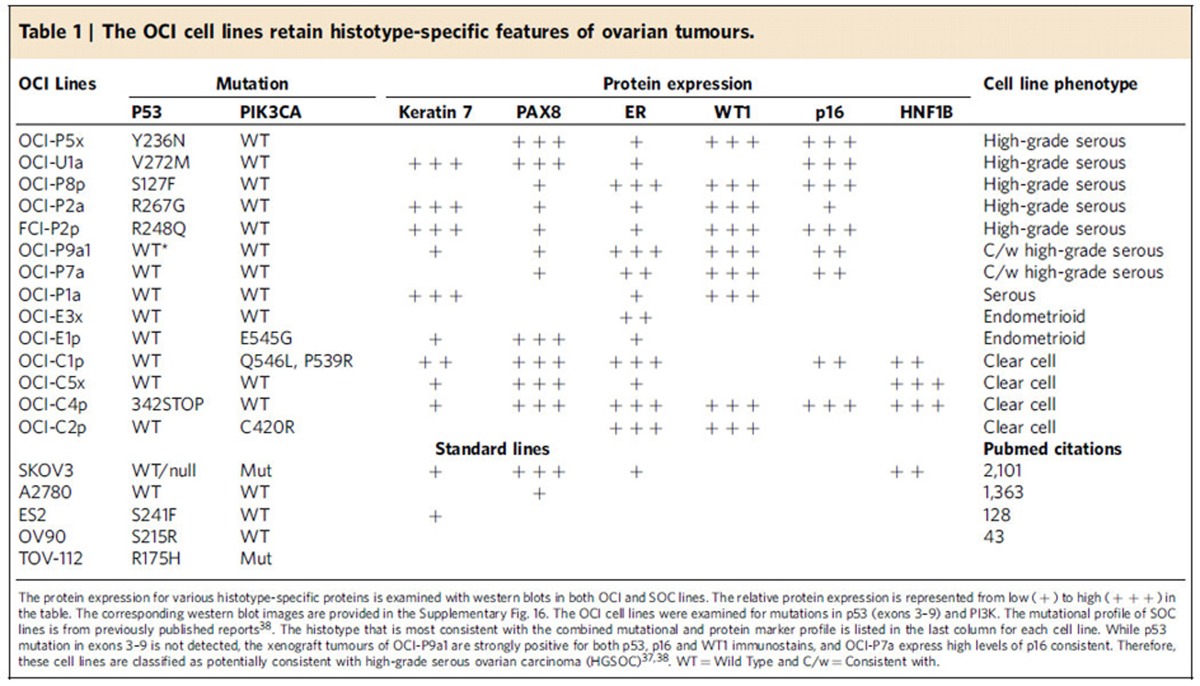
The OCI cell lines retain histotype-specific features of ovarian tumours.
