# Supplementary material for: Characterization of twenty-five ovarian tumour cell lines that phenocopy primary tumours
Source: Nat Commun. 2015 Jun 17;6:7419. doi: 10.1038/ncomms8419 (PMC4473807; doi:10.1038/ncomms8419)
Supplement: Supplementary Information — Supplementary Figures 1-22, Supplementary Tables 1-16, Supplementary Note 1, Supplementary Methods and Supplementary References [file ncomms8419-s1.pdf]

SUPPLEMENTARY INFORMATION

Supplementary Figure 1

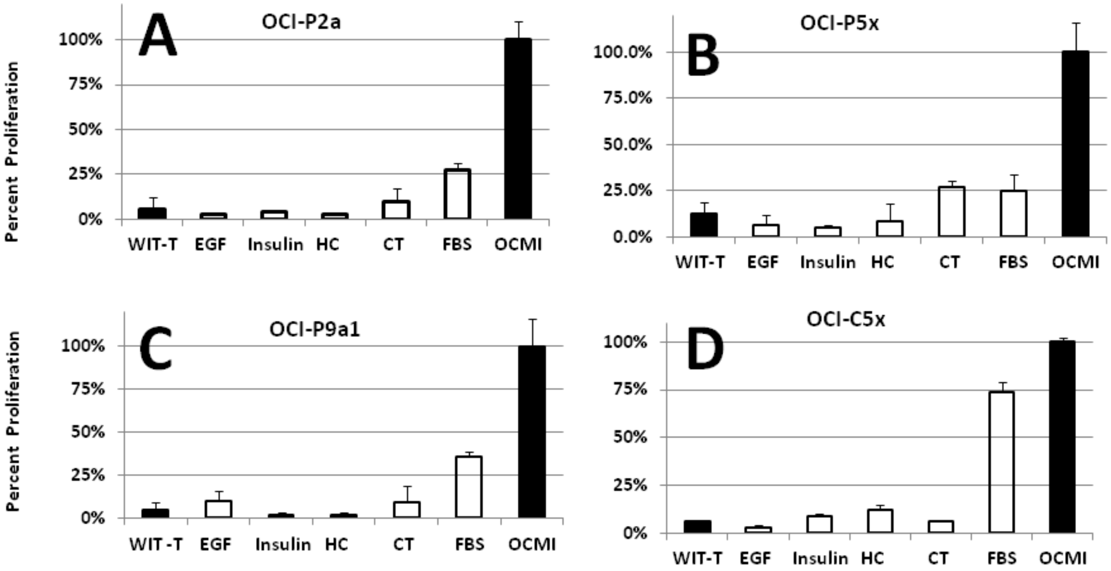

E

Component	WIT-T	+ EGF	+ Insulin	+ HC	+ CT	+ Serum	OCMI
EGF (ng/mL)	0.5	10	-	-	-	-	10
Insulin (ug/mL)	10	-	20	-	-	-	20
HC (ng/mL)	0.5	-	-	500	-	-	500
CT (ng/mL)	0	-	-	-	25	-	25
Serum (%)	0	-	-	-	-	5%	5

Combinatorial effects of OCMI components on Serous and Clear Cell tumors:

A-D) OCI cell lines established from papillary serous (OCI-P2a, OCI-P9a1, OCI-P5x) and clear cell carcinomas (OCI-C5x) were cultured in WIT-T base medium (control) supplemented with individual components; EGF, Insulin, Hydrocortisone (HC), Cholera Toxin (CT), Fetal Bovine Serum (FBS), or with combination of all these components (OCMI). In almost all cases the individual components have little effect on cell proliferation when added alone. However, when all variables are combined there is a 4-10 fold increase in cell proliferation. Cell proliferation is calculated as percent fraction of growth in the complete medium (OCMI). Adding serum alone to WIT-T base medium does not induce proliferation of tumor cells in three out of four tumor samples tested. In one cell line (C5x) FBS alone appear to stimulate cell proliferation up to 75 % of OCMI, however, this is a short-term effect. Furthermore, when used alone serum promotes over-proliferation of fibroblasts which dilutes the tumor cells. In each experiment 50,000 cells/well are plated in triplicates in six well plates and counted after five days. Error bars represent standard deviation of the mean of triplicates. Each experiment was repeated at least twice.

E) The final concentration of each medium component is listed in the table.

Supplementary Figure 2

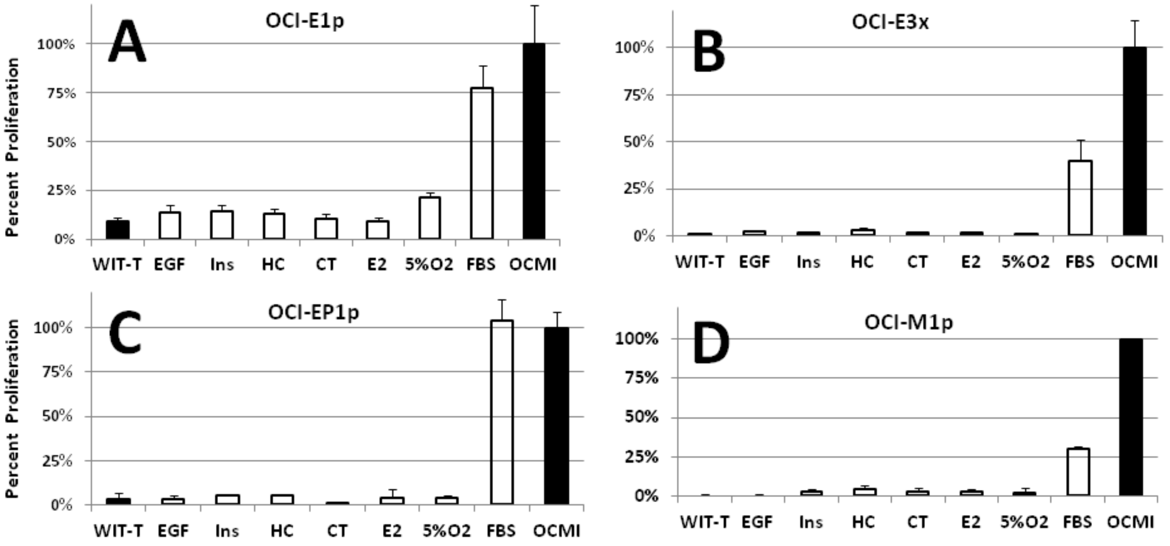

E

Component	WIT-T	+ EGF	+ Insulin	+ HC	+ CT	+ Serum	+E2	% O_2	OCMI
EGF (ng/mL)	0.5	10	-	-	-	-	-	-	10
Insulin (ug/mL)	10	-	20	-	-	-	-	-	20
HC (ng/mL)	0.5	-	-	500	-	-	-	-	500
CT (ng/mL)	0	-	-	-	25	-	-	-	25
17 β-Estradiol (nM)	1	-	-	-	-	-	100	-	100
Serum (%)	0	-	-	-	-	5%	-	-	5
% O_2	21	21	21	21	21	21	21	5	5

Combinatorial effects of OCMI components on Endometrioid and Mucinous Tumors

A-D) OCI cell lines established from endometrioid (OCI-E1p, OCI-E3x, OCI-EP1p) and mucinous carcinomas (OCI-M1p) were cultured in WIT-T base medium (control) supplemented with individual components; 17-β-Estradiol (E2), EGF, Insulin, Hydrocortisone (HC), Cholera Toxin (CT), Fetal Bovine Serum (FBS), or with combination of all these components (OCMIe) in regular oxygen levels (air) or in low oxygen (5%). In almost all cases the individual components had little effect on cell proliferation when added alone. However, when all variables were combined there was a significant increase in cell proliferation. Cell proliferation was calculated as percent fraction of growth in the complete medium (OCMI). While FBS alone appeared to stimulate cell proliferation to the level of OCMI in one of the four cell lines tested (EP1p), this was a short term effect that was not maintained in long-term culture. In each experiment 50,000 cells/well were plated in triplicates in six well plates and counted after five days. Error bars represent standard deviation of the mean of three replicates. Each experiment was repeated at least twice.

E) The final concentration of each component is listed in the table.

Supplementary Figure 3

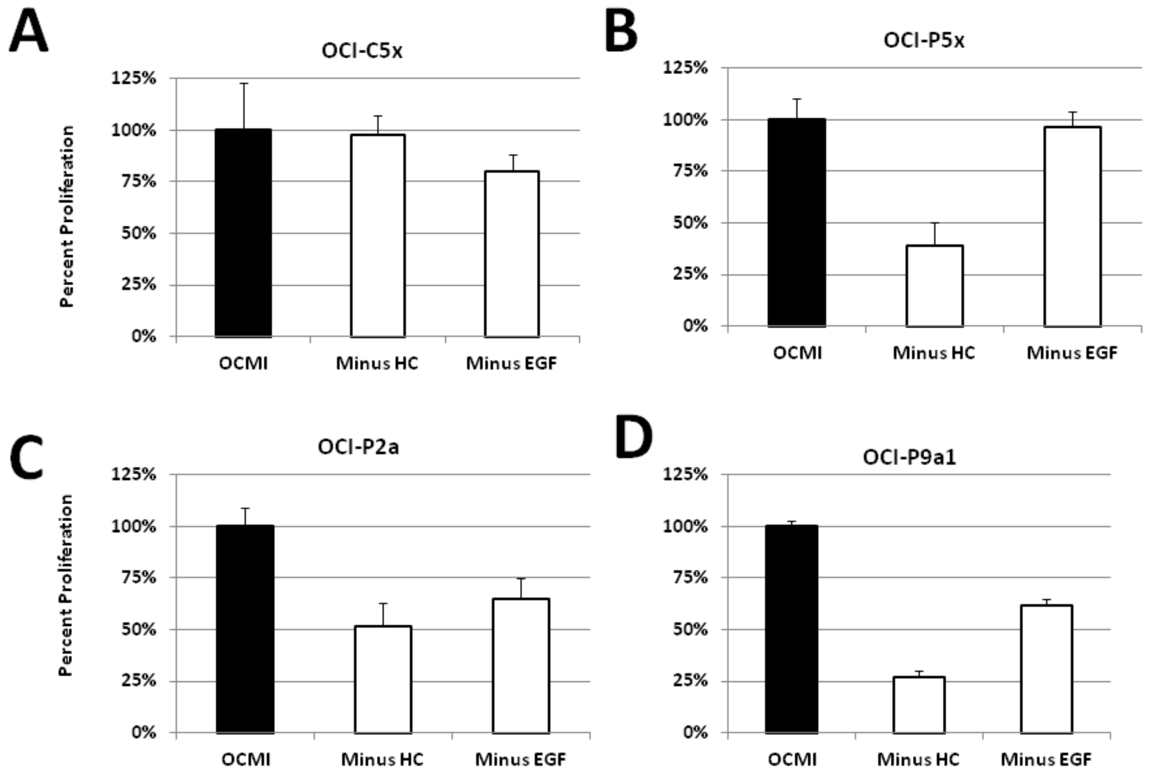

The effect of individual OCMI components varies in different OCI lines

Four different OCI lines (C5x, P5x, P2a, and Ppa1) were cultured in OCMI medium without hydrocortisone (HC) or without EGF supplement. While subtracting hydrocortisone had no effect on OCI-C5x **(A)**, it decreased proliferation of the other three cell lines. In contrast, while subtracting EGF had no effect on OCI-P5x **(B)**; it decreased proliferation of the other three lines. In each experiment 50,000 cells/well were plated in triplicates in six well plates and counted after five days. Error bars represent standard deviation of the mean of three replicates. Each experiment was repeated at least twice

Supplementary Figure 4

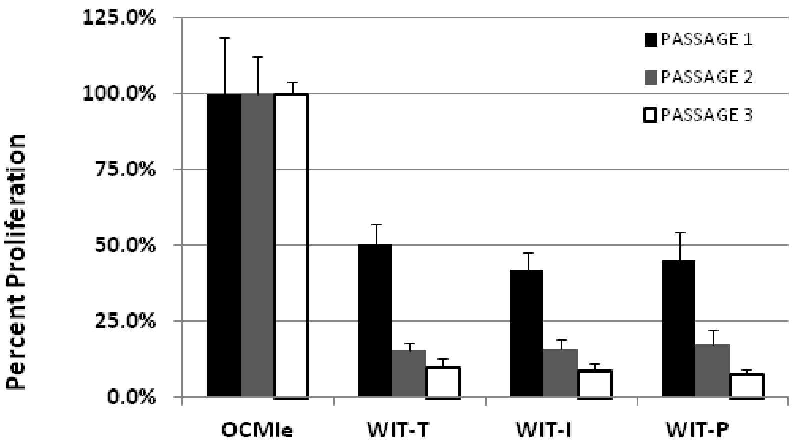

The effects of culture conditions become apparent after successive passages

The endometrioid OCI-E1p cell line was cultured three passages in OCMIe vs. WIT-T base medium, supplemented with 25 ng/mL cholera toxin (WIT-T) or with EGF (10ng/mL), Insulin (10ng/mL), Hydrocortisone (0.5 ug/mL), Cholera Toxin (25 ng/mL) (WIT-P). At each passage the cells were trypsinized, counted and plated once a week. During the first passage OCI-E1p cells proliferated in WIT-T, -I and P medium at ~50% of OCMIe. However, by passage three, proliferation in these medium dropped to less than 10% of OCMIe. In each experiment 50,000 cells/well were plated in triplicates in six well plates and counted after five days. Error bars represent standard deviation of the mean of three replicates. Each experiment was repeated at least twice

Supplementary Figure 5

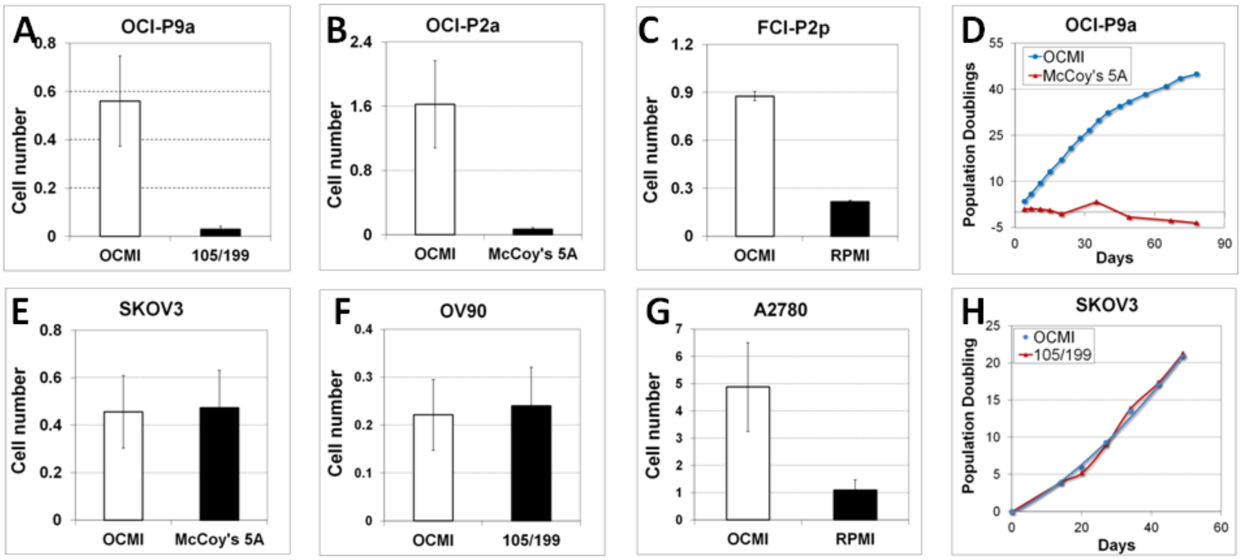

A-C) OCI ovarian cancer cell lines cannot be cultured in standard culture media

Equal numbers of cells from OCI-P2a, OCI-P9a and FCI-P2p cell lines were plated in OCMI medium in six well plates in triplicate (white bars) vs. standard cell culture medium MCDB105/M199, McCoy's 5A, or RPMI-1640 media supplemented with 15% serum (black bars). The cells were counted after 5 days in culture. The OCI cell lines immediately exhibited growth arrest in standard media. Error bars represent standard deviation of three replicates.

D) OCI lines do not adapt to standard media even after long term culture

Equal numbers of cells from OCI-P9a were plated in OCMI medium (blue line) vs. McCoy's 5A supplemented with 15% serum (red line) and cultured continuously for approximately 80 days. The cells were counted approximately once a week. While some cells in the OCI-P9a culture survived in McCoy’s 5A briefly, the cell line could not be expanded in this medium.

E-G) Standard ovarian cancer cell lines can be cultured in OCMI medium

Equal numbers of cells from SOC ovarian cancer cell lines (SKOV3, OV90, and A2780) were plated in OCMI medium (white bars) vs. McCoy's 5A, MCDB105/M199, or RPMI-1640 media supplemented with 15% serum (black bars). The cells were counted after 5 days in culture. All the SOC ovarian cancer cell lines proliferated in OCMI medium as well as recommended medium. A2780 proliferated faster and exhibited improved cell attachment in OCMI compared to RPMI. Error bars represent standard deviation of three replicates.

H) Standard ovarian cancer cell lines can be cultured long term in OCMI medium

Equal numbers of SKOV3 cells were plated either in OCMI medium (blue line) or in MCDB105/m199 supplemented with 15% serum (red line) and cultured continuously for 50 days. The cells were passaged and counted once a week. SKOV3 proliferated equally well in OCMI and in MCDB105/m199 over multiple passages.

Supplementary Figure 6

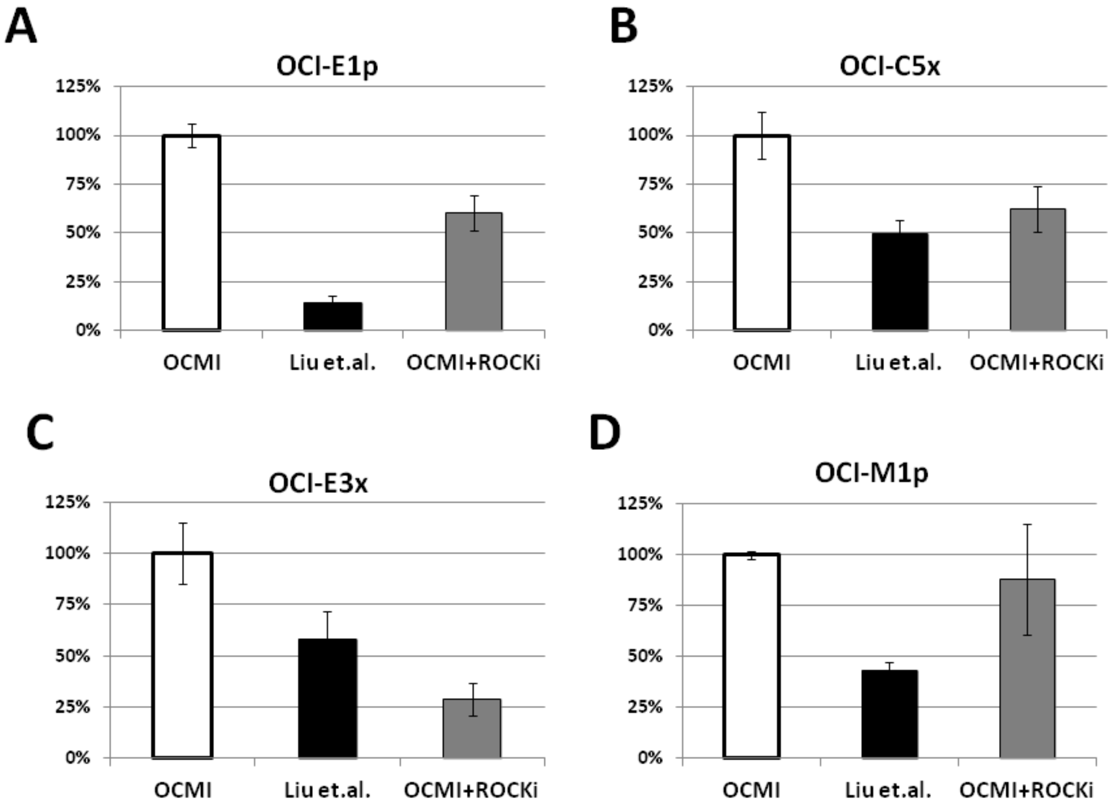

A-D) Proliferation of OCI lines is inhibited by ROCK inhibitor

We tested whether OCI cell lines can be cultured in the medium recently described by Watanabe *et al.*[1], who used a DMEM:F12 based medium with an apoptosis inhibitor (Y-27632) that inactivates Rho-associated kinase (ROCK) to culture human ES cells. More recently, Liu *et al.,* have used this approach to culture human tumors[2]. However, they described establishing only one normal ovarian cell line, and no ovarian cancer cell lines [2]. We cultured OCI lines in OCMI (white bars) vs. the medium described by Liu et al,. (black bars), or in OCMI with 10 mol/L ROCK inhibitor (Y-27632) (gray bars) [2]. The error bars represent standard deviation of the mean for three replicates. The results after three passages are shown. These results demonstrate that proliferation of OCI cells is inhibited in the medium described by Liu et al. Furthermore, Y-27632 inhibits proliferation of OCI lines even in OCMI medium (gray bars). Interestingly, it was recently shown that the ROCK Inhibitor Y-27632 also negatively affects the survival of CD34+ hematopoietic progenitor cells, human adipose-derived stem cells and proliferation of melanomas [3-5] suggesting that Y-27632 may have a differential impact on different lineages and tissue-specific stem cells.

Supplementary Figure 7

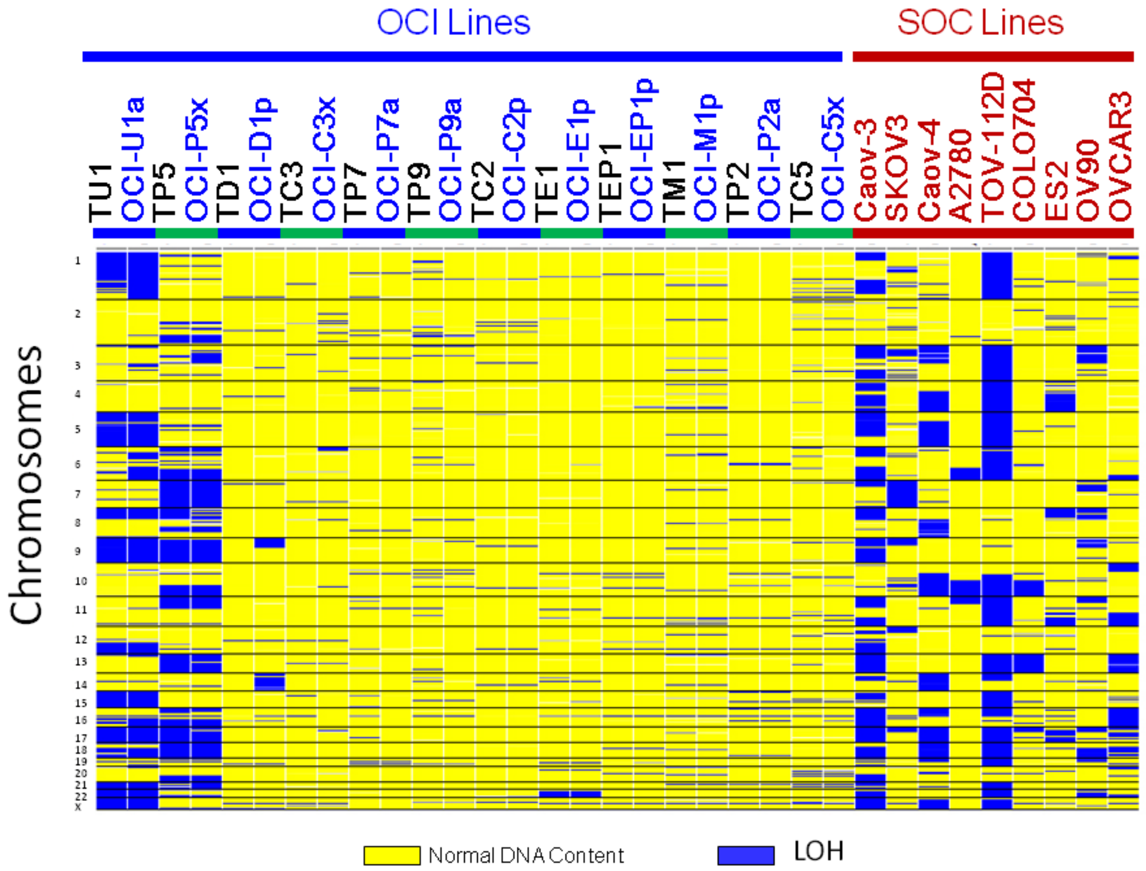

Comparison of OCI lines vs. original tumor tissue with LOH analysis

DNA from 12 primary uncultured tumors (T, black labels), 12 matched OCI cell lines (OCI, blue labels), and nine SOC lines (red labels) were analyzed using 250K Sty Affymetrix SNP arrays. Tumor-only LOH was determined by hidden Markov modeling with a standard rate of heterozygosity (the ratio of AB calls/total number of calls) of 0.2. Blue regions indicate inferred loss of heterozygosity, yellow indicates normal rate of heterozygosity. Each OCI cell line is placed beside its corresponding original tumor DNA sample for comparison. While OCI-U1p and OCI-P3a have large segments of LOH, this appears to be a feature of the original tumors they were derived from and not a cell culture artifact. The other 10 OCI lines contain LOH involving small segments of the chromosome arms. In contrast, all of the SOC cell lines tested contain large segments of LOH that involved whole chromosomes or chromosome arms**.** See online Excel data file 1 for LOH segment and breakpoint analysis. The complete dataset is available at GEO, accession number GSE40787.

Supplementary Figure 8

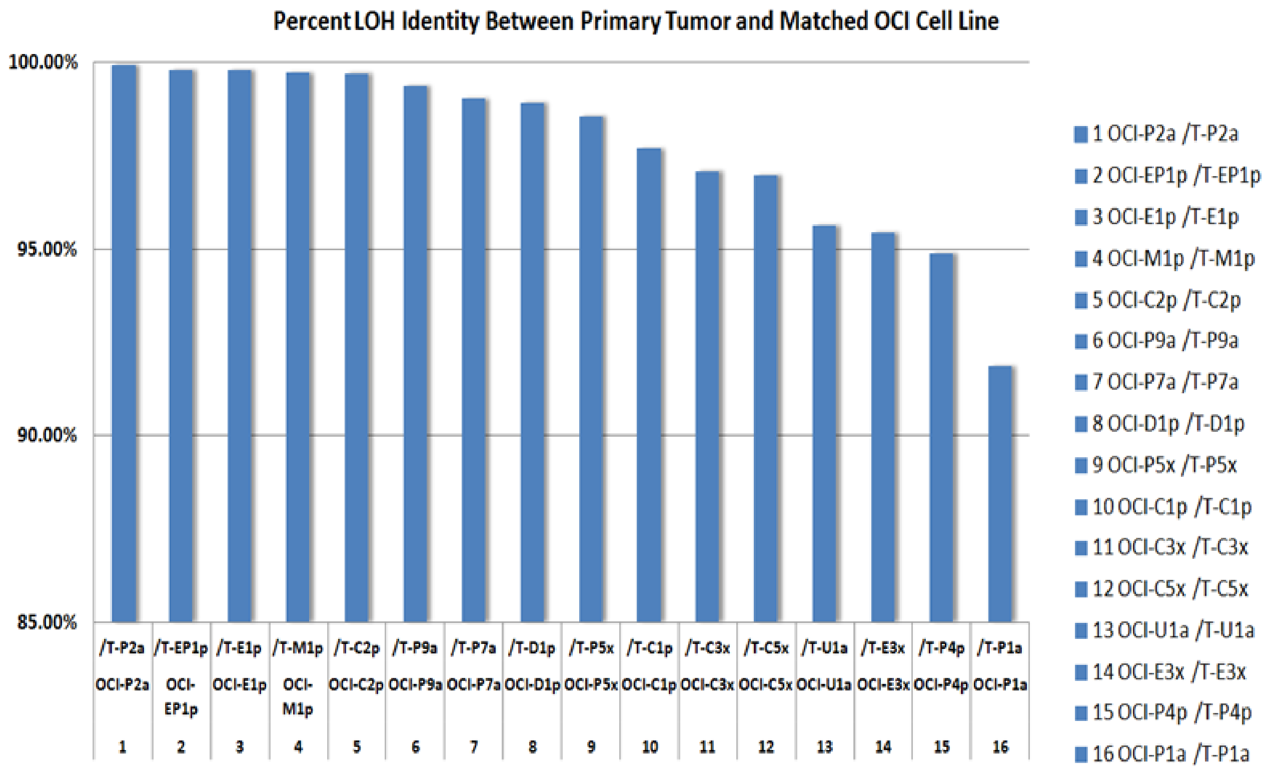

LOH analysis demonstrates similarity between cell line and tumor DNA

OCI lines have a greater than ninety percent LOH identity with the original tumor. We compared the genomic DNA of the uncultured tumor to the matched OCI cell line in sixteen cases. In the remaining cases we did not have adequate uncultured frozen tumor tissue to carry out such a comparison. To determine the similarity of the OCI cell lines with the matched tumors tissue, we performed pair-wise analyses of approximately 238,000 SNPs. SNP genotype calls were generated using the Affymetrix Genotyping Console version 4.1.4.840. The percent identical genotype calls for each pair was computed with the R v2.15.1 statistical programming language (R development core 2012; HTTP://cran.r-project.org/). See online Excel data file 1 for LOH segment and breakpoint analysis.

Supplementary Figure 9

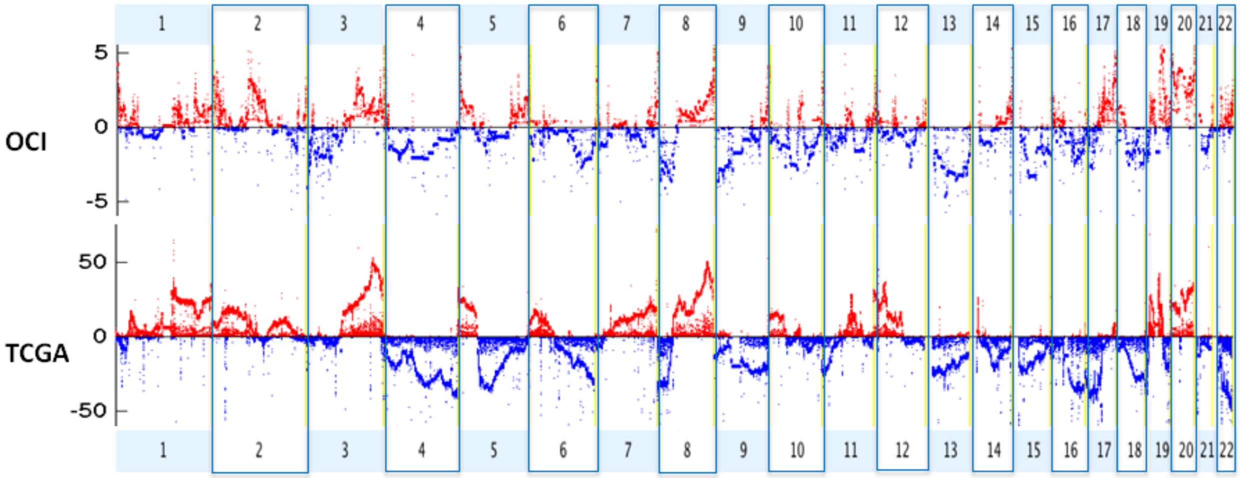

Copy number variations (CNV) of OCI cell lines are similar to ovarian tumors

In order to compare CNV patterns of OCI cells with the human ovarian tumors, we downloaded the TCGA CNV data from 100 randomly selected human ovarian tumors. This comparison revealed that the overall CVN trends are very similar between OCI lines and TCGA ovarian tumors. In both data sets CNV trend is copy number gain in chromosomes 2, 19, 20 and copy number loss in chromosomes 4, 9, 13, 15, and 18. While copy number losses were predominant in short arm of chromosomes 3 and 8, gains are predominant in the long arm, and this pattern was replicated in OCI lines. These results are consistent with the LOH comparison between OCI lines and their matched tumors and indicate that the genomic landscapes of primary tumors are preserved in OCI lines. The copy number analysis was performed using the Molecular Inversion Probe (MIP) 330k microarrays from Affymetrix on all 25 OCI lines. The results were merged into a copy number profile by using an algorithm that sums together the mean log2 intensity values in overlapping intervals. The red peaks indicate copy number gain and blue peaks indicate copy number loss. The complete dataset is available at GEO, accession number GSE40786.

Supplementary Figure 10

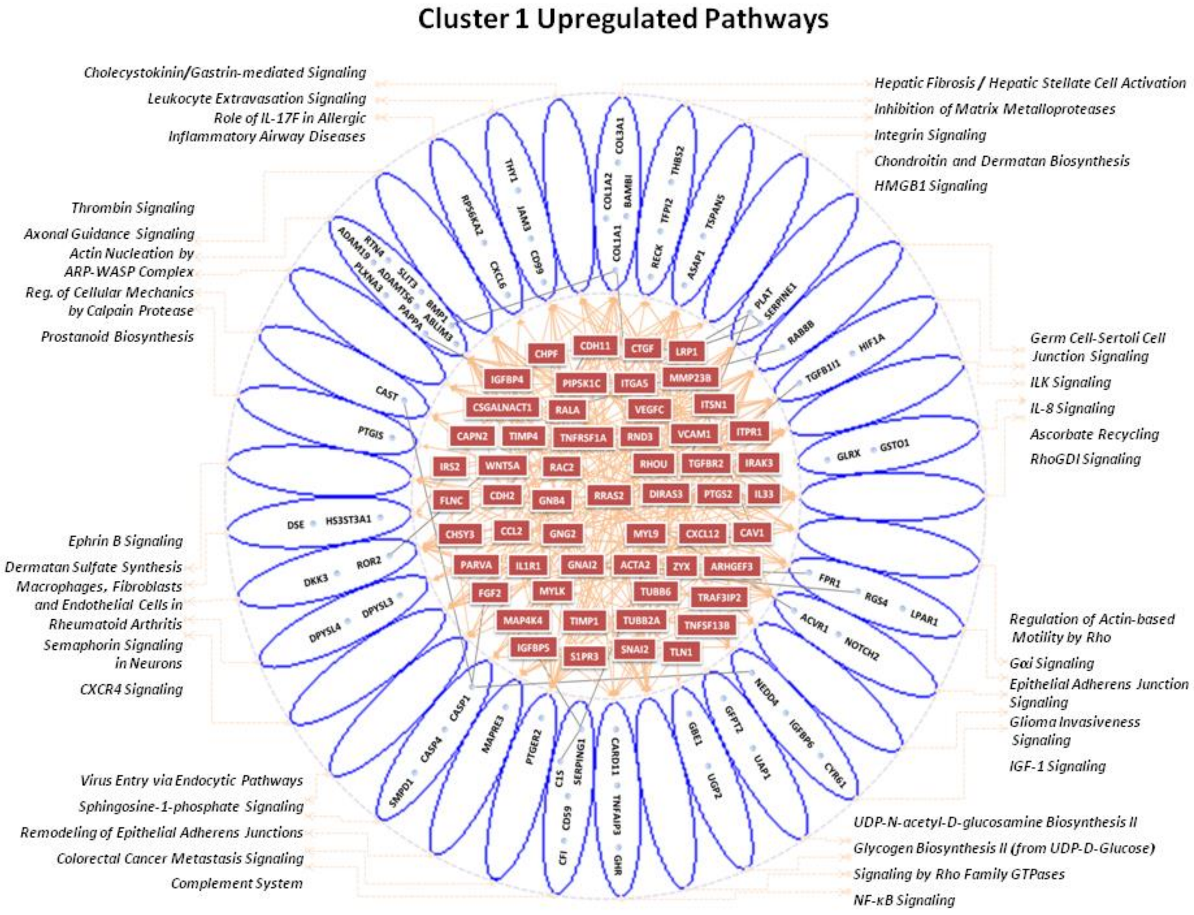

The Cluster 1 OCI cell lines are associated with distinct pathways

The 558 transcripts up-regulated in Cluster-1 are associated with 37 core pathways in IPA ($p < 0.05$). For the analysis depicted in this figure we used Ingenuity Pathway Analysis (IPA) software to organize the 823 genes that are differentially expressed between Cluster 1 vs. in Cluster 2 ($p < 0.05$) (Figure 3). See Supplementary Table 10 for the top 25 pathways, and online Excel data file 3 for the full list of IPA enriched pathways.

Supplementary Figure 11

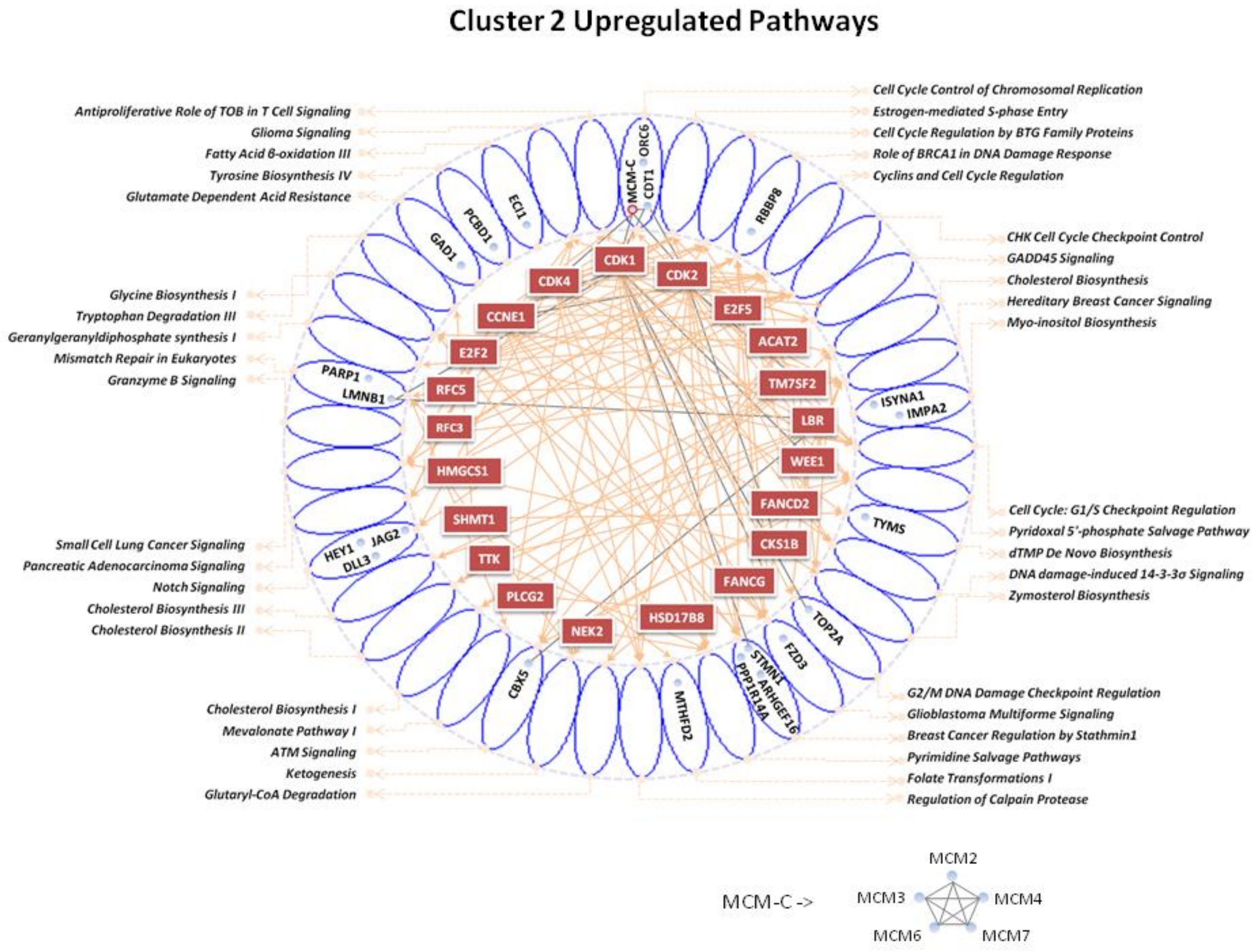

The Cluster 2 OCI cell lines are associated with distinct pathways

The 265 transcripts up-regulated in Cluster-2 are associated with 37 core pathways in IPA ($p < 0.05$). See Supplementary Table 11 for the top 25 pathways and online Excel data file 3 for the full list of IPA enriched pathways.

Supplementary Figure 12

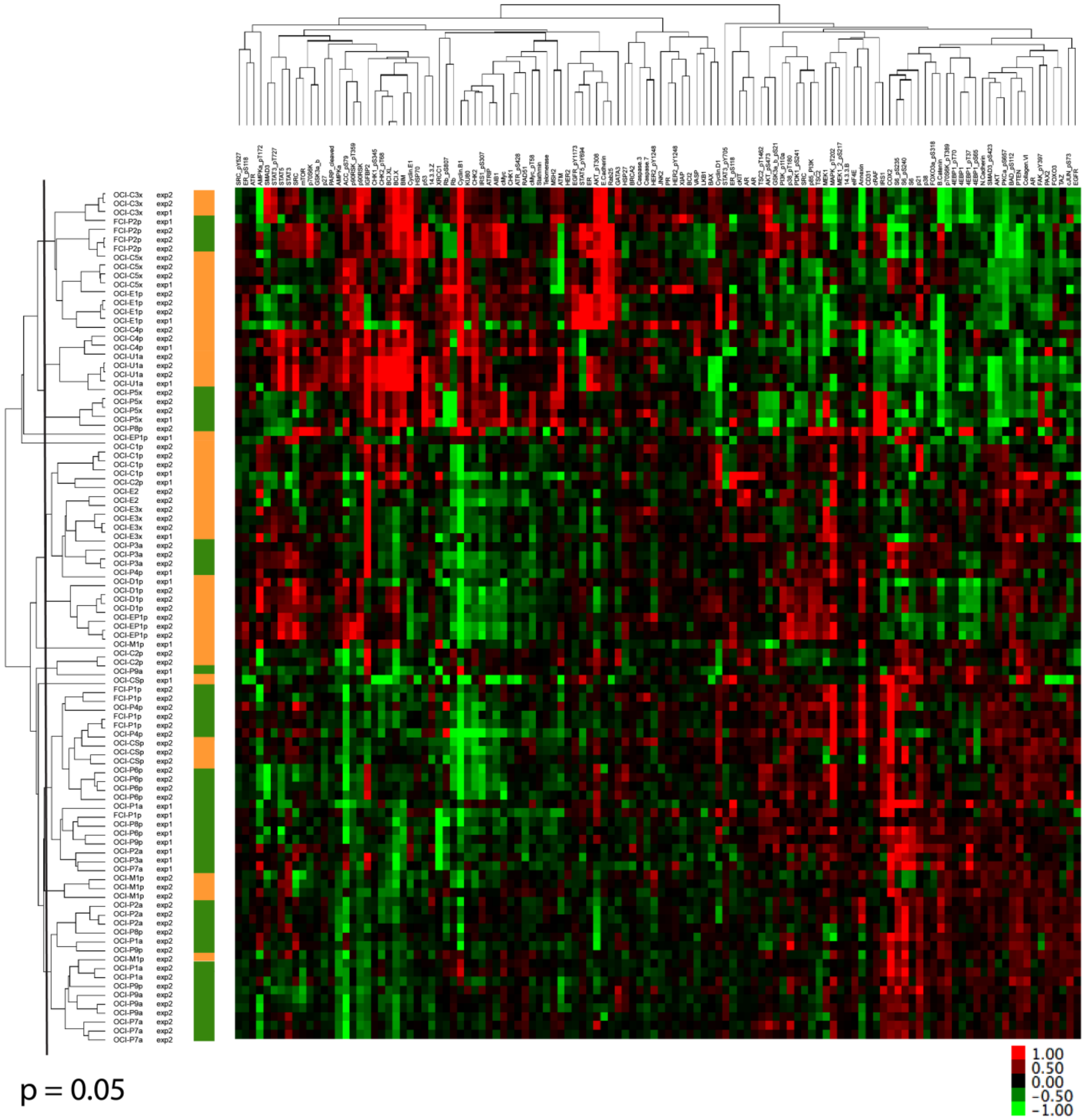

Protein expression profiles of OCI cell lines are reproducible between replicates

The heatmap profiles derived from unsupervised clustering of data from RPPA analysis of OCI cell lines. Each column depicts a different antibody and each row depicts an individual replicate from each cell line (n = 3-4). Tumor types are depicted in different colors; papillary serous (green), non-serous (orange). In order to ensure the reproducibility of the cell line phenotypes the protein extracts were prepared in triplicates from two different passages in two separate experiments. We observed that replicates of each cell line from different experiments and passages clustered together. See Supplementary Table 12 and online Excel file 6 for detailed histotype and replicate information and online Excel data file 5 for a complete list of antibodies.

Supplementary Figure 13

A

	Cell Line	Passage A	Passage B	Passage Difference	Cluster
1	OCI-P7a	13	24	11	1
2	OCI-P8p	18	30	12	1
3	OCI-P9a1	12	31	19	1
4	OCI-E3x	8	19	11	1
5	OCI-P5x	15	56	41	2
6	FCI-P2p	29	38	9	2
7	OCI-U1a	18	27	9	2
8	OCI-C4p	10	55	45	2
9	OCI-C5x	8	56	48	2
10	OCI-E1p	15	47	32	2

B

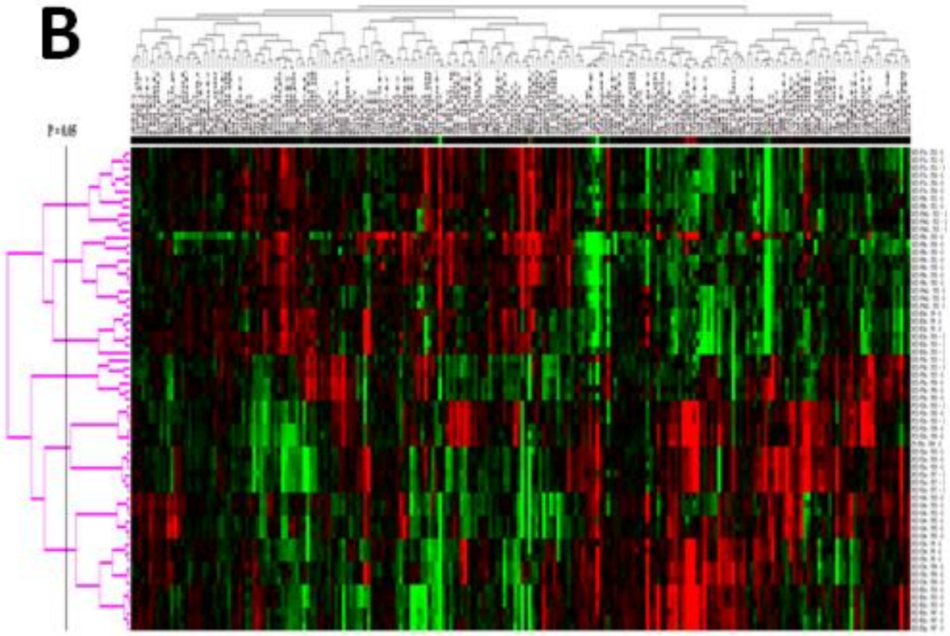

C

OCI-P7a. P13 -1
OCI-P7a. P13 -2
OCI-P7a. P13 - 3
OCI-P7a. P24 -1
OCI-P7a. P24 -2
OCI-P7a. P24 -3
OCI-P8p. P21 -1
OCI-P8p. P21 -2
OCI-P9a1. P12 - 2
OCI-P9a1. P12 - 1
OCI-P9a1. P12 - 3
OCI-P8p. P18 -1
OCI-P8p. P18 -2
OCI-P8p. P18 -3
OCI-P8p. P21 -3
OCI-P8p. P30 -1
OCI-P8p. P30 -2
OCI-P8p. P30 -3
OCI-P9a1. P31 -1
OCI-P9a1. P31 -2
OCI-P9a1. P31 -3
OCI-E3x. P8 -1
OCI-E3x. P8 -2
OCI-E3x. P8 -3
OCI-E3x. P19 - 2
OCI-E3x. P19 - 3
OCI-E3x. P19 - 1
OCI-P5x. P15 - 1
OCI-P5x. P15 - 2
OCI-P5x. P15 -3
OCI-P5x. P56 -1
OCI-P5x. P56 -2
OCI-P5x. P56 -3
FCI-P2p. P20 - 1
FCI-P2p. P20 -2
FCI-P2p. P20 - 3
FCI-P2p. P38 -1
FCI-P2p. P38 -2
FI-P2p. P38 -3
OCI-U1a. P18 -1
OCI-U1a. P18 -2
OCI-U1a. P18 -3
OCI-U1a. P27 - 2
OCI-U1a. P27 - 1
OCI-U1a. P27 - 3
OCI-C4p. P10 -2
OCI-C4p. P10 -1
OCI-C4p. P10 -3
OCI-C4p. P55 -1
OCI-C4p. P55 -2
OCI-C4p. P55 -3
OCI-C5x. P8 -1
OCI-C5x. P8 -2
OCI-C5x. P8 -3
OCI-C5x. P56 -1
OCI-C5x. P56 -2
OCI-C5x. P56 -3
OCI-E1p. P15 -1
OCI-E1p. P15 -2
OCI-E1p. P15 -3
OCI-E1p. P47 -1
OCI-E1p. P47 -2
OCI-E1p, P47 -3

Protein expression profiles of OCI lines are stable during long term culture

A) Ten OCI lines were cultured long term and triplicate aliquots were prepared at two different times with 9 to 48 passages separating them.

B) The unsupervised hierarchical clustering heatmap demonstrate that each cell line groups within the same cluster (1 vs. 2) regardless of passage number.

C) The cell lines, passages numbers and different replicates are listed in the order they clustered in the heatmap, demonstrating that the three replicates and two different passages of each cell line clustered together. The RPPA data set of 63 samples were probed with 218 antibodies.

Supplementary Figure 14

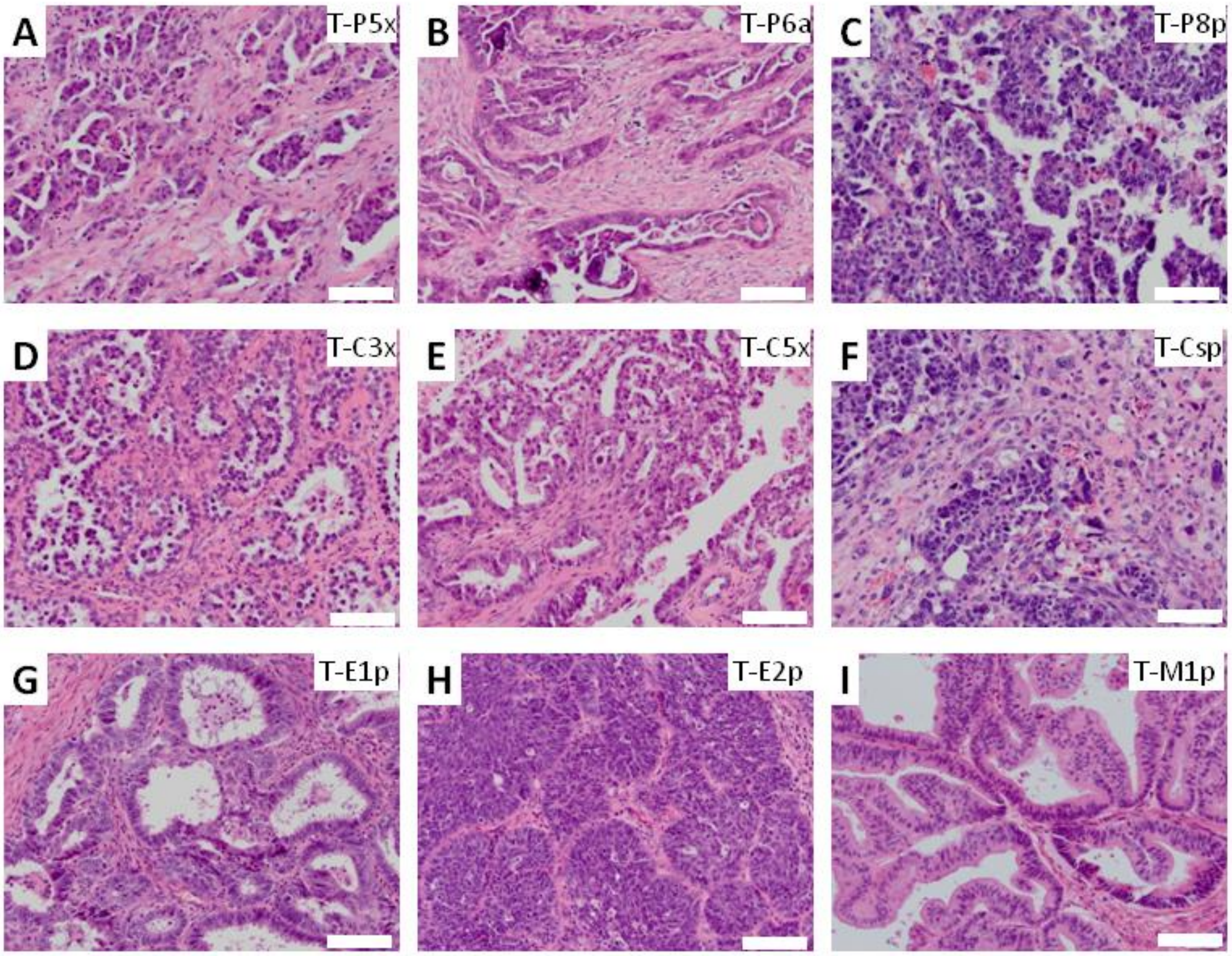

Histo-morphology of the primary tumors from which OCI lines were established

A) T-P5x, papillary serous adenocarcinoma, solid primary; **B)** T-P6a, papillary serous adenocarcinoma, ascites; **C)** T-P8x, papillary serous adenocarcinoma, solid primary, **D)** T-C3x, clear cell adenocarcinoma, primary xenograft; **E)** T-C5x, clear cell adenocarcinoma, primary xenograft; **F)** T-CSp, carcinosarcoma, solid primary; **G)** T-E1p, endometrioid adenocarcinoma, solid primary; **H)** T-E2p, endometrioid adenocarcinoma, solid primary; **I)** T-M1p, mucinous adenocarcinoma, solid primary. Scale bar (white) represents 100 μM.

Supplementary Figure 15

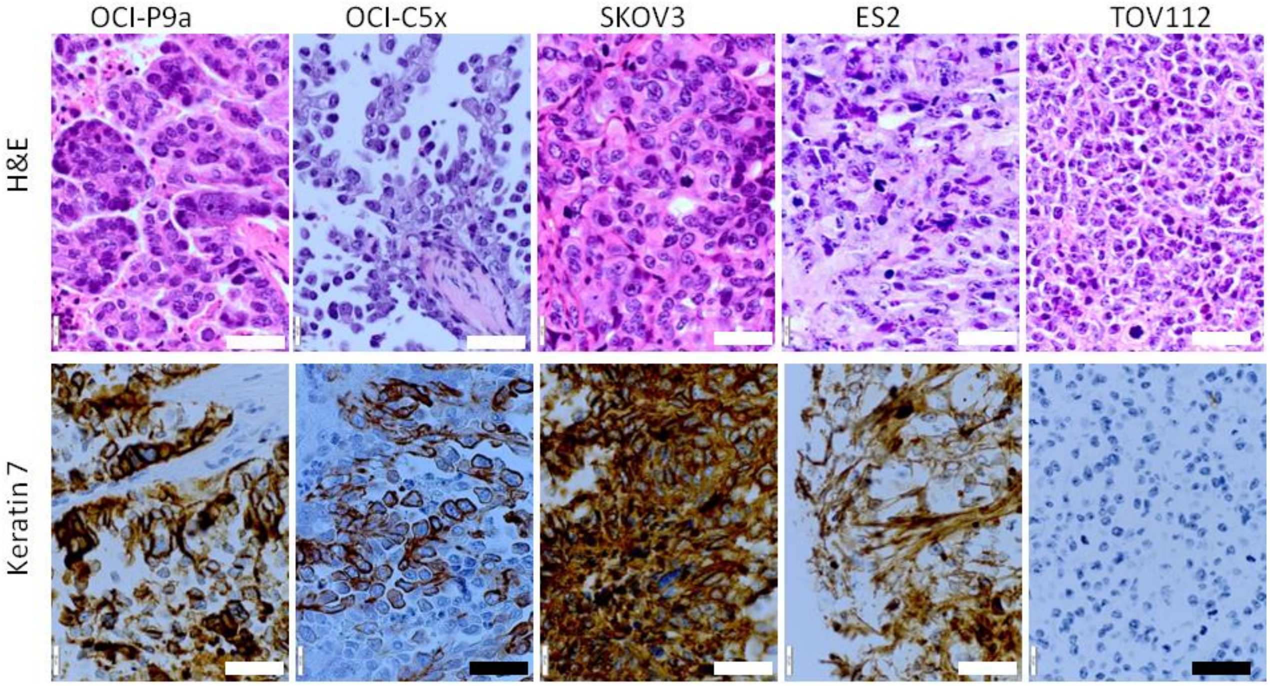

Histo-morphology of the OCI and SOC derived xenograft tumors

FFPE sections of xenograft tumors derived from OCI and SOC lines were stained with H&E and cytokeratin 7. The cytoplasmic staining (brown) in OCI-P9a, OCI-C5x and SKOV3 is specific. The stromal background staining in ES2 is non-specific. Scale bar (white) represents 40 μM.

Supplementary Figure 16

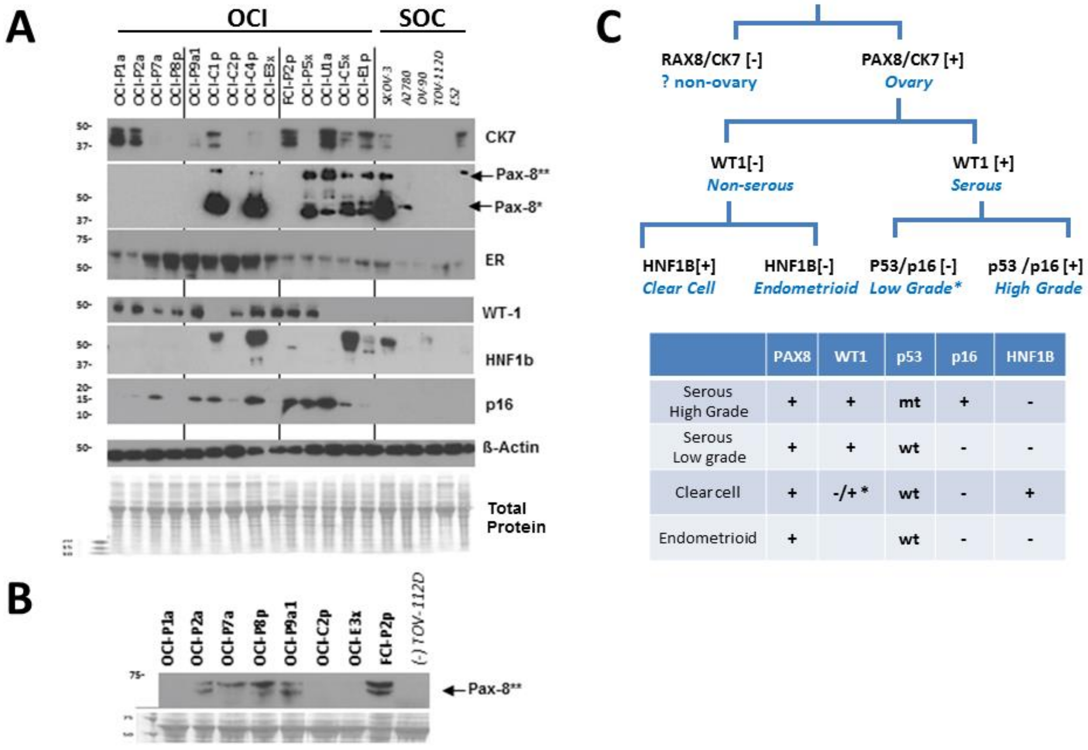

Ovarian cancer histotype specific marker expression of OCI and SOC lines

A) Equal amounts (25 µg) of OCI and SOC cell lysates were run in a western blot and probed with the following antibodies; keratin 7 (CK7), Pax8, estrogen receptor (ER), WT1, HNF1β, p16. Below, β- actin and total protein are shown as loading controls. High levels of both low molecular weight (*) and high molecular weight (**) isoforms of PAX8 are expressed in OCI-C1p, -C4p, -P5x, -U1a, -E1p, -C5x, and SKOV3. The names of the cell lines for each lane are listed above the western blot.

B) A separate western blot was run and developed with longer exposure demonstrating that OCI-P2a, -P7a, -P8p, P9a, and FCI-P2p do express predominantly high molecular weight (**) PAX8 isoforms. The names of the cell lines for each lane are listed above the western blot.

C) An algorithm for the interpretation of histotype specific markers is shown [6]. The marker profile of OCI lines match the expected histotype specific pattern (see Table 1). While the marker profile of SKOV3 (PAX8+, CK7+ and HNF1β+) is consistent with clear cell ovarian carcinoma (CCOC) in this western blot, the expression of HNF1 β is cytoplasmic and these cells express ARID1a (Fig. 5) which is inconsistent with CCOC. The marker expression patterns of the other SOC lines (A2780, OV90, TOV-112D, and ES2) do not match any of the histotype specific patterns. (*) Up to twenty percent CCOC can be WT1 positive [7].

Supplementary Figure 17

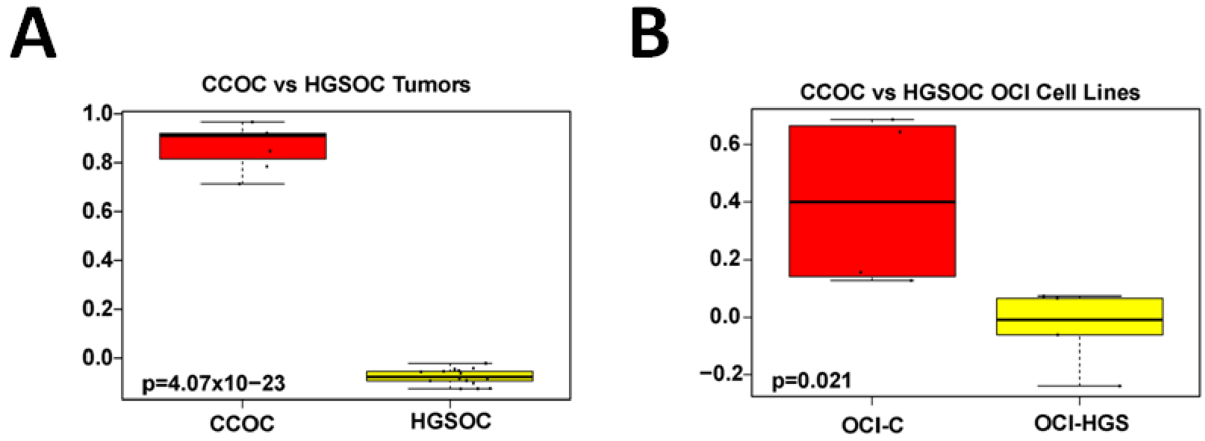

OCI cell lines recapitulate the HGSOC vs. CCOC specific tumor signatures

A) The top 25 genes that are most differentially expressed between clear cell (CCOC) and high grade serous (HGSOC) carcinomas were used to generate a gene signature. These genes are significantly upregulated in CCOC compared to HGSOC carcinomas (p= 4.07 x 10-23). Clear cell ovarian carcinoma (CCOC, red), High Grade Ovarian Carcinoma (HGSOC, yellow), inter-quartile range (colored box); the bar indicates the median value; whiskers show the range and are 1.5*IQR.

B) The 25 gene clear cell discriminator is expressed significantly differently between CCOC derived OCI cell lines (red) vs. HGSOC derived OCI cell lines (yellow), indicating that the most significant in vivo mRNA expression differences between CCOC vs. HGSOC patient tumors are at least partly preserved in the OCI cell lines (p=0.021). CCOC cell lines; OCI-C1p, C2p, C4p, C5x (OCI-C), HGSOC cell lines: OCI- P2a, P2p, U1a, P8p, and P5x (OCI- HGS)

Supplementary Figure 18

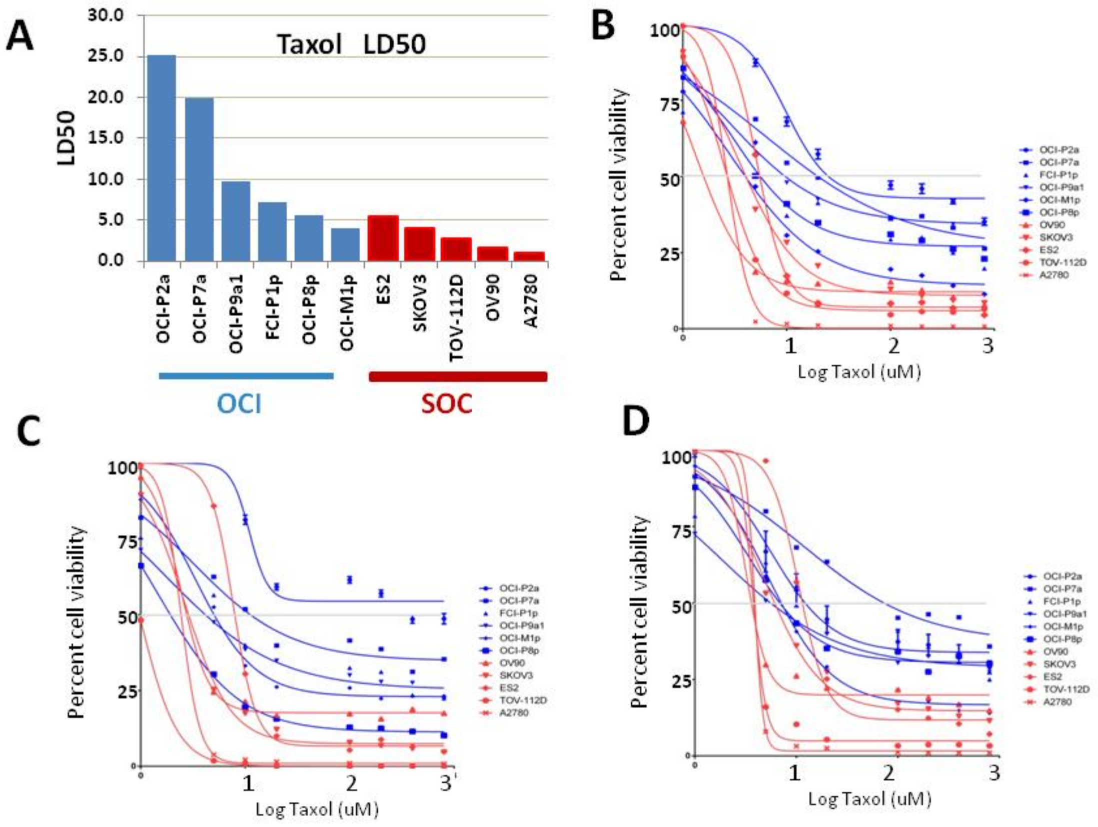

Taxol response of OCI lines (cluster 1) vs. SOC cell lines (cluster 2)

A) Representative of four different experiments of Taxol LD 50. Six replicates of the OCI (blue) and SOC (red) lines were plated in OCMI medium (3000 cells/well) in 96 well plates. Next, Taxol was added and metabolic activity was measured as 590/530 fluorescence via Alamar Blue after 5 days, which was used to estimate the LD50 for each line.

B-D) Full dose-response curves of three separate experiments plotted as percent cell viability of vehicle control vs. Log Taxol concentration (nM); OCI lines (blue), SOC lines (red). The LD90 for all of the OCI lines was >800 nM; in contrast LD90 for the SOC lines ranged between <1 nM (A2780), 5.2 nM (TOV-112D), 24 nM (ES2), 158 nM (SKOV3) to 800 nM (OV90). Error bars represent standard error of the mean of six replicates.

Supplementary Figure 19

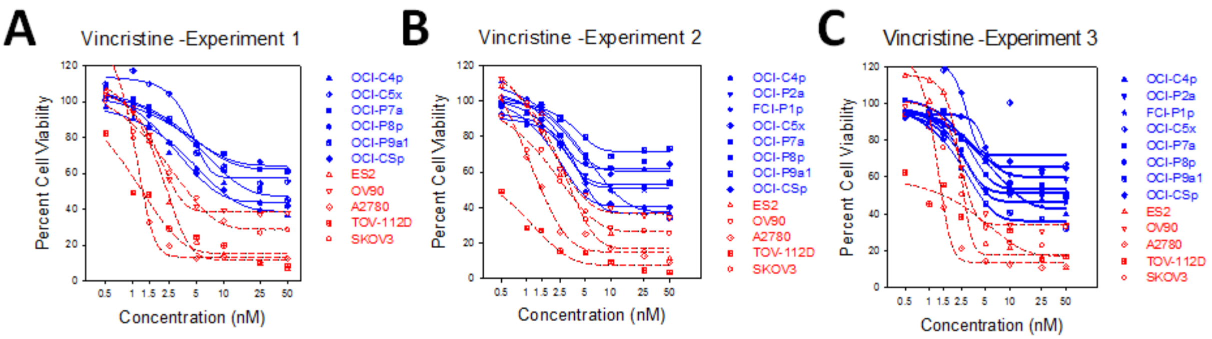

Vincristine response of cluster 1 OCI vs. Cluster 2 SOC/OCI cell line

Dose-response curves of the three independent Vincristine sensitivity experiments demonstrate that the cluster 1 OCI cell lines (blue) are more sensitive to Vincristine compared to cluster 2 SOC cell lines (red). Six replicates of the OCI and SOC lines were plated in OCMI medium (3000 cells/well) in 96 well plates. Error bars represent standard error of the mean

Both Taxol and Vincristine target the microtubules for their mechanism of action. However, while the vinca alkaloids bind stoichiometrically to tubulin subunits and induce polymer disassembly, the taxanes bind to polymerized tubulin and prevent disassembly. During cell division microtubules are assembled by polymerizing tubulin monomers, and are disassembled when they are no longer needed. Hence, both drugs block mitosis but through very different mechanisms.

Since taxanes and vinca alkaloids have very different mechanisms of actions, the control experiment with Vincristine suggests that the sensitivity of cluster 1 cell lines is related to a difference in tubulin function in cluster 1 vs. 2 cell lines and it is unlikely to be due to idiosyncratic metabolism, transport or inactivation of taxanes.

Supplementary Figure 20

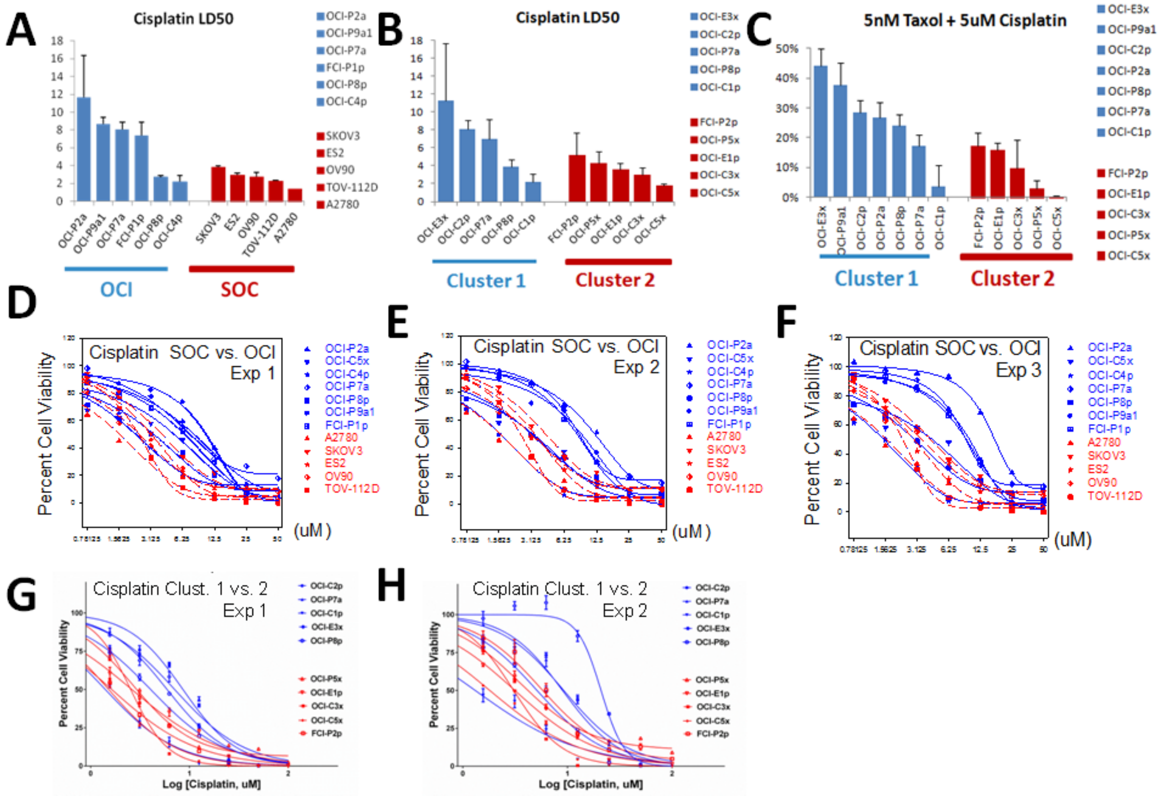

Cisplatin response of cluster 1 OCI vs. Cluster 2 SOC/OCI cell lines

Six replicates of the OCI and SOC lines were plated in OCMI medium (3000 cells/well) in 96 well plates. Next, Cisplatin was added and metabolic activity was measured as 590/530 fluorescence via Alamar Blue after 3 days. Error bars represent standard error of the mean of six replicates.

A) The bar graphs show average LD50 values for Cisplatin from three different experiments for OCI cluster 1 (blue bars) vs. cluster 2 SOC (red bars) cell lines.

B) The bar graphs show average LD50 values for Cisplatin from three different experiments for OCI Cluster 1 (blue) and OCI Cluster 2 (red) cell lines. Each cluster panel included one endometrioid, two clear cell and two papillary serous cell lines. Hence, the results are not due to histotype differences.

C) The bar graphs show average LD50 values for combined Cisplatin (5 uM) and Taxol (5 nM) treatment from three different experiments for OCI Cluster 1 (blue) and OCI Cluster 2 (red) cell lines. Each cluster panel included one endometrioid, two clear cell and two serous cell lines. Hence, the results are not due to histotype differences.

D) Dose-response curves of the three independent Cisplatin sensitivity experiments used to calculate the LD50 values in panel A. OCI cell lines (blue) and SOC cell lines (red).

E) Dose-response curves of Cisplatin sensitivity experiments used to calculate the LD50 values in panel B. OCI cluster 1 cell lines (blue) and OCI - cluster 2 cell lines (red).

Supplementary Figure 21

	Ince Cluster 1	Ince Cluster 2	TCGA Poor Prognosis	TCGA Good Prognosis	Tothill C1 up	Tothill C2 up	Tothill C3 up	Tothill C4 up	Tothill C5 up	Tothill C6 up	Vascular Content Signature
Ince Cluster 1	1										
Ince Cluster 2	-0.04	1									
TCGA Poor Prognosis	-0.04	0.18	1								
TCGA Good Prognosis	0.06	0.22	-0.60	1							
Tothill C1 up	0.93	-0.07	0.00	-0.02	1						
Tothill C2 up	0.36	0.12	-0.20	0.46	0.34	1					
Tothill C3 up	0.22	-0.06	-0.16	0.14	0.06	-0.12	1				
Tothill C4 up	-0.13	-0.03	-0.06	0.16	-0.22	0.18	0.19	1			
Tothill C5 up	-0.09	0.19	0.28	-0.20	-0.10	-0.50	-0.05	-0.53	1		
Tothill C6 up	0.45	0.22	0.06	0.03	0.35	-0.13	0.41	-0.13	0.42	1	
Vascular Content Signature	0.73	0.23	0.19	-0.11	0.74	0.05	0.07	-0.40	0.31	0.55	1

Correlation of Ince Cluster 1/2 vs. TCGA, Tothill and vascular content signatures

The gene expression microarray data for the Ince et al ovarian cancer cell lines were downloaded from the Gene Expression Omnibus (GSE40785). In total, 37 microarrays and 47,323 unique probes were retrieved. All data were log2 transformed with Microsoft Excel 2010, quantile normalized with R statistical software version 3.1 with the package 'ArrayTools'. The data were then filtered with Cluster v3.0 and all probes with standard deviations of less than 0.5 across all samples were removed, leaving 7,479 probes. These 7,479 probes from the 37 arrays were then median centered in Cluster v3.0 and hierarchical clustered, where two major groups of ovarian cancer cell lines were observed (Ince Cluster 1 and 2). The top 500 probes over-expressed in each group (FDR <5) were then used to generate gene signature scores for each tumor in the TCGA ovarian cancer gene expression dataset. Signature scores for other relevant gene expression signatures were also identified for each tumor in the TCGA dataset including the TCGA defined 'poor' and 'good' prognosis genes, the Tothill C1-C6 'up' genes, and a recently described 'vascular content' gene expression signature. All signature scores were identified by taking the mean value for all genes within a respective signature. Excel was then used to identify the Pearson correlation values for each of the signature scores. This analysis showed a very strong correlation with the genes most abundantly expressed in the Ince Cluster 1 ovarian cell lines and the Tothill C1 up genes (R value = 0.93). Interestingly, both these signatures also exhibited a strong correlation with genes that are abundantly expressed in vascular endothelial cells (>0.7) [8], suggesting that both of these signatures define ovarian cancer cells with mesenchymal/endothelial attributes.

Supplementary Figure 22

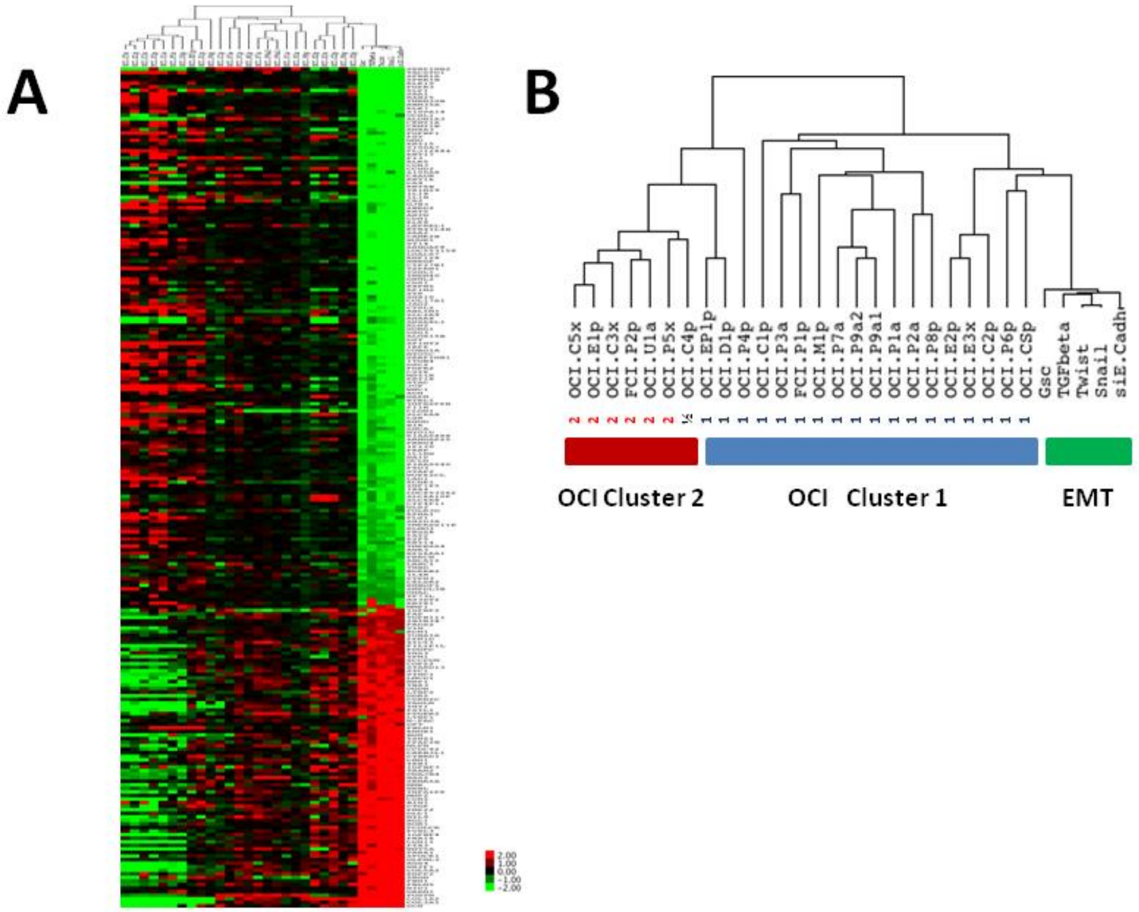

Epithelial and mesenchymal marker expression in OCI lines

A) Unsupervised hierarchical clustering of mRNA expression of OCI ovarian cancer cell lines and HMLE cell lines induced to undergo EMT. Each row represents an mRNA from the Taube EMT core signature. Each column shows the mRNA levels for each sample; the samples on the left are the cultured OCI lines and the samples on the right are the manipulations to HMLE cells from Taube et al. Both data sets are log2 transformed for visualization. All of the Cluster 2 OCI samples were associated with high levels of epithelial markers and low levels of mesenchymal markers. In contrast, fifteen Cluster 1 OCI lines co-cluster with the EMT signature. Of these, five lines (OCI-E2-, OCI-E3x, OCI-C2p, OCI-P6p, and OCI-CSp) correlated significantly ($p<0.00004551$, Pearson correlation) with the EMT signature, suggesting that these are the most mesenchymal in nature. OCI lines display a spectrum phenotypes from weakly epithelial to more mesenchymal.

B) Dendogram of the cell lines that make up the two clusters in panel A.

Supplementary Table 1

Clinical features of the primary tumors from which SOC cell lines were initiated

The standard ovarian cancer (**SOC**) cell line panel was selected to represent examples of different tumor subtypes, including papillary serous (OV90), clear cell (ES2, RMG-1), mucinous (RMUG-L), and endometrioid (TOV-112D).

CELL LINE	SOURCE	DIAGNOSIS	YEAR	TISSUE	Reference
**SKOV3**	ATCC& ECACC	adenocarcinoma	1973	ascites	ATCC
**A2780**	ECACC	ovarian carcinoma	Pre-1985	-	ECACC
**TOV-112D**	ATCC	endometrioid, grade 3	1992	primary	ATCC
**OV90**	ATCC	papillary serous, grade 3	1992	ascites	ATCC
**ES2**	ATCC	clear cell carcinoma, grade3	Pre-1990	primary	ATCC

Supplementary Table 2

Twenty-five ovarian cell lines (OCI) are established from different tumor subtypes

The most common histotype represented is papillary serous. In addition, clear cell, endometrioid, mucinous, carcinosarcoma, and dysgerminoma histotypes were established as cell lines. Tissue sources were primary solid tumors, malignant ascites fluid or from xenograft tumors. The cell lines were assessed in 2D, 3D soft agar colonies, and as xenografts in immunocompromised mice.

Cell Line	Histotype	Sample Origin	Soft Agar Colony	Xeno-graft Tumor	Fresh Sample	Frozen Sample
**OCI-C1p**	clear cell	Primary	Yes	No		+
**OCI-C2p**	clear cell	Primary	Yes	No		+
**OCI-C3x**	clear cell	Xenograft	Yes	No	+	
**OCI-C4p**	clear cell	Primary	Yes	No	+	
**OCI-C5x**	clear cell	Xenograft	Yes	**Yes**		+
**OCI-P1a**	papillary serous	Ascites	Yes	No	+	
**OCI-P2a**	papillary serous	Ascites	Yes	No	+	
**OCI-P3a**	papillary serous	Ascites	Yes	n/a	+	
**OCI-P4p**	papillary serous	Primary	Yes	n/a	+	
**OCI-P5x**	papillary serous	Xenograft	Yes	**Yes**	+	
**OCI-P6p**	papillary serous	Primary	Yes	n/a	+	
**OCI-P7a**	papillary serous	Ascites	Yes	No	+	
**OCI-P8p**	papillary serous	Primary	Yes	n/a	+	
**OCI-P9a**	papillary serous	Ascites	Yes	**Yes**	+	
**OCI-P9p**	papillary serous	Primary	Yes	**Yes**	+	
**FCI-P1p**	papillary serous	Primary	Yes	n/a	+	
**FCI-P2p**	papillary serous	Primary	Yes	No	+	
**OCI-E1p**	endometrioid	Primary	Yes	**Yes**	+	
**OCI-E2p**	endometrioid	Primary	Yes	No	+	
**OCI-E3x**	endometrioid	Xenograft	Yes	n/a		+
**OCI-EP1p**	endo-serous	Primary	Yes	No	+	
**OCI-M1p**	mucinous	Primary	Yes	No	+	
**OCI-U1a**	mullerian	Ascites	Yes	**Yes**	+	
**OCI-CSp**	carcinosarcoma	Primary	Yes	**Yes**	+	
**OCI-D1p**	dysgerminoma	Primary	Yes	No		+

Supplementary Table 3

The culture conditions of twenty-five ovarian tumor cell lines (OCI)

Please note that some OCI lines are cultured in 21% O_2 and others in 5% O_2. In addition, while some OCI lines are cultured best in standard culture plates, others require Primaria culture plates. In general, the endometrioid and mucinous carcinoma cell lines are cultured in 5% Os and with additional 100nM 17β- Estradiol (OCMI-e). All cell lines have been cultured for at least 15 passages, > 20 passages (n=24), > 25 passages (n=12), > 30 passages (n=7), >40 passages (n=5). Each passage corresponds to approximately 2 to 3 population doublings.

	Cell Line Name	Cluster	Minimum Passages	Culture plate	Medium	O2 %
1	OCI-C4p	1	61	Primaria	OCMI	21
2	OCI-P8p	1	40	Standard	OCMI	5
3	OCI-P9a1	1	31	Standard	OCMI	5
4	OCI-P4p	1	26	Standard	OCMI	21
5	OCI-P2a	1	26	Standard	OCMI	5
6	FCI-P1p	1	26	Standard	OCMI	5
7	OCI-P7a	1	24	Standard	OCMI	5
8	OCI-EP1p	1	24	Primaria	OCMI	5
9	OCI-D1p	1	23	Standard	OCMI	21
10	OCI-C1p	1	23	Standard	OCMI	21
11	OCI-P1a	1	23	Standard	OCMI	5
12	OCI-CSp	1	22	Primaria	OCMI	21
13	OCI-C2p	1	21	Standard	OCMI	5
14	OCI-P3a	1	20	Standard	OCMI	21
15	OCI-P9a2	1	20	Primaria	OCMI	21
16	OCI-P6p	1	15	Standard	OCMI	21
17	OCI-E2p	1	20	Standard	OCMI-e	5
18	OCI-E3x	1	21	Standard	OCMI-e	5
19	OCI-M1p	1	25	Primaria	OCMI-e	5
20	OCI-P5x	2	56	Primaria	OCMI	21
21	OCI-C5x	2	100	Primaria	OCMI	21
22	OCI-E1p	2	47	Primaria	OCMI-e	5
23	FCI-P2p	2	38	Primaria	OCMI	21
24	OCI-U1a	2	27	Primaria	OCMI	21
25	OCI-C3x	2	23	Primaria	OCMI	21

Supplementary Table 4

The list of causes that prevent successful culture of tumor cells

During the development and optimization of OCMI medium and culture methods, we failed to establish cell lines in some cases. In two cases, the tissue sample was necrotic and no viable cells were detected upon plating. In four cases the tissue sample was composed of normal and stromal tissues, and no tumor cells were detected. In two cases bacterial/fungal contamination was detected within the first weeks of culture. In these cases we had to terminate the cultures. In addition, during the preliminary phase, we used five endometrioid adenocarcinomas and one mucinous adenocarcinoma to optimize the medium formulation. These specimens did not survive due to suboptimal medium formulations and culture conditions such as 21% O2 that was initially used for endometrioid and mucinous tumors. Upon fully optimizing the culture methods, we failed to establish a long-term continuous culture only once with a viable sample that contained tumor tissue (OC-P10).

	Cell Line	Histopathological Subtype	
1	**OC-E13**	Endometrioid w. mucinous differentiation	Suboptimal Medium
2	**OC-E14**	Low grade endometrioid	Suboptimal Medium
3	**OC-E9**	Mucinous adenocarcinoma	Suboptimal Medium
4	**OC-E10**	Endometrioid	Suboptimal Medium
5	**OC-E6**	Endometrioid, solid and ascites	Suboptimal Medium
6	**OC-P11**	Papillary serous	Non-viable sample
7	**OC-E7**	Mucinous	No Tumor in Sample
8	**OC-E11**	Endometrioid	No Tumor in Sample
9	**OC-E15**	Mucinous / endometrioid	No Tumor in Sample
10	**FC-P3**	Papillary serous	No Tumor in Sample
11	**FC-P4**	Papillary serous	Bacterial Fungal contamination
12	**OC-E8**	Endometrioid	Bacterial Fungal contamination
13	**OC-P9**	Papillary serous	Non-viable sample
14	**OC-P10**	Papillary serous	Failure in Optimal Conditions

Supplementary Table 5

The mitochondrial hyper-variable region (HVR1) fingerprint for 16 cell lines

PCR product of D-Loop mtDNA has length 1450 bp (HVR1: 15756-16569bp). Sequence analysis has shown polymorphisms for 37 nucleotides in HVR1 between 15824bp and 16519bp. (Dots indicate no change from the reference sequence; CRS – Cambridge Reference Sequence; D – deletion; N – sequence not available; blank- no nucleotide present).

(A) Location in HVR1 (15756-16569)		15824	15927	15928	16093	16111	16114	16124	16126	16145	16173	16176	16182	16183	16189	16194	16195	16218	16223	16224	16239	16257	16262	16266	16278	16290	16293	16294	16296	16299	16304	16311	16319	16327	16362	16390	16474	16519
	**CRS**	**A**	**G**	**G**	**T**	**C**	**C**	**T**	**T**	**G**	**C**	**C**	**A**	**A**	**T**	**A**	**T**	**C**	**C**	**T**	**C**	**C**	**C**	**C**	**C**	**C**	**A**	**C**	**C**	**A**	**T**	**T**	**G**	**C**	**T**	**G**	**G**	**T**
Cell Line	FCI-P1p	.	.	.	.	.	.	.	.	.	.	.	.	.	.	.	.	.	.	.	.	.	.	.	.	.	.	.	.	.	.	.	.	.	.	.	.	.
	A2780	.	.	.	.	.	.	C	.	.	.	.	.	.	.	.	.	.	T	.	.	.	.	.	.	.	.	.	.	.	.	.	A	.	.	.	.	.
	SKOV3	.	.	.	.	.	.	.	.	A	.	A	.	.	.	.	.	.	.	.	.	.	.	.	.	.	.	.	.	.	.	.	.	.	.	A	.	C
	BPLER3	G	.	.	.	.	.	C	.	.	.	.	.	.	.	.	.	.	T	.	.	.	.	.	T	.	.	.	.	.	.	.	.	.	C	.	.	C
	OCI-P7a	.	.	.	.	T	.	.	.	.	.	.	.	.	.	.	.	T	T	.	A	.	.	T	.	T	.	.	.	.	.	.	A	.	C	.	.	.
	OCI-P9a1	.	.	.	.	.	.	.	.	.	.	.	.	.	.	.	.	.	.	.	.	.	.	.	.	.	.	.	.	.	.	.	.	.	.	.	.	.
	OCI-C5x	.	.	.	.	.	.	.	.	.	.	.	.	.	.	.	.	.	.	.	.	.	.	.	.	.	.	.	.	.	.	.	.	.	.	.	.	.
	OCI-P3a	.	.	.	.	.	.	.	.	.	.	.	.	.	.	.	.	.	.	.	.	.	.	.	.	.	.	.	.	.	.	.	.	.	.	.	.	C
	OCI-P8P	.	.	.	.	.	.	.	.	.	.	.	.	.	.	.	.	.	.	.	T	.	.	.	.	.	.	.	.	.	.	.	.	.	.	.	.	C
	OCI-C3x	.	.	.	.	.	.	.	.	.	.	.	.	.	.	.	.	.	.	.	.	.	.	.	.	.	.	.	.	.	.	C	.	.	.	.	.	C
	OCI-P2a	.	.	A	.	T	.	.	C	.	.	.	.	.	.	.	.	.	.	.	.	.	.	.	.	.	.	T	T	.	C	C	.	T	.	.	.	C
	ES2	.	.	.	C	.	.	.	.	.	.	.	.	C	C	C	C	.	.	.	.	D	D	.	.	.	G	.	.	.	.	.	.	.	.	.	.	C
	TOV-112D	.	.	.	.	.	.	.	.	.	.	.	.	.	.	.	.	.	.	C	.	.	.	.	.	.	.	.	.	.	.	C	.	.	.	.	.	C
	OV9O	.	A	.	.	.	.	.	.	.	T	.	C	C	C	C	C	.	.	.	.	.	.	.	T	.	.	.	.	.	.	.	.	.	.	.	.	C
	OCI-C4P	.	.	.	.	.	T	.	.	.	.	.	.	.	.	.	.	.	.	.	.	.	.	.	.	.	.	.	.	G	.	.	.	.	.	.	.	C
	FCI-P2p	.	.	.	.	.	.	.	.	.	.	.	.	.	.	.	.	.	.	.	.	.	.	.	.	.	.	.	.	.	.	.	.	.	.	.	D	C

Supplementary Table 6

The mitochondrial hyper-variable region 2 (HVR2) fingerprint for 16 cell lines

PCR product of D-Loop mtDNA has length 1450 bp (HVR2: 1-636 bp). Sequence analysis has shown polymorphisms for 29 nucleotides in HVR2 between 1 and 636 bp. (Dots indicate no change from the reference sequence; CRS – Cambridge Reference Sequence; Ins Loc – location of insertion is indicated after the decimal point; D – deletion; N – sequence not available; blank- no nucleotide present)

(B) Location in HVR2 (1-636)		064	073	146	150	152	153	188	195	225	226	235	263	264	306	307	308	309	309	309	310	315	408	494	497	523	524	525	525	589
	**Ins Loc**																		.1	.2		.1					.1		.1	
	**CRS**	C	A	T	C	T	A	A	T	G	T	A	A	C	C	C	C	C	—	—	T	—	T	C	C	A	—	C	—	T
Cell Line	FCI-P1p	.	.	.	.	.	.	.	.	.	.	.	G	.	.	.	.	.	—	—	.	C	.	.	.	.	—	.	—	D
Cell Line	A2780	.	G	.	T	C	.	.	.	.	.	.	G	.	.	.	.	.	—	—	.	C	.	.	.	D	—	D	—	D
Cell Line	SKOV3	.	G	.	.	C	.	.	.	.	.	.	G	.	.	.	.	.	C	—	.	C	.	.	.	.	—	.	—	.
Cell Line	BPLER3	.	G	.	.	.	.	.	.	.	.	G	G	.	.	.	.	.	—	—	.	C	.	.	.	.	—	.	—	.
Cell Line	OCI-P7a	T	G	C	.	.	G	.	.	.	.	G	G	.	.	.	.	.	C	—	.	C	.	.	.	N	—	N	—	N
Cell Line	OCI-P9a1	.	G	.	.	.	.	.	.	.	.	.	G	.	.	.	.	.	C	—	.	C	.	.	.	D	—	D	—	D
Cell Line	OCI-C5x	.	.	.	.	.	.	.	.	.	.	.	G	.	.	.	.	.	C	—	.	C	.	.	.	.	—	.	—	.
Cell Line	OCI-P3a	.	.	.	.	.	.	.	.	.	.	.	G	.	.	.	.	.	—	—	.	C	.	.	.	.	—	.	—	.
Cell Line	OCI-P8P	.	.	.	.	.	.	.	.	.	.	.	G	.	.	.	.	.	C	—	.	C	.	.	.	.	—	.	—	.
Cell Line	OCI-C3x	.	G	.	.	.	.	.	.	.	.	.	G	.	.	.	.	.	C	—	.	C	.	.	T	.	A	.	C	D
Cell Line	OCI-P2a	.	G	.	.	.	.	.	.	.	.	.	G	.	.	.	.	.	—	—	.	C	.	.	.	.	—	.	—	.
Cell Line	ES2	.	.	.	.	C	.	.	.	.	.	.	G	.	.	D	D	D	—	—	.	C	.	.	.	.	—	.	—	D
Cell Line	TOV-112D	.	G	.	.	.	.	G	.	.	.	.	G	T	.	.	.	.	—	—	.	C	.	.	T	.	A	.	C	D
Cell Line	OV9O	.	G	.	.	.	G	.	C	A	C	.	G	.	D	D	D	D	—	—	.	C	A	.	.	.	—	.	—	D
Cell Line	OCI-C4P	.	.	.	.	.	.	.	.	.	.	.	G	.	.	.	.	.	C	C	.	C	.	D	.	.	—	.	—	.
Cell Line	FCI-P2p	.	.	.	.	C	.	.	.	.	.	.	G	.	.	.	D	D	—	—	C	—	.	.	.	.	—	.	—	D

Supplementary Table 7

The short tandem repeat (STR) profile of OCI cell lines

The OCI cell lines were validated by STR DNA fingerprinting at the MD Anderson Characterized Cell Line Core (CCLC). The STR profiles were compared to online search databases (DSMZ/ATCC/JCRB/RIKEN) of 2455 known cell line profiles; along with the MD Anderson Characterized Cell Line Core (CCLC) database of 2556 known profiles. The analysis demonstrated that the OCI line STR profiles were unique and did not match any of the known cell lines. In one case the STR profile of OCI-C2p matched with the breast cancer cell line UACC2715. However, we have demonstrated that an OCI-C2p cell line has a > 95% SNP identity with its matching uncultured original tumor, confirming it's identify. Furthermore, we have never obtained or cultured UACC2715 cell line in our laboratory, making cross-contamination or switch impossible. Hence, the similarity to is likely to be incidental.

Cell Lines	AMEL	CSF1PO	D13S317	D16S539	D5S818	D7S820	TH01	TPOX	vWA	Comments
OCI-C1P	X	10,11	11,13	11,12	12	10,12	7,9.3	9,11	16,17	Unique Profile
OCI-C4P	X	10	11	12	11,13	10,11	6,7	8,9	17	Unique Profile
OC1-C5x	X	11,13	10,11,12	10,11,12	10,11	8,12	7,9.3	11	18,22	Unique Profile
OCI-P1a	X	12	9,13	12,14	11,13	11,12	9.3	11,12	16,20	Unique Profile
OC1-P2a	X	11	11,12	12,13	12	9,11	9,9.3	8	16	Unique Profile
OCI-P5X	X	12	11	11	10,12	12	6	9,11	14,17	Unique Profile
OCI-P7a	X	10,12	11	8,14	12	11,12	6,10	9,11	15,18	Unique Profile
OCI-P8p	X	11	11,12	9	11,13	9,10	7,9.3	9,11	15,17	Unique Profile
OC1-P9a1	X	10,11	9,13	9	11	9,14	9.3	8	17,19	Unique Profile
FCI-P2p	X	10,13	10	12	11	9,10	6,9	11	14,17	Unique Profile
OCI-E1p	X	12,13	11	11,12	11,12	11,12	6,9.3	8,12	14,19	Unique Profile
OCI-EP1P	X	12,13	11,14	9,11	16,17	10,13	9,9.3	8,9	16,19	Unique Profile
OCI-E3x	X	10,11	8,10	11	12,13	8,9	7,9	8,11	14,18	Unique Profile
OCI-M1P	X	10,12	12	11	16,17	11	9	8,9	18	Unique Profile
OCI-U1A	X	10,14	8,10	12,13	12	10,11	9,9.3	8	16,17	Unique Profile
OCI-C2P	X	11,12	11,12	8,13	10,12	9,11	7,9	8,11	17,18	***

Supplementary Table 8
List of 20 most up and down regulated mRNAs in Cluster 1
See online Excel data file 2 and Gene Expression Omnibus (GSE40785).

Cluster#1, Up regulated genes		fold
HSD11B1	hydroxysteroid (11-beta) dehydrogenase 1	32.20
DIRAS3	DIRAS family, GTP-binding RAS-like 3	27.35
HSD11B1	hydroxysteroid (11-beta) dehydrogenase 1	23.89
HSD11B1	hydroxysteroid (11-beta) dehydrogenase 1	22.85
COL1A2	collagen, type I, alpha 2	22.61
COL1A2	collagen, type I, alpha 2	19.01
COL1A1	collagen, type I, alpha 1	12.53
THBS2	thrombospondin 2	11.51
ANXA8	annexin A8	11.46
BDKRB1	bradykinin receptor B1	11.34
RARRES1	retinoic acid receptor responder 1	9.23
COL5A1	collagen, type V, alpha 1	9.11
C13orf33	chromosome 13 open reading frame 33	8.58
ANXA8	annexin A8 (ANXA8)	8.40
SPARC	secreted protein, acidic, cysteine-rich (osteonectin)	8.27
SERPINE1	serpin peptidase inhibitor 1	8.18
CPA4	carboxypeptidase A4	7.85
RARRES1	retinoic acid receptor responder 1	7.80
WISP1	WNT1 inducible signaling pathway protein 1	7.74
LOC652846	Similar to Annexin A8 (Vascular anticoagulant-beta)	7.53

Cluster #1, Down regulated genes		fold
CD24	CD24 molecule (CD24)	0.17
QPRT	quinolinate phosphoribosyltransferase	0.29
IGF2BP3	insulin-like growth factor 2 mRNA binding protein 3	0.32
PITX1	paired-like homeodomain transcription factor 1	0.35
SPINT2	serine peptidase inhibitor, Kunitz type, 2	0.38
IMPA2	inositol(myo)-1(or 4)-monophosphatase 2	0.40
CLDN1	claudin 1	0.41
CTSL2	cathepsin L2	0.41
HIST1H4C	histone cluster 1, H4c	0.43
ABLIM1	actin binding LIM protein 1	0.43
AURKB	aurora kinase B	0.43
CMTM8	CKLF-like MARVEL TM 8	0.43
SDC1	syndecan 1 (SDC1), transcript variant 1	0.44
UBE2C	ubiquitin-conjugating enzyme E2C	0.44
AIF1L	allograft inflammatory factor 1-like	0.44
HES4	hairy and enhancer of split 4 (Drosophila)	0.44
UBE2C	ubiquitin-conjugating enzyme E2C	0.44
CDCA5	cell division cycle associated 5	0.44
DBNDD1	dysbindin, dystrobrevin binding protein 1	0.44
CDCA7	cell division cycle associated 7	0.45

Supplementary Table 9
List of 20 most up and down regulated mRNAs in Cluster 2

See online Excel data file 2 for details. The complete dataset is available at Gene Expression Omnibus (GSE40785).

Cluster #2, Up regulated genes		fold
TACSTD1	tumor-associated calcium signal transducer 1	12.42
EPCAM	epithelial cell adhesion molecule	9.81
SPINT2	serine peptidase inhibitor, Kunitz type, 2	9.24
S100A4	S100 calcium binding protein A4	8.67
TACSTD2	tumor-associated calcium signal transducer 2	7.71
CDH1	cadherin 1, type 1, E-cadherin	6.49
MAL	mal, T-cell differentiation protein	6.16
ZIC2	Zinc family member 2 (Drosophila)	6.03
C10orf58	chromosome 10 open reading frame 58	5.98
MAL2	mal, T-cell differentiation protein 2	5.94
S100A4	S100 calcium binding protein A4	5.93
UGT2B7	UDP glucuronosyltransferase 2 family, polypeptide B7	5.72
APOE	apolipoprotein E	5.71
SPP1	secreted phosphoprotein 1	5.70
GLDC	glycine dehydrogenase	5.48
SPP1	secreted phosphoprotein 1	5.44
UCP2	uncoupling protein 2 (mitochondrial, proton carrier)	5.39
MDK	midkine (neurite growth-promoting factor 2)	5.39
ALDH1A1	aldehyde dehydrogenase 1 family, member A1	5.13
FAM84B	family with sequence similarity 84, member B	5.08

Cluster #2, Down regulated genes		fold
TMEM98	transmembrane protein 98	0.07
EFEMP1	EGF-containing fibulin-like extracellular matrix protein 1	0.07
DCN	decorin (DCN), transcript variant C	0.07
CDH11	cadherin 11, type 2, OB-cadherin (osteoblast)	0.09
MT1E	metallothionein 1E (MT1E)	0.10
COL3A1	collagen, type III, alpha 1 (COL3A1)	0.10
ALDH1A3	aldehyde dehydrogenase 1 family, member A3	0.10
IGFBP4	insulin-like growth factor binding protein 4	0.11
COL5A2	collagen, type V, alpha 2	0.11
PDPN	podoplanin (PDPN)	0.12
PLOD2	procollagen-lysine, 2-oxoglutarate 5-dioxygenase 2	0.12
SPOCK1	sparc/osteonectin, (testican) 1	0.12
FLNC	filamin C, gamma (actin binding protein 280)	0.12
CRISPLD2	cysteine-rich secretory protein LCCL domain containing 2	0.14
DKK3	dickkopf homolog 3 (Xenopus laevis) (	0.14
RAC2	rho family, small GTP binding protein Rac2	0.14
SGK1	serum/glucocorticoid regulated kinase 1	0.15
DPYSL3	dihydropyrimidinase-like 3	0.15
SGK1	serum/glucocorticoid regulated kinase 1	0.15
PLIN2	perilipin 2	0.15

Supplementary Table 10

The 25 pathways that are most significantly upregulated in OCI Cluster 1 cell lines

	PATHWAYS UPREGULATED IN CLUSTER 1 Ingenuity Canonical Pathways	p-value	Molecules
1	Hepatic Fibrosis / Hepatic Stellate Cell Activation	0.000002	IGFBP4,VCAM1,CTGF,TNFRSF1A,FGF2,ACTA2,BAMBI,VEGFC,IGFBP5,IL1R1,MYL9,TGFBR2,COL1A2,COL1A1,CCL2,TIMP1,COL3A1
2	Inhibition of Matrix Metalloproteases	0.0002	TIMP4,MMP23B,TIMP1,RECK,THBS2,TFPI2,LRP1
3	Integrin Signaling	0.0002	RAC2,PARVA,RALA,TSPAN5,ASAP1,DIRAS3,ACTA2,ITGA5,TLN1,MYLK,MYL9,RRAS2,RND3,RHOU,CAV1,ZYX,CAPN2
4	Chondroitin and Dermatan Biosynthesis	0.0006	CHSY3,CHPF,CSGALNACT1
5	HMGB1 Signaling	0.0009	VCAM1,RRAS2,CCL2,RND3,TNFRSF1A,DIRAS3,RHOU,IL1R1,SERPINE1,PLAT
6	Germ Cell-Sertoli Cell Junction Signaling	0.0016	TGFBR2,RAC2,CDH2,RRAS2,RND3,TUBB6,TNFRSF1A,DIRAS3,TUBB2A,ACTA2,RHOU,ZYX,RAB8B
7	ILK Signaling	0.002	PARVA,TNFRSF1A,DIRAS3,ACTA2,VEGFC,HIF1A,MYL9,TGFB1I1,RND3,FLNC,SNAI2,RHOU,IRS2,PTGS2
8	IL-8 Signaling	0.003	RAC2,VCAM1,DIRAS3,VEGFC,MAP4K4,IRAK3,GNAI2,MYL9,GNB4,RRAS2,RND3,RHOU,GNG2,PTGS2
9	Ascorbate Recycling (Cytosolic)	0.003	GLRX,GSTO1
10	RhoGDI Signaling	0.004	DIRAS3,ACTA2,ITGA5,CDH11,GNAI2,MYL9,GNB4,CDH2,RND3,PIP5K1C,RHOU,GNG2,ARHGEF3
11	Regulation of Actin-based Motility by Rho	0.005	MYLK,MYL9,RAC2,RND3,PIP5K1C,DIRAS3,ACTA2,RHOU
12	Gαi Signaling	0.006	GNAI2,S1PR3,GNB4,RRAS2,RALA,LPAR1,CAV1,RGS4,GNG2,FPR1
13	Epithelial Adherens Junction Signaling	0.007	MYL9,TGFBR2,CDH2,NOTCH2,RRAS2,TUBB6,SNAI2,ACTA2,TUBB2A,ACVR1,ZYX
14	Glioma Invasiveness Signaling	0.010	TIMP4,RRAS2,RND3,TIMP1,DIRAS3,RHOU
15	IGF-1 Signaling	0.014	IGFBP4,IGFBP6,NEDD4,CTGF,RRAS2,IGFBP5,IRS2,CYR61
16	UDP-N-acetyl-D-glucosamine Biosynthesis II	0.014	UAP1,GFPT2
17	Glycogen Biosynthesis II (from UDP-D-Glucose)	0.014	UGP2,GBE1
18	Signaling by Rho Family GTPases	0.017	DIRAS3,ACTA2,ITGA5,CDH11,GNAI2,MYLK,MYL9,GNB4,CDH2,RND3,PIP5K1C,RHOU,GNG2,ARHGEF3
19	NF-κB Signaling	0.018	IL33,TGFBR2,GHR,RRAS2,TNFRSF1A,TNFAIP3,MAP4K4,IL1R1,IRAK3,CARD11,TNFSF13B
20	Complement System	0.019	SERPING1,CD59,C1S,CFI
21	Colorectal Cancer Metastasis Signaling	0.020	TGFBR2,GNB4,RRAS2,MMP23B,RND3,TNFRSF1A,DIRAS3,RHOU,VEGFC,GNG2,PTGS2,PTGER2,LRP1,WNT5A
22	Remodeling of Epithelial Adherens Junctions	0.020	RALA,TUBB6,ACTA2,TUBB2A,ZYX,MAPRE3
23	Sphingosine-1-phosphate Signaling	0.025	GNAI2,S1PR3,RND3,DIRAS3,CASP1,RHOU,CASP4,SMPD1
24	Virus Entry via Endocytic Pathways	0.026	RAC2,RRAS2,ITSN1,FLNC,ACTA2,CAV1,ITGA5
25	CXCR4 Signaling	0.026	GNAI2,MYL9,GNB4,RRAS2,RND3,DIRAS3,CXCL12,RHOU,ITPR1,GNG2

Supplementary Table 11

The 25 pathways that are most significantly upregulated in OCI Cluster 2 cell lines

	PATHWAYS UPREGULATED IN CLUSTER 2 Ingenuity Canonical Pathways	p-value	Molecules
1	Cell Cycle Control of Chromosomal Replication	0.000000	MCM3,MCM6,MCM2,CDT1,CDK4,ORC6,MCM4,CDK2,MCM7
2	Estrogen-mediated S-phase Entry	0.000001	CCNE1,CDK4,E2F5,CDK1,E2F2,CDK2
3	Cell Cycle Regulation by BTG Family Proteins	0.000007	PRMT1,CCNE1,CDK4,E2F5,E2F2,CDK2
4	Role of BRCA1 in DNA Damage Response	0.000017	FANCD2,FANCG,E2F5,RBBP8,RFC5,E2F2,RFC3
5	Cyclins and Cell Cycle Regulation	0.000089	CCNE1,CDK4,WEE1,E2F5,CDK1,E2F2,CDK2
6	CHK Proteins in Cell Cycle Checkpoint Control	0.000105	E2F5,RFC5,CDK1,E2F2,CDK2,RFC3
7	GADD45 Signaling	0.000115	CCNE1,CDK4,CDK1,CDK2
8	Superpathway of Cholesterol Biosynthesis	0.000479	ACAT2,TM7SF2,HMGCS1,LBR
9	Hereditary Breast Cancer Signaling	0.000912	FANCD2,FANCG,CDK4,WEE1,RFC5,CDK1,RFC3
10	Myo-inositol Biosynthesis	0.001122	ISYNA1,IMPA2
11	Cell Cycle: G1/S Checkpoint Regulation	0.001288	CCNE1,CDK4,E2F5,E2F2,CDK2
12	Pyridoxal 5'-phosphate Salvage Pathway	0.001622	NEK2,CDK4,TTK,CDK1,CDK2
13	dTMP De Novo Biosynthesis	0.001820	TYMS,SHMT1
14	DNA damage-induced 14-3-3σ Signaling	0.002138	CCNE1,CDK1,CDK2
15	Zymosterol Biosynthesis	0.002754	TM7SF2,LBR
16	G2/M DNA Damage Checkpoint Regulation	0.002818	WEE1,CKS1B,TOP2A,CDK1
17	Glioblastoma Multiforme Signaling	0.003981	CCNE1,PLCG2,CDK4,FZD3,E2F5,E2F2,CDK2
18	Breast Cancer Regulation by Stathmin1	0.004786	STMN1,CCNE1,ARHGEF16,E2F5,PPP1R14A,CDK1,E2F2,CDK2
19	Salvage Pathways of Pyrimidine Ribonucleotides	0.006026	NEK2,CDK4,TTK,CDK1,CDK2
20	Folate Transformations I	0.006310	MTHFD2,SHMT1
21	Regulation of Cellular Mechanics by Calpain Protease	0.006918	CCNE1,CDK4,CDK1,CDK2
22	Glutaryl-CoA Degradation	0.007943	ACAT2,HSD17B8
23	Ketogenesis	0.007943	ACAT2,HMGCS1
24	ATM Signaling	0.008710	FANCD2,CBX5,CDK1,CDK2
25	Mevalonate Pathway I	0.011482	ACAT2,HMGCS1

See Supplementary Data 3 for the full list of IPA enriched pathways.

Supplementary Table 12

The clustering order, passage number, and confluency of OCI cell lines profiled in the RPPA heatmap in Supplementary figure 12. Clear Cell (light blue), Serous (dark green), Endometrioid (pink), Mucinous (light green) other histotypes (yellow), cluster 1 (dark blue) cluster 2 (red). See online Excel data files 4 and 6 for detailed information.

RPPA Cluster Order		Cluster	Exp. #	RPPA Passage #	Density	Histotype
1	OCI-C3x	2	2	**10**		Clear Cell
2	OCI-C3x	2	2	**10**	Conf.	Clear Cell
3	OCI-C3x	2	1	**9**		Clear Cell
4	FCI-P2p	2	1	**14**		Papillary Serous
5	FCI-P2p	2	2	**15**		Papillary Serous
6	FCI-P2p	2	2	**15**		Papillary Serous
7	FCI-P2p	2	2	**15**		Papillary Serous
8	OCI-C5x	2	2	**15**		Clear Cell
9	OCI-C5x	2	2	**15**		Clear Cell
10	OCI-C5x	2	2	**15**		Clear Cell
11	OCI-C5x	2	1	**14**		Clear Cell
12	OCI-E1p	2	2	**12**		Endometrioid
13	OCI-E1p	2	2	**12**		Endometrioid
14	OCI-E1p	2	2	**12**		Endometrioid
15	OCI-E1p	2	1	**13**		Endometrioid
16	OCI-C4p	2	2	**12**		Clear Cell
17	OCI-C4p	2	2	**12**		Clear Cell
18	OCI-C4p	2	1	**11**		Clear Cell
19	OCI-U1a	2	2	**14**		Mullerian
20	OCI-U1a	2	2	**14**		
21	OCI-U1a	2	2	**14**		
22	OCI-U1a	2	1	**13**		
23	OCI-P5x	2	2	**10**		Papillary Serous
24	OCI-P5x	2	2	**10**		Papillary Serous
25	OCI-P5x	2	2	**10**		Papillary Serous
26	OCI-P5x	2	1	**9**		Papillary Serous
27	OCI-P8p	2	2	**22**		Papillary Serous
28	OCI-EP1p	2	1	**13**		Endometrioid
29	OCI-C1p	1	2	**16**		Clear Cell
30	OCI-C1p	1	2	**16**		Clear Cell
31	OCI-C1p	1	2	**16**		Clear Cell
32	OCI-C1p	1	1	**15**		Clear Cell
33	OCI-C2p	1	1	**4**		Clear Cell
34	OCI-E2p	1	2	**13**		Endometrioid
35	OCI-E2p	1	2	**13**		Endometrioid
36	OCI-E3x	1	2	**16**	Conf.	Endometrioid
37	OCI-E3x	1	2	**16**		Endometrioid
38	OCI-E3x	1	2	**16**		Endometrioid
39	OCI-E3x	1	1	**15**		Endometrioid
40	OCI-P3a	1	2	**13**		Papillary Serous
41	OCI-P3a	1	2	**13**		Papillary Serous
42	OCI-P3a	1	2	**13**		Papillary Serous
43	OCI-P4p	1	1	**11**		Papillary Serous
44	OCI-D1p	1	1	**13**		Dysgerminoma
45	OCI-D1p	1	2	**14**		Dysgerminoma
46	OCI-D1p	1	2	**14**		Dysgerminoma
47	OCI-D1p	1	2	**14**		Dysgerminoma

RPPA Cluster Order		Cluster	Exp. #	RPPA Passage	Density	Histotype
48	OCI-EP1p	1	2	**17**		Endometrioid
49	OCI-EP1p	1	2	**17**		Endometrioid
50	OCI-EP1p	1	2	**14**		Endometrioid
51	OCI-M1p	1	1	**13**		Mucinous
52	OCI-C2p	1	2	**5**		Clear Cell
53	OCI-C2p	1	2	**5**		Clear Cell
54	OCI-P9a1	1	1	**17**		Papillary Serous
55	OCI-CSp	1	1	**20**		Carcinosarcoma
56	FCI-P1p	1	2	**13**	Conf.	Papillary Serous
57	FCI-P1p	1	2	**13**		Papillary Serous
58	OCI-P4p	1	1	**11**		Papillary Serous
59	FCI-P1p	1	2	**12**		Papillary Serous
60	FCI-P1p	1	2	**12**		Papillary Serous
61	OCI-P4p	1	1	**11**		Papillary Serous
62	OCI-CSp	1	2	**21**		Carcinosarcoma
63	OCI-CSp	1	2	**21**		Carcinosarcoma
64	OCI-CSp	1	2	**21**		Carcinosarcoma
65	OCI-P6p	1	2	**14**		Papillary Serous
66	OCI-P6p	1	2	**14**		Papillary Serous
67	OCI-P6p	1	2	**14**		Papillary Serous
68	OCI-P6p	1	2	**14**		Papillary Serous
69	OCI-P1a	1	1	**13**		Papillary Serous
70	FCI-P1p	1	1	**12**		Papillary Serous
71	OCI-P8p	1	1	**15**	Conf.	Papillary Serous
72	OCI-P6p	1	1	**13**		Papillary Serous
73	OCI-P9a1	1	1	**17**		Papillary Serous
74	OCI-P2a	1	1	**7**		Papillary Serous
75	OCI-P3a	1	1	**13**		Papillary Serous
76	OCI-P7a	1	1	**18**		Papillary Serous
77	OCI-M1p	1	2	**14**		Mucinous
78	OCI-M1p	1	2	**14**		Mucinous
79	OCI-M1p	1	2	**14**		Mucinous
80	OCI-P2a	1	2	**8**		Papillary Serous
81	OCI-P2a	1	2	**8**		Papillary Serous
82	OCI-P2a	1	2	**8**		Papillary Serous
83	OCI-P8p	1	2	**22**		Papillary Serous
84	OCI-P1a	1	2	**14**	Conf.	Papillary Serous
85	OCI-P9a1	1	2	**18**		Papillary Serous
86	OCI-M1p	1	2	**14**		Mucinous
87	OCI-P1a	1	2	**14**		Papillary Serous
88	OCI-P1a	1	2	**14**		Papillary Serous
89	OCI-P9a2	1	2	**17**		Papillary Serous
90	OCI-P9a2	1	2	**14**		Papillary Serous
91	OCI-P9a2	1	2	**14**		Papillary Serous
92	OCI-P9a2	1	2	**14**		Papillary Serous
93	OCI-P7a	1	2	**18**		Papillary Serous
94	OCI-P7a	1	2	**18**		Papillary Serous
95	OCI-P7a	1	2	**18**		Papillary Serous

Supplementary Table 13

OCI and SOC cell lines have a reproducible clustering pattern

The OCI and SOC cell lines form similar clusters regardless of the presence or absence of patient samples in the mRNA data analysis and whether the analysis was done using mRNA or protein expression levels.

	mRNA Cluster 1	mRNA Cluster 2	RPPA Cluster 1	RPPA Cluster 2	Patient Cluster 1	Patient Cluster 2
1	FCI-P1p	A2780	FCI-P1p	A2780	FCI-P1p	A2780
2	OCI-C1p	OV90	OCI-C1p	OV90	OCI-C1p	OV90
3	OCI-C2p	RMG-1	OCI-C2p	RMG-1	OCI-C2p	RMG-1
4	OCI-CSp	RMUGL	OCI-CSp	RMUGL	OCI-CSp	RMUGL
5	OCI-D1p	SKOV3	OCI-D1p	SKOV3	OCI-D1p	SKOV3
6	OCI-E2p	TOV112D	OCI-E2p	TOV112D	OCI-E2p	TOV112D
7	OCI-E3x	FCI-P2p	OCI-E3x	FCI-P2p	OCI-E3x	FCI-P2p
8	OCI-EP1p	OCI-C3x	OCI-EP1p	OCI-C3x	OCI-EP1p	OCI-C3x
9	OCI-M1p	OCI-C5x	OCI-M1p	OCI-C5x	OCI-M1p	OCI-C5x
10	OCIP1a	OCI-E1p	OCIP1a	OCI-E1p	OCIP1a	OCI-E1p
11	OCI-P2a	OCI-P5x	OCI-P2a	OCI-P5x	OCI-P2a	OCI-P5x
12	OCI-P3a	OCI-U1a	OCI-P3a	OCI-U1a	OCI-P3a	OCI-U1a
13	OCI-P4p		OCI-P4p	**OCI-C4p**	OCI-P4p	**OCI-C4p**
14	OCI-P6p		OCI-P6p		OCI-P6p	
15	OCI-P7a		OCI-P7a		OCI-P7a	
16	OCI-P8p		OCI-P8p		OCI-P8p	
17	OCI-P9a1		OCI-P9a1		OCI-P9a1	
18	OCI-P9a2		OCI-P9a2		OCI-P9a2	
	**OCI-C4p**					

Supplementary Table 14

OCI cell lines have a stable phenotype

We confirmed the drug response experiments with equal number of serous, clear cell and endometrioid subtypes in each group i.e.; cluster 1 vs. 2 (see Supplementary Fig. 20 b). This result demonstrates that that the differences between cluster 1 vs. cluster 2 cell lines are not due to histotype differences. Furthermore, there were at least 5 to 36 passages between the RPPA analysis and Taxol/Cisplatin sensitivity experiments. Nevertheless, there was a close concordance between RPPA clusters and drug response, providing further evidence that the phenotype of OCI lines are stable over many passage in culture.

	Cell Line	Cluster	mRNA/RPPA Passage	Drug Assay Passage	Histotype
1	FCI-P1p	1	12	17	Papillary Serous
2	OCI-P7a	1	18	23	Papillary Serous
3	OCI-C1p	1	15	19	Clear Cell
4	OCI-C2p	1	5	19	Clear Cell
5	OCI-E3x	1	15	21	Endometrioid
5	FCI-P2p	2	14	20	Papillary Serous
7	OCI-P5x	2	9	40	Papillary Serous
8	OCI-C3x	2	9	19	Clear Cell
9	OCI-C5x	2	14	51	Clear Cell
10	OCI-E1p	2	12	17	Endometrioid

Supplementary Table 15

List of proteins that are differentially expressed that are differentially expressed between cluster 1 vs. cluster 2 cell lines, corresponding to the RPPA heatmap in Figure 7B. The genes in italic in cluster 1 have been previously implicated in Taxol resistance. See Supplementary Table 16, and online Excel data files 4 and 7 for detailed information.

	Over-expressed in Cluster 1	Over-expressed in Cluster 2
1	***a.Tubulin***	ACC_pS79
2	***ab_Crystalline***	AIB1
3	***AKT***	AMPKa
4	***AR.C19***	AR.N20
5	BAD_pS112	AR.N20
6	BOP1.N16	ATRIP
7	BRCA2	BCl.XL
8	***c.JUN_pS73***	BIM.V
9	Caveolin.1	c.Myc_pT58
10	CD20	CASK
11	Cofilin	Caspase.7.cleaved
12	Cofilin_pS3	CHK2
13	Collagen.VI	CHK2_pT68
14	COX2	Cyclin.B1
15	eIF4E	Cyclin.B1
16	***Erg.1_2_3***	Cyclin.E
17	***FAK_pY397***	Cyclin.E1
18	Fibronectin	Cyclin.E2
19	***FOXO3a***	ERa_pS118
20	HSP27	HSP70
21	***MAPK_pT202***	JUNB
22	***MAPK_pT202***	LCK
23	***N.Cadherin***	MKLP.1.D17
24	p21	NBS1
25	***PAI.1***	p53
26	***PAX2***	p90RSK
27	PKCa	p90RSK_pT359
28	***PKCa_pS657***	p90RSK_pT359
29	***PTEN***	PKCa
30	***PTEN.138G50***	PLK1
31	S6_pS235	SMAC_DIABO
32	***SMAD3***	***SRC_pY527***
33	***SMAD3_pS423***	Stathmin
34	TSC2_pT1462	Telomerase
35	YBI	XRCC1
36		ZNF342

Supplementary Table 16

List of proteins with known Taxol resistance association that are differentially expressed between Cluster 1 vs. Cluster 2 OCI cell lines in RPPA analysis.

RPPA Probe	Association with Taxol Resistance
**a.Tubulin**	Tubulin is over-expressed and/or mutated in Taxol resistant cells [9-12]
**AKT**	Rapamycin with paclitaxel displayed synergistic effects [13,14]. Constitutively active AKT contributes to Vincristine resistance [15]. AKT induces survival in paclitaxel treated cells [16]. Akt directly regulates the transcriptional activity of c-Jun [17]
**AR.C19.**	Androgen receptor is activated by STAT3 [18].
**c.JUN pS73**	Paclitaxel-resistant human ovariancCancer cells undergo c-Jun NH2-terminalkKinase-mediated apoptosis [19]. A physical interaction of Stat3 with c-Jun has been reported both in vitro and in vivo. Stat3 and c-Jun cooperated to yield maximal enhancer function, point mutations of Stat3 within the interacting domains blocked both physical interaction of Stat3 with c-Jun and their cooperation [20]
**Crystalline**	Crystalline interact with tubulin to regulate the equilibrium between tubulin and microtubules [21].
**Erg.1_2_3**	Expression of EGR-1/p38MAPK plays a critical role in paclitaxel resistance of ovarian carcinoma cells [22]
**FAK _pY397**	Docetaxel induces FAK cleavage in taxane-sensitive ovarian cancer cells but not in resistant cells [23].
**FOXO3a**	ERK promotes tumorigenesis by inhibiting FOXO3a [24].
**MAPK_ pT202**	MEK inhibitor CI-1040 potentiates efficacy of Taxol in xenograft tumor modes [25]. RNAi screening identified Erk1 as enhancing paclitaxel activity [26]. Inactivation of ERK is necessary for the enhancement of paclitaxel cytotoxicity by U0126 [25].
**N.Cadherin**	N-Cadherin is over-expressed in Taxol resistant cells [27].
**PAI.1**	MEK/ERK1/2 and SMAD3 are essential for PAI-1 induction initiated by microtubule disruption [28].
**PAX2**	PAX2 expression correlated with resistance against apoptosis and proinvasive phenotype [29].
**PKCa**	Purified protein kinase C phosphorylates microtubule-associated protein 2 [30].
**PTEN**	Silencing AKT in PTEN-mutated prostate cancer cells enhances the antitumor effects of taxol [31].
**SMAD3**	Increased expression in Paclitaxel resistant cells [32], SMAD3 binds to microtubules[33]. SMAD3 and SMAD4 cooperate with c-Jun/c-Fos to mediated transcription [34].
**SRC**	Knockdown of Src enhanced paclitaxel-mediated growth inhibition in ovarian cancer cells [35,36], SRC activates STAT3 [37]. STAT3 siRNA inhibited Bcl-2 expression [38]. Bcl-2 Down-Regulation is associated with Paclitaxel resistance [39]. Constitutive activation of Stat3 by the Src causes growth of breast carcinoma cells [40].
**STAT3**	STAT3 activation through Src leads to Taxol resistance [41], STAT3 is activated by ERK1 and induces AKT. STAT3 binds the C-terminal tubulin [42]. Knockdown of Stat3 reduces AKT1 expression [43] STAT3 is induced by Src [44].

The specific mRNA and protein expression differences cluster 1 vs. 2 are difficult to associate with specific genes associated with Cisplatin resistance, which can be due to the multitude of complex mechanisms associated with [45]: **(1)** ATP-Binding Cassette Transporters, **(2)** Copper Transporter 1, **(3)** Transmembrane Protein 205, **(4)** Glucose Transporter 1, **(5)** Heat Shock Proteins, **(6)** Small GTPases, **(7)** Ribosomal Proteins, **(8)** DNA Methylation, **(9)** Histone Modification, **(10)** Nuclear Factor (Erythroid-Derived 2)-Like 2, **(11)** GC-Binding Factor 2, **(12)** Epithelial to Mesenchymal Transition, **(13)** Wingless Gene, **(14)** Protein Kinase B, **(15)** MicroRNA changes and **(16)** DNA Repair and BRCA Mutant Genes [45]

SUPPLEMENTARY NOTE 1

Cell Culture Medium

Several different media have been previously used to culture human breast and ovarian cells including RPMI, DMEM, Ham's F12, MCDB-105, McCoy's 5A, and MCDB-170 (MEGM). In general only a small percent of primary ovarian or breast cancer samples can be established as cell lines using these standard cell culture media. Consistent with this, we failed to establish any permanent human breast or ovarian cancer cell lines using these media to culture cells from more than one hundred tumors. Therefore, we explored the use of a cell culture media that we had previously described in Ince *et al,*. [46].

The chemically-defined cell culture medium we developed can support long-term growth of normal and transformed human breast cells without undefined components such as serum, feeder-layers, tissue extracts or pharmacological reagents [46]. Using a version of this medium optimized for normal cells (WIT-P), we were able to culture human breast epithelial cells (BPEC) for more than seventy population doublings during six months of continuous culture, a nearly 10^{21}-fold expansion of cell number [46]. In contrast, in standard medium these normal breast epithelial cells ceased growing after several passages [46]. Importantly, it was recently shown that normal breast cells maintain high telomerase activity in culture in this medium [47], which is not the case in standard media.

We initially tested a version of the medium (WIT-T) optimized for transformed human breast cells (BPLER) [46] to establish human ovarian tumor cell lines, but were unsuccessful with more than a dozen tumor samples.

Next, we examined whether modifications in the concentration of distinct components of the media would support the growth of primary ovarian tumor cells. First, we reasoned that low levels of serum may be required to mimic the physiologic environment of normal ovary, fallopian tube and ovarian cancers in the peritoneal cavity.

Since most normal epithelia are not in direct contact with blood or serum, we had used a chemically-defined serum free medium to culture normal breast epithelial cells. However, the normal ovaries and fallopian tubes are normally surrounded by normal peritoneal fluid, which contains protein concentrations that are similar to serum levels, which is increased to above plasma levels in malignant ascites fluid associated with ovarian cancer [48-50]. Consistent with these observations, the addition of serum to the medium proved to be necessary, but not sufficient for growth of ovarian tumor cells; additional factors had to be optimized (**Supplementary Fig. 1-2**).

One of the difficulties associated with optimizing media is the non-obvious and synergistic combinatorial effects of individual components. The individual additives increased ovarian cancer culture success only incrementally in many cases. However, cell proliferation increased exponentially when all components were added at optimal concentrations (**Supplementary Fig. 1-4**). As a result, after many years of optimization, we found that inclusion of insulin, hydrocortisone, EGF, and cholera toxin in addition to fetal bovine serum to media showed broad efficacy in supporting the growth of the serous ovarian cancers.

The non-serous ovarian tumor subtypes, such as endometrioid and mucinous cancers, express estrogen receptor (ER). In this case we found that addition of β-Estradiol (E2) is necessary to enhance the growth of these tumors subtypes (**Supplementary Fig. 2**).

We also had to optimize O_2 levels because while many papillary serous and clear cell subtypes proliferate best in ambient O_2 (18 to 21%), the endometrioid and mucinous subtypes proliferate best at low O_2 levels (5 to 10%) in general (see Supplemental Table 3 for details). Interestingly, we found that the O_2 levels that are commonly used for low O_2 cultures(1 to 2 % O_2) is detrimental for ovarian carcinoma cells (data not shown). Intriguingly, we found that culturing ER+ tumors at lower O_2 levels (5%) is necessary to maintain long-term ER expression *in vitro*. It has been shown that estrogens can play a role as pro-oxidants or anti-oxidants depending on the cell types[51]. This might be one reason ER+ endometrioid and mucinous OCI lines in 100nM β-Estradiol maintain their phenotype best in low O_2 levels.

A very time consuming aspect of the medium optimization process was the need to validate applicability of the medium and methods across the broad spectrum of specimens and cancer subtypes. We found that while some ingredients have little effect on culture efficiency for some samples, they are absolutely essential for others (**Supplementary Fig. 3**). Importantly, the effects of removing these components become more apparent after multiple passages (**Supplementary Fig. 4**). Hence, effects of each ingredient has to be tested over many passages.

The cell attachment surface is also important; the standard tissue culture plates have a uniformly negatively charged surface which was not suitable for primary ovarian cells and produce variable results. In contrast we use a modified cell culture plastic with mixed positive and negative charges (Primaria, BD), which greatly helps in preserving cell morphology and heterogeneity of the original tumor. We found that while some cell

lines proliferate best on standard plates others require Primaria plates (**Supplementary Table 3**).

All of these factors - the non-obvious nature of combinatorial outcomes, all-or-none synergistic effects, the need to test conditions in multiple passages and in multiple cell lines, and the very large number of conditions to test - precluded an incremental approach to medium development.

With the current formulation of OCMI, we were able to culture ovarian tumors with relatively high efficiency (>95%, 25 out of 26). At this point we did not go forward with further experiments to determine which medium components are responsible for improved success compared to standard media. This decision was influenced by the fact that the baseline media contains 85 components [46], many of which are distinct from standard cell culture media that are generally comprised of 30 to 55 ingredients; including **DMEM** (32 components, Life Technologies, Cat # 31600), **Ham's F-12** (*47 components*, Sigma Cat# 51651C), **DMEM/F12** (*52 components*, Sigma Cat# 51445C), **RPMI-1640** (*40 components*, Sigma Cat # R0883,), **McCoys's 5A** (*45 components*, Sigma, Cat # M4892,), **MCDB105** (*53 components*, Sigma, Cat # M6395), **MCDB-170** (*53 components*, US Biological Cat# M2162).

It is difficult to determine whether all of the OCMI components are necessary for its performance. Leaving aside the undefined ingredients such as serum or tissue extracts, and just testing 30-40 components in WIT that are generally absent in standard media would require examining more than 1.0 x 10 [9-12] different conditions, which is well beyond the scope of this manuscript or capability of an academic laboratory. For this

reason the initial invention of the WIT medium was the result of a theoretical deduction about the metabolic pathways necessary to maintain healthy cells in culture and not through a trial-and-error approach. Since we have not explored all the $1.0 \times 10^{9-12}$ different variations, it is possible that additional refinements in the medium formulation in the future can further improve the successful culture of human cells.

Likewise, even after excluding the 30 to 40 differences between WIT vs. standard media in order to concentrate on the seven differences between WIT vs. OCMI, and testing each variable at three concentrations would require examining 262,144 combinations. Therefore, it was not possible to systematically test each of these variations even in retrospect.

Despite these daunting odds, our empiric efforts led to identification of a combination of insulin, hydrocortisone, EGF, cholera toxin, serum, β-Estradiol, O_2 and cell culture flasks that supported long term culture of a majority of primary ovarian cancers.

The medium optimized for human ovarian tumors, named OCMI (-e when β-Estradiol added), contains final concentrations of EGF (0.01 ug/mL, E9644, Sigma-Aldrich, St. Louis, MO), Insulin (20 ug/mL, I0516, Sigma-Aldrich), Hydrocortisone (0.5 ug/mL, H0888, Sigma-Aldrich), 25ng/mL Cholera Toxin (227035, Calbiochem, EMD Millipore, Billerica, MA) together with 2 - 5 % heat inactivated fetal bovine serum (HyClone, Thermo Fisher Scientific, Waltham, MA) (**Supplementary Fig. 1-2**)

To our knowledge, none of the standard media support the culture of all of the existing SOC lines. Thus, it has been difficult to compare a large panel of SOC lines with each other. We also found that none of the OCI lines can be cultured in existing standard

media **(Supplementary Fig. 5 A-D**). In contrast, all of the SOC lines we tested can be cultured in OCMI medium (**Supplementary Fig. 5 E=H**). Our results indicate that OCMI medium can serve as a universal culture medium for SOC lines facilitating their comparisons with each other.

Watanabe *et al.,* used a DMEM:F12 based medium supplemented with apoptosis inhibitor (Y-27632) that inactivates Rho-associated kinase (ROCK) to culture human ES cells [1]. Liu et al., used this medium to culture human tumors [2]. However, they reported establishing only one normal ovarian cell line, and no ovarian cancer cell lines. We were unable to use this medium to establish ovarian carcinoma cell lines because we found that that Y-27632 inhibits proliferation of OCI lines (**Supplementary Fig. 6)**. Consistent with our experience, others also recently reported that Y-27632 negatively impacts culture of hematopoietic progenitor cells, human adipose-derived stem cells and melanomas [2]. Thus, this is not an approach that can be used universally on all cells.

Sato et al. also reported a DMEM:F12 based medium for the culture of human small intestine, Barrett's esophagus and colon epithelium using drugs that inhibit of Alk and p38 [52]. In the absence of these two kinase inhibitors, human intestinal cultures were growth arrested after 10 to 20 population doublings [52]. It is a significant concern that cell lines derived using the Liu or Sato et al., media will be ‘addicted’ to these drugs. In addition, the presence of feeder layers causes difficulties with the interpretation of cytotoxic assays since the observed results can be due to direct effects on cancer cells as well as indirect effects on the feeder layer. In contrast, we have no such drugs or feeder layers in our system, which is a significant improvement in new culture methods.

Tumor Tissue Collection and Clinical Information

All study procedures were approved by the Internal Review Boards of the Brigham and Women's Hospital and the University of Miami to collect discarded tissues with a written consent from all patients. In this initial study we concentrated on developing methods for successful culture of human ovarian tumors. For this purpose we used anonymized discarded human tissue and did not have access to clinical patient follow up information retrospectively. A prospective study with larger number of patients will be needed to examine the direct comparison of individual patients to treatment and in vitro response of their corresponding cell line, which is underway.

Establishment of Cell Lines

Tumors are complex tissues composed of many cell types including stromal cells such as fibroblasts, endothelial cells, leukocytes, macrophages as well as normal epithelial cells that are intermingled with tumor cells [53]. Among these, fibroblasts have historically been the easiest cells to grow in standard culture medium [54,55]. In general serum promotes fibroblast growth and inhibits epithelial cell proliferation. When tumor tissue is cultured in medium with high serum content, typically there is an exponential growth of fibroblasts such that in a few weeks the fibroblasts completely overtake the culture plate, and soon all other cell types including tumor cells are eliminated. For this reason we used low levels of serum (2 %) to culture ovarian tumor cells during the initial passages (1-5) to suppress fibroblast growth.

Another difference between epithelial cell and fibroblasts is adherence to tissue culture plastic; in general epithelial cells are more strongly adherent to the culture flasks and require higher concentrations of trypsin to release them. Thus, it is possible to treat the

plates with diluted trypsin first (0.05%), which selectively removes stromal cells. Afterwards, the epithelial cells that are still attached to the culture plate can be were treated with 0.25% trypsin for sub-culturing. OCMI was designed to support epithelial tumor cell proliferation and suppress fibroblast growth. However, in general it takes 4-6 passages with differential trypsinization to establish tumor cultures free of stromal and normal cell types. Afterwards the FBS levels were increased to 5% to increase tumor cell proliferation.

We observed a remarkable degree for consistency in the phenotype of OCI lines in long term culture. All OCI cell lines we cultured for at least 20-25 population doublings. In several cases, we carried out a formal population doubling analysis (**Figure 1**), which demonstrated that the OCI lines proliferate for at least 120 days (~60 population doublings). Importantly, there was a remarkable correlation among our results; even though the mRNA profiling (**Figure 3**), protein profiling (**Figure 4**) and drug sensitivity (**Figure 7**) experiments were carried out by different investigators at different times (passages 8-56) (**Supplementary Fig. 12-13, Supplementary Table 12-14**). These results indicate that OCI cell lines have a stable and reproducible phenotype.

Clonal Selection

Mindful of the possibility of clonal selection, we carefully monitored all OCI cultures for emergence of fast growing colonies, eliminated plates with too few starting cells and avoided partial trypsinization of plates during sub-culturing of OCI lines.

Measures of cell proliferation

In many previous reports, the cumulative number of cell passages has been used to indicate successful establishment of cell lines. However, it is important to note that the

number of passages is not adequate by itself to verify net increase in tumor cell numbers. The cell passage number refers to the number of times the cells are successfully lifted from one plate and seeded into a new culture plate. This indicates that at least some of the cells can tolerate the transfer and are still alive. However, passage number does not necessarily correlate with increased cell numbers. For example, we were able to passage the tumor cell line OCI-C5x in MCDB-105/M199 for nearly 20 passages. However, the population doubling curve of these cells stayed flat after 7 passages. Thus, there was no net increase in cell numbers between passages 7 and 20 (**Figure 1 d**). Hence these cells, when grown using MCDB-105/199, could not provide a practical platform to carry out many experiments.

The utility of a culture system as a practical and robust experimental platform is better assessed by measuring population doublings. An objective comparison of results from different studies can be made with previous work in terms of 'population doublings', or the log(2) of the number of cells harvested less the log (2) of the number of cells seeded; hence 2 cells expand to 1,024 cells in 10 population doublings (2^{10}= 1,024). Each 10 population doublings is approximately equal to 3 orders of magnitude ($\times 10^3$) net increase in cell numbers, and so 20 population doublings would be close to a 1 million-fold increase and 30 population doublings would be close to a 1 billion fold increase in net cell numbers. We have achieved 30-100 population doublings with OCI lines, with no decrease in cell growth rate. At this point we ended long-term cell growth experiments, thus the upper limit of population doublings that can be achieved is likely to be much greater with OCI lines (**Figure 1 d**). Sixty population doublings would be

approximately equal to 10^{20}-fold expansion in cell numbers (~ 100 quintillion cells) more than adequate for any research use.

The growth rate plateau that is seen during the culture of tumor cells in standard media is linked to the long lag time between the initial plating of tumor tissue and the emergence of a cell line. This is a significant variable in evaluating the efficiency and practicality of a culture system, and has significant implications for the quality of the cell lines. For example, Verschraegen *et al.,* reported that on average it took more than five months (21 weeks) before tumor cells could be passaged for the first time, which is similar to our experience using RPMI medium (**Figure 1 a**) [56].

In standard cell culture medium both normal and ovarian tumor cells are growth arrested within the initial several passages (**Figure 1**). Since the growth arrest due to telomere-shortening occurs typically after 50-70 passages, this type of early growth arrest is due to inadequate culturing conditions [57].

SUPPLEMENTARY METHODS

Soft agar colony assay

In order to confirm that the OCI cell lines we established maintained their transformed phenotype in culture we carried out anchorage independent growth assays in soft agar. Since normal cells are incapable of forming soft agar colonies, this is an excellent method to ensure that we have indeed established tumor cell lines. For these assays, well bottoms of a 12-well plate are sealed with 0.6% agar prepared in OCMI medium to prevent monolayer formation. Cells from established cultures (passage 6-8) are harvested. A single cell suspension in 0.4% agar in OCMI medium is added and

allowed to set at room temperature, and placed in 37℃ incubators with 5% CO_2. The cells are fed with 0.4% agar in OCMI at 2 weeks, and colony formation is assessed 2-4 weeks after plating.

Alternatively, tumor cells were grown in suspension cultures. The tumor spheres were grown in OCMI medium with 2% B27 supplement (Gibco), 20 ng/ml EGF, 20 ng/ml bFGF (BD Biosciences), 4 ug/ml heparin, and 0.5% methyl cellulose. For sphere formation experiments, 15,000-20,000 cells/well were plated into 6-well ultra-low attachment plates (Corning), and fresh medium was added on 1, 3,5, and spheres were counted at day 7.

LOH Analysis

The genomic DNA of tumor tissues were extracted from paraffin sections or, when available, from fresh tissues. The fresh tumor tissues were homogenized directly in RLT+ cell lysis buffer (Qiagen). The DNA was extracted from the lysates using the Qiagen All-Prep mini kit. Briefly, DNA is cleaved with Sty1, and the fragments are PCR amplified. The purified products were further fragmented with DNaseI, biotinylated, hybridized to a chip, and fluorescently labeled with phycoerythrin-conjugated streptavidin with signal amplification. Inferred LOH analysis in Figure 2 was performed using dCHIP software and employed the hidden Markov model with a reference heterozygosity rate of 0.2.

LOH segment analyses in Supplementary Fig 2a and Supplementary Data 1 was performed using the Affymetrix Genotyping Console (version 4.1.4.840). The BRLMM algorithm was used for genotyping (score threshold=0.5, prior size=10,000, DM

threshold=0.17). Unpaired sample analysis was performed for CN and LOH using 20 female samples taken from the HapMap samples that Affymetrix has provided for the platform (default configuration, i.e. quantile normalization, 0.1Mb genomic smoothing). The Segment Reporting Tool within the software was run to get the filtered result (minimum number of markers per segment=5, minimum genomic size of a segment=100kbp). Finally, after synchronizing the probe sets for all the samples, we further summarized the LOH calls for every 60kbp region along the chromosomes. When there were no LOH calls in such a region for all samples, the region was excluded from the final table. The complete dataset is available at GEO, accession number GSE40787.

Copy Number Analysis

The copy number analysis in Supplementary Fig. 9 was performed using the Molecular Inversion Probe (MIP) 330k microarrays from Affymetrix. The basic MIP assay has been previously described[58-61]. MIP probes are oligonucleotides in which the two end sequences are complementary to two adjacent genomic sequences; these two ends anneal to the genomic DNA in an inverted fashion with a single base between them. In copy number analysis the genomic DNA is hybridized to the MIP probe and the reaction split into two separate tubes containing nucleotide mixes (triphosphates of either Adenine + Thymine or Cytosine + Guanine). With the addition of polymerase and ligase, the MIP probe circulates in the presence of the nucleotide complementary to the allele on the genome. Genomic DNA is limiting in the reaction such that the number of circulated probes proportionally reflects the absolute amount of template DNA. After circularization, unused probes and genomic DNA are eliminated from the reaction by

exonuclease leaving only circularized probes. These probes are then amplified, labeled, detected, and quantified by hybridization to tag microarrays; tags are designed to have low cross hybridization. The data was analyzed using the Nexus 5.1 software from BioDiscovery.

In order to compare CNV patterns of OCI cells with the ovarian tumors in the TCGA dataset, we downloaded the MSKCC Agilent 1M Copy Number Variation data from the TCGA data portal (https://tcga-data.nci.nih.gov/docs/publications/ov_2011/). This set included 497 copy-number segmentation files generated from 487 TCGA ovarian samples using the CBS algorithm. We randomly selected 100 files and merged individual copy number profile into a single consensus using an interval-merging algorithm that sums together the mean log2 intensity values in overlapping intervals.

Similarly, we generated individual copy number profile for 25 ovarian cell-line samples using Affymetrix MIP array and the CBS algorithm in the DNAcopy package in R bioconductor. A consensus copy number profiling was generated from these 25 samples using the same interval-merging algorithm method for Copy Number/LOH.

RNA expression analysis

Total RNA was extracted from each cell line in triplicate (different passages from the same cell line) using the RNeasy Mini kit (Qiagen, Valencia, CA) according to the manufacturer's instructions. RNA was checked with a size fractionation procedure using a capillary electrophoresis instrument (Bioanalyzer 2100, Agilent Technologies, Santa Clara, CA) to ensure high quality and RNA concentrations were estimated using the Nanodrop ND-1000 (Nanodrop Technologies Inc, Wilmington, DE). Gene expression for the cell lines was measured using the Illumina HumanHT-12 v4 Expression BeadChip

platform. Raw signals of all the built-in controls were checked as quality control for the performance of the arrays. The sample-independent controls were used to check hybridization and signal generation and the housekeeping genes were used as sample-dependent controls. After background subtraction, the data were normalized across arrays using quantile normalization [62]. The average signal intensities were used for gene expression profiling.

Gene expression data for 285 ovarian tumor samples were also obtained from the Gene Expression Omnibus (GEO) accession number GSE9899. The samples were assayed using the Affymetrix HG-U133 Plus 2.0 platform. The data were normalized by RMA method [63]. The two data sets were combined by matching gene symbols. The data were median-centered for each sample. Genes with an expression level that had at least a 2-fold difference relative to the median value across tissues in at least 4 cell lines were selected. This resulted in 3831 genes for further analysis.

OCI cell line vs. human tumor mRNA expression clustering analysis in Figure 6

In Figure 6 the combined samples were clustered using hierarchical clustering to see whether the cell lines could be grouped with patient samples with different clinical outcomes. This was done using a Spearman correlation coefficient based distance matrix and Ward's minimum variance based agglomeration algorithm. The sample tree was cut into three main branches (**Figure 6a**). Cluster P2 has all the SOC cell lines (and a few OCI cell lines), and all the OCI cell lines were grouped in Cluster P1. Branch 3 was not included in survival analysis because it is small and has no cell line samples. The Kaplan-Meier curves for progression free survival and overall survival were plotted

in Figure 6b. All the statistical analysis, after the raw data had been generated from the platform vendor software, was performed in R [64].

Comparison of the mRNA expression of cell lines with primary tumor tissue is challenging, because primary tumors are a heterogeneous mix of normal cells, tumor cells, stromal cell, inflammatory cells, apoptotic cells, blood vessels, necrotic matrix etc. Furthermore, cell lines in culture have a much higher fraction of cells in cycle compared to tumors. Hence, comparisons of tissues with cell lines generally result in cell cycle, stroma, matrix, and inflammatory genes dominating the profile. Since we had limited fresh tumor material that was mostly used for optimizing culture conditions, we did not have enough tissue material to carry out microdissection that may address some of these problems. For these reason we were not able to compare the primary tumor tissue with cell lines.

OCI cell lines vs. TCGA/AOCS mRNA expression analysis in Figure 8

For the analysis in **Figure 8**, the gene expression microarray data for the Ince et al., ovarian cancer cell lines were downloaded from the Gene Expression Omnibus (GSE40785). In total, 37 microarrays and 47,323 unique probes were retrieved. All data were log2 transformed with Microsoft Excel 2010, quantile normalized with R statistical software version 3.1 with the package ‘ArrayTools’. The data were then filtered with Cluster v3.0 and all probes with standard deviations of less than 0.5 across all samples were removed, leaving 7,479 probes. These 7,479 probes from the 37 arrays were then median centered in Cluster v3.0 and hierarchical clustered. This information was then visualized with Java Treeview v.1.16r4 where two major groups of ovarian cancer cell

lines were observed. To identify the genes that were most differentially expressed across these two groups Significance Analysis of Microarrays in R with the same package. The top 500 probes over-expressed in each group (all which had a false discovery rate of <5) termed Ince Cluster 1 and Ince Cluster 2 were then used to generate gene signature scores for each tumor in The Cancer Genome Atlas (TCGA) ovarian cancer gene expression dataset. Signature scores for other relevant gene expression signatures were also identified for each tumor in the TCGA dataset including the TCGA defined 'poor' and 'good' prognosis genes, the Australian Ovarian Cancer Study (AOCS, Tothill) C1-C6 'up' genes, and a recently described 'vascular content' gene expression signature. All signature scores were identified by taking the mean value for all genes within a respective signature. Excel was then used to identify the Pearson correlation values for each of the signature scores (Figure 1A). This analysis showed a very strong correlation with the genes most abundantly expressed in the Ince Cluster 1 ovarian cell lines and the AOCS Tothill C1 up genes (value = 0.93). Interestingly, both these signatures also exhibited a strong correlation with genes that are abundantly expressed in vascular endothelial cells (>0.7), suggesting that both of these signatures define ovarian cancer cells with mesenchymal/endothelial attributes. Next, the Tothill gene expression microarray data (GSE9899) and clinical data were downloaded and processed as above. Since the AOCS Tothill paper did not define which tumors they identified as belonging to groups C1-C6, we next aimed to categorize the 295 AOCS Tothill samples into one of six groups. Therefore, we used the k-Means function of Cluster v3.0, with similarity metric Euclidean distance, and ran 1000 permutations to identify these sample groupings. The Kaplan-Meier plot (Figure 8) for

overall survival was then generated with R using package Biobase and in Excel with Winstat.

Methods used for pathway enrichment analysis in Supplementary Fig. 10-11

For pathway analysis in Supplementary Figure 10-11 and Supplementary Data 2 we used average value of expressions in log2 transformed microarray data, for each gene on each sample, detected by different probes to denote the consensus gene expression. In MATLAB 2010b, student's t-test p-value and fold-change value were calculated for each gene with the partition of clusters 1 and 2. Gene names and their affiliated p values, fold-change values were imported into Ingenuity Pathway Analysis (IPA). By setting cutoffs as 0.05/N (N=4308, the total number of genes) and 1 for p and fold-change values (log2-based, either up- or down-regulation), 823 genes were identified as significantly differentiate expressed. 558 and 265 genes were found up-regulated in clusters 1 and 2 respectively. Using the 'core analysis' module in IPA, 37 and 41 pathways were found significantly enriched (with the p value < 0.05 by IPA) correspondingly for cluster 1 and 2 as up-regulated. Enriched pathways and related genes for cluster 1 and 2 were shown separately in Supplementary figures 3A and 3B.

Protein expression analysis in Figure 3, 5, 7 and Supplementary Fig. 12-16

For the RPPA analysis in Figure 3 the cell lysates were immobilized on nitrocellulose coated slides, and each slide was incubated with an antibody specific for a protein of interest. The protein lysates were prepared in a lysis buffer containing SDS and protease inhibitors. Semi-confluent wells in 6-well plates were lysed in 125 uL lysis buffer on ice in triplicate (at least two different passages from the same cell line). Sample concentrations were adjusted after BCA measurements. Each sample was

spotted onto the slide in dilution series (5 dilutions), and the slides were probed with 156 (first experiment) and 191 (second experiment) primary antibodies (see Supplementary Data 5 for detailed antibody information) and the signal intensity was captured by a biotin conjugated secondary antibody and amplified by a DakoCytomation-catalyzed system. The slides were scanned, analyzed and quantitated using MicroVigene software (Vigene Tech inc. Carlise, MA) to generate spot signal intensities, which were processed by the R package SuperCurve [65].

Protein concentrations were derived from the supercurve for each lysate by curve-fitting and normalized by median polish. The antibodies utilized in this study were primarily targeting proteins involved in PI3K/Akt pathway or were otherwise cancer related signaling pathways. The signal intensity data was collected and normalized using software specifically developed for RPPA analyses (http://www.vigenetech.com). Replicate data were averaged, log2-median centered, hierarchically clustered (Cluster 3.0), and visualized in heatmaps (Java TreeView 1.1.1). Two-sided Student's t tests of log transformed RPPA values were performed using the t.test function in bioConductor/R.

Western blots in Supplementary Figure 16 were probed with the following antibodies WT1 (ab89901, 1:500, abcam) B-Actin (A2228,1:5,000, Sigma), CK7 (MAB3554, 1:200, Millipore), p16INK4a (554079, 1:50, BD PharMingen), Pax8 (10336-1-AP, 1:500, Proteintech), ER (SC8002 - F10, 1:200, Santa Cruz Biotechnology) and HNF1b (HPA002083, 1:250, Sigma). Protein loading per lane: 25ug, incubation conditions: 4°C for 12h.

IHC was performed using the following probing conditions: WT1 (M3561, 1:100, DAKO), CK7 (Ab117238, 1:100, abcam), p16INK4a (ab54210, 1:200, abcam), Pax8 (10336-1-AP, 1:800, Proteintech), ER (SC8002 - F10, 1:100, Santa Cruz Biotechnology) and HNF1b (HPA002083, 1:100, Sigma), p53 (1767, 1:200, Immunotech) and ARID1a (ab176395, 1:200, abcam).

Cell Line Unique Identifier mtDNA and STR in Supplementary Tables 5-7

A common problem in cell culture is cross-contamination or misidentification of cells. In repeated studies since 1970s, it has been shown that 15-25 % of cell lines are contaminated with a second line, or is completely misidentified (Alston-Roberts, 2010). In the 1970s and 1980s, Nelson-Rees showed that over 100 cancer cell lines were actually HeLa cells [66,67]. An effective cell culture quality and identity control is required in order to avoid inter- and intra-species contamination of cell lines and their further propagation and dissemination. However, vigilant monitoring against misidentification and cross-contamination is possible by developing a practical **"unique identifier"** for the cells by the establishing laboratory.

In Supplementary Table 5 and 6 we present mitochondrial DNA (mtDNA sequence) and short tandem repeat (**STR**) evidence that the cell lines examined in this manuscript are from unrelated individuals. Thus, the OCI cell lines can be verified by the recipient laboratories and can be monitored for purity and integrity. This will significantly reduce the incidence of cell line contamination and misidentification[68]. The control region of the human mtDNA is highly polymorphic due to a rapid rate of evolution. The mtDNA does not undergo recombination and is present in high copy number per cell. For this reason, its analysis is very useful for the identification of cell lines.

For mtDNA analysis the cell line DNA was extracted using the QIAamp DNA Mini Kit using standard methods. The HVRI and HVRII segments were amplified by PCR using specific primers: Primer R: 5′-GAA TCG GAG GAC AAC CAG TA-3′ and Primer F: 5′-TGA TGT GAG CCC GTC TAA AC-3′ [69]. The two segments were directly sequenced by capillary electrophoresis on both strands. Nucleotide substitutions and insertions/deletions were found by comparison with Cambridge reference sequence (NCBI Reference Sequence NC_012920.1) [70]. PCR amplification was performed in 50 µl with a Bio-Rad thermocycler (Applied Biosystems Inc., USA). The PCR product amplified from D-loop mtDNA was detected by electrophoresis on a 1% agarose gel with 1X TBE buffer at 120 V and 60mA for 60 min and under UV transillumination after ethidium bromide staining, and photographed. After purification with the QIAquick Gel Extraction Kit (QIAGEN, USA), all of the PCR products were sequenced (Operon, Petaluma, CA) in both directions using the same primers as PCR [69]. After nucleotide sequencing, sequence variations were determined by comparison with the Cambridge reference sequence using CLUSTALW2 [71].

In Supplementary Table 7 the OCI cell lines were validated by STR DNA fingerprinting using the Promega 16 High Sensitivity STR Kit (Catalog # DC2100). The STR profiles were compared to online search databases (DSMZ/ATCC/JCRB/RIKEN) of 2455 known profiles; along with the MD Anderson Characterized Cell Line Core (CCLC) database of 2556 know profiles. The STR DNA fingerprinting was carried out by a by the MD Anderson Cancer Center Support Grant-funded Characterized Cell Line core (NCI # CA016672). All of the tested OCI cell lines have unique profile with no match in these reference datasets, except, OCI-C2p which had a match with the breast cancer cell line

UACC2715. This match appears to be incidental because the SNP/LOH profile of OCI-C2p has a > 95% identity with its matching uncultured original tumor. All the SOC lines used in this study were also STR validated and matched the unique reference profile listed by ATCC for each line (data not shown).

mRNA profile of high grade serous vs. clear cell ovarian carcinomas

To determine if the OCI-CC and OCI-HGS cell lines reflect gene expression differences that are found within patients with CCOC and HGSOC, we identified a mRNA expression signature that significantly separated the CCOC and HGSOC tumors from Wu et al [72]._Signature values for each OCI cell line were then identified, box-and-whisker plot were generated, and ANOVA identified that the same genetic discriminator signature that was identified in patient tumors was also present in the OCI lines.

The gene expression microarray data from Wu *et a*l., [72] were downloaded from the Gene Expression Omnibus (GSE6008). R software version 3.1 was then used to quantile normalize the data, collapse probes, and median center the data. Significance Analysis of Microarrays [73] was performed in Microsoft Excel 2010. The two-class unpaired SAM performed aimed to identify the genes most different in the 8 Wu et al defined clear cell carcinomas versus the 17 grade 3 serous ovarian carcinomas. The top 25 significantly different mRNAs were defined by the highest SAM score (T-statistic value) and False Discovery Rate (0) that were also present within the Ince *et al.*, OCI cell line dataset. These 25 mRNAs were used to generate a Clear Cell discriminator gene list. The values of each gene were averaged to generate signature scores for each tumor (Wu dataset) or cell line dataset (Ince dataset). Box-and-whisker plot were

generated, and ANOVA identified that the same genetic discriminator signature that was identified in patient tumors was also present in the OCI lines

Drug sensitivity experiments

The relative sensitivities of OCI and SOC cell lines to Taxol was measured by seeding 3000 cells/well in six replicates in 96-well black-walled clear bottom Corning plates and allowing attachment in OCMI for 12h. Both OCI and SOC cell lines were cultured in the presence of Taxol dosages ranging from 1 to 800nM (or vehicle control) in OCMI medium for 120h. The fraction of metabolically active cells after drug treatment was measured by incubation with 2:10 (v/v) CellTiter-Blue reagent (Promega Cat# G8081) in media for 2h, and the reaction was stopped by addition of 3% SDS. Fluorescence was measured in SpectraMax M5 plate reader (Molecular Devices, CA) using SoftMax software (555EX/585EM). In case of high variation among calculated values for four independent assays, four additional independent assays were performed to allow defining and discarding outliers.

Lethal Dose Analysis:

Data was analyzed using GraphPad Prism 5 Software, and values were fit to a dose response-inhibition curve with variable slope (sigmoidal with four parameters). The cell viability as response r between bottom (B) and top (T) values ($B < r < T$) was assumed to depend on concentration (C) via a general Hill equation for inhibition as in equation (1)

$$(1)\quad r = B + (T - B)\frac{C^n}{C^n + IC_{50}^n}$$

where IC_{50} is the concentration producing a response that is halfway between Bottom and Top (notation as used in Prism) and n is the Hill coefficient. T was constrained to be constant and equal to 100, B to be equal or greater than zero and n to be less than 4. Accordingly, the concentration that produces a given response r (viability) can be calculated from equation (2), and after obtaining B, T, IC_{50}, and n from fitting the data, this was used to estimate LD_{90} values corresponding to concentrations causing 90% lethality (r = 10% viable cells).

$$(2)\quad C_r = IC_{50}\left(\frac{r-B}{T-r}\right)^{1/n}$$

SUPPLEMENTARY REFERENCES

1 Watanabe, K. *et al.* A ROCK inhibitor permits survival of dissociated human embryonic stem cells. *Nature biotechnology* 25, 681-686, doi:10.1038/nbt1310 (2007).

2 Liu, X. *et al.* ROCK inhibitor and feeder cells induce the conditional reprogramming of epithelial cells. *The American journal of pathology* 180, 599-607, doi:10.1016/j.ajpath.2011.10.036 (2012).

3 Bueno, C., Montes, R. & Menendez, P. The ROCK inhibitor Y-27632 negatively affects the expansion/survival of both fresh and cryopreserved cord blood-derived CD34+ hematopoietic progenitor cells: Y-27632 negatively affects the expansion/survival of CD34+HSPCs. *Stem cell reviews* 6, 215-223, doi:10.1007/s12015-010-9118-5 (2010).

4 Lamas, N. J., Serra, S. C., Salgado, A. J. & Sousa, N. Failure of Y-27632 to improve the culture of adult human adipose-derived stem cells. *Stem cells and cloning : advances and applications* 8, 15-26, doi:10.2147/SCCAA.S66597 (2015).

5 Routhier, A. *et al.* Pharmacological inhibition of Rho-kinase signaling with Y-27632 blocks melanoma tumor growth. *Oncology reports* 23, 861-867 (2010).

6 Kobel, M. *et al.* Ovarian carcinoma histotype determination is highly reproducible, and is improved through the use of immunohistochemistry. *Histopathology* 64, 1004-1013, doi:10.1111/his.12349 (2014).

7 Liliac, L. *et al.* The value of PAX8 and WT1 molecules in ovarian cancer diagnosis. *Romanian journal of morphology and embryology = Revue roumaine de morphologie et embryologie* 54, 17-27 (2013).

8 Harrell, J. C. *et al.* Endothelial-like properties of claudin-low breast cancer cells promote tumor vascular permeability and metastasis. *Clinical & experimental metastasis* 31, 33-45, doi:10.1007/s10585-013-9607-4 (2014).

9 Sangrajrang, S. & Fellous, A. Taxol resistance. *Chemotherapy* 46, 327-334 (2000).

10 Orr, G. A., Verdier-Pinard, P., McDaid, H. & Horwitz, S. B. Mechanisms of Taxol resistance related to microtubules. *Oncogene* 22, 7280-7295, doi:10.1038/sj.onc.1206934 (2003).

11 Murphy, L., Henry, M., Meleady, P., Clynes, M. & Keenan, J. Proteomic investigation of taxol and taxotere resistance and invasiveness in a squamous lung carcinoma cell line. *Biochimica et biophysica acta* 1784, 1184-1191, doi:10.1016/j.bbapap.2008.04.014 (2008).

12 L'Esperance, S. *et al.* Gene expression profiling of paired ovarian tumors obtained prior to and following adjuvant chemotherapy: molecular signatures of chemoresistant tumors. *International journal of oncology* 29, 5-24 (2006).

13 Aissat, N. *et al.* Antiproliferative effects of rapamycin as a single agent and in combination with carboplatin and paclitaxel in head and neck cancer cell lines. *Cancer chemotherapy and pharmacology* 62, 305-313, doi:10.1007/s00280-007-0609-2 (2008).

14 Liu, Y., Chen, L., Ko, T. C., Fields, A. P. & Thompson, E. A. Evi1 is a survival factor which conveys resistance to both TGFbeta- and taxol-mediated cell death via PI3K/AKT. *Oncogene* 25, 3565-3575, doi:10.1038/sj.onc.1209403 (2006).

15 Zhang, H., Li, B., Bai, S. W. & Wang, H. J. Constitutively active Akt contributes to vincristine resistance in human retinoblastoma cells. *Cancer Invest* 28, 156-165, doi:10.3109/07357900903179641 (2010).

16 Bava, S. V. *et al.* Akt is upstream and MAPKs are downstream of NF-kappaB in paclitaxel-induced survival signaling events, which are down-regulated by curcumin contributing to their synergism. *The international journal of biochemistry & cell biology* 43, 331-341, doi:10.1016/j.biocel.2010.09.011 (2011).

17 Shin, S. *et al.* Activator protein-1 has an essential role in pancreatic cancer cells and is regulated by a novel Akt-mediated mechanism. *Molecular cancer research : MCR* 7, 745-754, doi:10.1158/1541-7786.MCR-08-0462 (2009).

18 Ueda, T., Bruchovsky, N. & Sadar, M. D. Activation of the androgen receptor N-terminal domain by interleukin-6 via MAPK and STAT3 signal transduction pathways. *The Journal of biological chemistry* 277, 7076-7085, doi:10.1074/jbc.M108255200 (2002).

19 Zhou, J. *et al.* Paclitaxel-resistant human ovarian cancer cells undergo c-Jun NH2-terminal kinase-mediated apoptosis in response to noscapine. *The Journal of biological chemistry* 277, 39777-39785, doi:10.1074/jbc.M203927200 (2002).

20 Zhang, X., Wrzeszczynska, M. H., Horvath, C. M. & Darnell, J. E., Jr. Interacting regions in Stat3 and c-Jun that participate in cooperative transcriptional activation. *Molecular and cellular biology* 19, 7138-7146 (1999).

21 Houck, S. A. & Clark, J. I. Dynamic subunit exchange and the regulation of microtubule assembly by the stress response protein human alphaB crystallin. *PLoS One* 5, e11795, doi:10.1371/journal.pone.0011795 (2010).

22 Lu, M., Xiao, L., Hu, J., Deng, S. & Xu, Y. Targeting of p38 mitogen-activated protein kinases to early growth response gene 1 (EGR-1) in the human paclitaxel-resistance ovarian carcinoma cells. *J Huazhong Univ Sci Technolog Med Sci* 28, 451-455, doi:10.1007/s11596-008-0417-x (2008).

23 Halder, J. *et al.* Focal adhesion kinase silencing augments docetaxel-mediated apoptosis in ovarian cancer cells. *Clinical cancer research : an official journal of the American Association for Cancer Research* 11, 8829-8836, doi:10.1158/1078-0432.CCR-05-1728 (2005).

24 Yang, J. S. *et al.* A role for phosphatidic acid in COPI vesicle fission yields insights into Golgi maintenance. *Nature cell biology* 10, 1146-1153, doi:10.1038/ncb1774 (2008).

25 McDaid, H. M. *et al.* Enhancement of the therapeutic efficacy of taxol by the mitogen-activated protein kinase kinase inhibitor CI-1040 in nude mice bearing human heterotransplants. *Cancer research* 65, 2854-2860, doi:10.1158/0008-5472.CAN-04-4391 (2005).

26 Bauer, J. A. *et al.* RNA interference (RNAi) screening approach identifies agents that enhance paclitaxel activity in breast cancer cells. *Breast Cancer Res* 12, R41, doi:10.1186/bcr2595 (2010).

27 Rosano, L. *et al.* Acquisition of chemoresistance and EMT phenotype is linked with activation of the endothelin A receptor pathway in ovarian carcinoma cells. *Clinical cancer research : an official journal of the American Association for Cancer Research* 17, 2350-2360, doi:10.1158/1078-0432.CCR-10-2325 (2011).

28 Samarakoon, R., Higgins, C. E., Higgins, S. P. & Higgins, P. J. Differential requirement for MEK/ERK and SMAD signaling in PAI-1 and CTGF expression in response to microtubule disruption. *Cellular signalling* 21, 986-995, doi:10.1016/j.cellsig.2009.02.007 (2009).

29 Buttiglieri, S. *et al.* Role of Pax2 in apoptosis resistance and proinvasive phenotype of Kaposi's sarcoma cells. *The Journal of biological chemistry* 279, 4136-4143, doi:10.1074/jbc.M306824200 (2004).

30 Akiyama, T. *et al.* Purified protein kinase C phosphorylates microtubule-associated protein 2. *The Journal of biological chemistry* 261, 15648-15651 (1986).

31 Priulla, M. *et al.* Preferential chemosensitization of PTEN-mutated prostate cells by silencing the Akt kinase. *Prostate* 67, 782-789, doi:10.1002/pros.20566 (2007).

32 Kashkin, K. N. *et al.* Genes potentially associated with resistance of lung cancer cells to paclitaxel. *Dokl Biochem Biophys* 437, 105-108, doi:10.1134/S1607672911020153 (2011).

33 Dong, C., Li, Z., Alvarez, R., Jr., Feng, X. H. & Goldschmidt-Clermont, P. J. Microtubule binding to Smads may regulate TGF beta activity. *Molecular cell* 5, 27-34 (2000).

34 Zhang, Y., Feng, X. H. & Derynck, R. Smad3 and Smad4 cooperate with c-Jun/c-Fos to mediate TGF-beta-induced transcription. *Nature* 394, 909-913, doi:10.1038/29814 (1998).

35 Le, X. F. & Bast, R. C., Jr. Src family kinases and paclitaxel sensitivity. *Cancer Biol Ther* 12, 260-269 (2011).

36 Chen, T., Pengetnze, Y. & Taylor, C. C. Src inhibition enhances paclitaxel cytotoxicity in ovarian cancer cells by caspase-9-independent activation of caspase-3. *Molecular cancer therapeutics* 4, 217-224 (2005).

37 Cao, X., Tay, A., Guy, G. R. & Tan, Y. H. Activation and association of Stat3 with Src in v-Src-transformed cell lines. *Molecular and cellular biology* 16, 1595-1603 (1996).

38 Choi, H. J., Lee, J. H., Park, S. Y., Cho, J. H. & Han, J. S. STAT3 is involved in phosphatidic acid-induced Bcl-2 expression in HeLa cells. *Exp Mol Med* 41, 94-101 (2009).

39 Ferlini, C. *et al.* Bcl-2 down-regulation is a novel mechanism of paclitaxel resistance. *Molecular pharmacology* 64, 51-58, doi:10.1124/mol.64.1.51 (2003).

40 Garcia, R. *et al.* Constitutive activation of Stat3 by the Src and JAK tyrosine kinases participates in growth regulation of human breast carcinoma cells. *Oncogene* 20, 2499-2513, doi:10.1038/sj.onc.1204349 (2001).

41 Hawthorne, V. S. *et al.* ErbB2-mediated Src and signal transducer and activator of transcription 3 activation leads to transcriptional up-regulation of p21Cip1 and chemoresistance in breast cancer cells. *Molecular cancer research : MCR* 7, 592-600, doi:10.1158/1541-7786.MCR-08-0316 (2009).

42 Ng, D. C., Lim, C. P., Lin, B. H., Zhang, T. & Cao, X. SCG10-like protein (SCLIP) is a STAT3-interacting protein involved in maintaining epithelial morphology in MCF-7 breast cancer cells. *The Biochemical journal* 425, 95-105, doi:10.1042/BJ20091213 (2010).

43 Park, S. *et al.* Molecular cloning and characterization of the human AKT1 promoter uncovers its up-regulation by the Src/Stat3 pathway. *The Journal of biological chemistry* 280, 38932-38941, doi:10.1074/jbc.M504011200 (2005).

44 Zhang, Y. *et al.* Activation of Stat3 in v-Src-transformed fibroblasts requires cooperation of Jak1 kinase activity. *The Journal of biological chemistry* 275, 24935-24944, doi:10.1074/jbc.M002383200 (2000).

45 Shen, D. W., Pouliot, L. M., Hall, M. D. & Gottesman, M. M. Cisplatin resistance: a cellular self-defense mechanism resulting from multiple epigenetic and genetic changes. *Pharmacological reviews* 64, 706-721, doi:10.1124/pr.111.005637 (2012).

46 Ince, T. A. *et al.* Transformation of different human breast epithelial cell types leads to distinct tumor phenotypes. *Cancer cell* 12, 160-170, doi:10.1016/j.ccr.2007.06.013 (2007).

47 Sauder, C. A. *et al.* Phenotypic plasticity in normal breast derived epithelial cells. *BMC cell biology* 15, 20, doi:10.1186/1471-2121-15-20 (2014).

48 Brenneman, M. & Rigby, P. Protein electrophoretic patterns of serum and peritoneal fluid in normal, tumor-bearing, and immune mice. *Cancer research* 28, 1138-1142 (1968).

49 Nagy, J. A. *et al.* Permeability properties of tumor surrogate blood vessels induced by VEGF-A. *Lab Invest* 86, 767-780, doi:10.1038/labinvest.3700436 (2006).

50 Mills, G. B., May, C., McGill, M., Roifman, C. M. & Mellors, A. A putative new growth factor in ascitic fluid from ovarian cancer patients: identification, characterization, and mechanism of action. *Cancer research* 48, 1066-1071 (1988).

51 Kumar, S., Lata, K., Mukhopadhyay, S. & Mukherjee, T. K. Role of estrogen receptors in pro-oxidative and anti-oxidative actions of estrogens: a perspective. *Biochimica et biophysica acta* 1800, 1127-1135, doi:10.1016/j.bbagen.2010.04.011 (2010).

52 Sato, T. *et al.* Long-term expansion of epithelial organoids from human colon, adenoma, adenocarcinoma, and Barrett's epithelium. *Gastroenterology* 141, 1762-1772, doi:10.1053/j.gastro.2011.07.050 (2011).

53 Kamb, A. What's wrong with our cancer models? *Nature reviews. Drug discovery* 4, 161-165, doi:10.1038/nrd1635 (2005).

54 Jacquez, J. A. & Barry, E. Tissue culture media; the essential non-dialyzable factors in human placental cord serum. *J Gen Physiol* 34, 765-774 (1951).

55 Danes, B. S. Suspension cultures of strain L mouse fibroblasts. I. A glass stirrer apparatus for the cultivation of cell suspensions. *Experimental cell research* 12, 169-179 (1957).

56 Verschraegen, C. F. *et al.* Establishment and characterization of cancer cell cultures and xenografts derived from primary or metastatic Mullerian cancers. *Clinical cancer research : an official journal of the American Association for Cancer Research* 9, 845-852 (2003).

57 Kuilman, T., Michaloglou, C., Mooi, W. J. & Peeper, D. S. The essence of senescence. *Genes & development* 24, 2463-2479, doi:10.1101/gad.1971610 (2010).

58 Hardenbol, P. *et al.* Multiplexed genotyping with sequence-tagged molecular inversion probes. *Nature biotechnology* 21, 673-678, doi:10.1038/nbt821 (2003).

59 Hardenbol, P. *et al.* Highly multiplexed molecular inversion probe genotyping: over 10,000 targeted SNPs genotyped in a single tube assay. *Genome Res* 15, 269-275, doi:10.1101/gr.3185605 (2005).

60 Ji, H. *et al.* Molecular inversion probe analysis of gene copy alterations reveals distinct categories of colorectal carcinoma. *Cancer research* 66, 7910-7919, doi:10.1158/0008-5472.CAN-06-0595 (2006).

61 Karlin-Neumann, G., Sedova, M., Sapolsky, R., Forman, J., Wang, Y., Moorhead, M., Faham, M. in *Genetic Variation: a Laboratory Manual* (ed Gabriel SB Weiner MP, Stephens JC) 199-211 (2007).

62 Bolstad, B. M., Irizarry, R. A., Astrand, M. & Speed, T. P. A comparison of normalization methods for high density oligonucleotide array data based on variance and bias. *Bioinformatics* 19, 185-193 (2003).

63 Irizarry, R. A. *et al.* Summaries of Affymetrix GeneChip probe level data. *Nucleic acids research* 31, e15 (2003).

64 Team, R. D. C. R: A language and environment for statistical computing. R Foundation for Statistical Computing. (2011).

65 Hu, J. *et al.* Non-parametric quantification of protein lysate arrays. *Bioinformatics* 23, 1986-1994, doi:10.1093/bioinformatics/btm283 (2007).

66 Nelson-Rees, W. A., Daniels, D. W. & Flandermeyer, R. R. Cross-contamination of cells in culture. *Science* 212, 446-452 (1981).

67 Nelson-Rees, W. A. & Flandermeyer, R. R. HeLa cultures defined. *Science* 191, 96-98 (1976).

68 Gignac, S. M. *et al.* Multiparameter approach in the identification of cross-contaminated leukemia cell lines. *Leukemia & lymphoma* 10, 359-368, doi:10.3109/10428199309148561 (1993).

69 Peng, Z. *et al.* Sequence variations of mitochondrial DNA D-loop region are associated with familial nasopharyngeal carcinoma. *Mitochondrion* 11, 327-333, doi:10.1016/j.mito.2010.12.008 (2011).

70 Andrews, R. M. *et al.* Reanalysis and revision of the Cambridge reference sequence for human mitochondrial DNA. *Nature genetics* 23, 147, doi:10.1038/13779 (1999).

71 Larkin, M. A. *et al.* Clustal W and Clustal X version 2.0. *Bioinformatics* 23, 2947-2948, doi:10.1093/bioinformatics/btm404 (2007).

72 Wu, R. *et al.* Mouse model of human ovarian endometrioid adenocarcinoma based on somatic defects in the Wnt/beta-catenin and PI3K/Pten signaling pathways. *Cancer cell* 11, 321-333, doi:10.1016/j.ccr.2007.02.016 (2007).

73 Tusher, V. G., Tibshirani, R. & Chu, G. Significance analysis of microarrays applied to the ionizing radiation response. *Proceedings of the National Academy of Sciences of the United States of America* 98, 5116-5121, doi:10.1073/pnas.091062498 (2001).
